# BRCA genetic testing and counseling in breast cancer: how do we meet our patients’ needs?

**DOI:** 10.1038/s41523-024-00686-8

**Published:** 2024-09-05

**Authors:** Peter Dubsky, Christian Jackisch, Seock-Ah Im, Kelly K. Hunt, Chien-Feng Li, Sheila Unger, Shani Paluch-Shimon

**Affiliations:** 1 Breast and Tumor Center, Hirslanden Klinik St. Anna, Lucerne, Switzerland; 2 University of Lucerne, Faculty of Health Sciences and Medicine, Lucerne, Switzerland; 3 Department of Obstetrics and Gynecology, Breast and Gynecologic Cancer Center, Sana Klinikum Offenbach, Offenbach, Germany; 4 Seoul National University Hospital, Cancer Research Institute, Seoul National University College of Medicine, Seoul National University, Seoul, Republic of Korea; 5 MD Anderson Cancer Center, Houston, TX USA; 6 National Institute of Cancer Research, National Health Research Institutes, Tainan, Taiwan; 7 Genetica, Lausanne, Switzerland; 8 Hadassah University Hospital & Faculty of Medicine, Hebrew University, Jerusalem, Israel

**Keywords:** Breast cancer, Breast cancer, Genetic testing

## Abstract

*BRCA1* and *BRCA2* are tumor suppressor genes that have been linked to inherited susceptibility of breast cancer. Germline *BRCA1/2* pathogenic or likely pathogenic variants (gBRCAm) are clinically relevant for treatment selection in breast cancer because they confer sensitivity to poly(ADP-ribose) polymerase (PARP) inhibitors. *BRCA1/2* mutation status may also impact decisions on other systemic therapies, risk-reducing measures, and choice of surgery. Consequently, demand for gBRCAm testing has increased. Several barriers to genetic testing exist, including limited access to testing facilities, trained counselors, and psychosocial support, as well as the financial burden of testing. Here, we describe current implications of gBRCAm testing for patients with breast cancer, summarize current approaches to gBRCAm testing, provide potential solutions to support wider adoption of mainstreaming testing practices, and consider future directions of testing.

## Introduction

*BRCA1* and *BRCA2* were identified in the 1990s as genes linked to inherited susceptibility to breast cancer^1,2^. As tumor suppressor genes, they encode proteins that are crucial for the repair of complex DNA damage (such as double-strand breaks) by homologous recombination^3^. Germline mutations (i.e., pathogenic or likely pathogenic variants) in *BRCA1/2* (gBRCAm) affecting this vital DNA repair pathway predispose individuals to developing breast cancer by impairing homologous recombination and causing genomic instability^3^.

The advent of poly(ADP-ribose) polymerase (PARP) inhibitors has revolutionized the therapeutic landscape for cancers associated with gBRCAm, including breast, ovarian, prostate, and pancreatic cancer^4^. For breast cancer, the focus of this article, PARP inhibitors are approved for early and advanced disease harboring gBRCAm based on the results of major clinical trials: for olaparib, OlympiAD and OlympiA; and for talazoparib, EMBRACA^5–7^. Given the opportunity for therapeutic targeting of gBRCAm, timely determination of gBRCAm status is critical to guide treatment decisions, and demand for gBRCAm testing has rapidly increased in recent years^8^. High-throughput sequencing technologies have made analysis of cancer-susceptibility genes rapid and affordable^8^. However, there is concern that the demand for gBRCAm testing may overwhelm current genetic services^9^. Furthermore, barriers at the individual-, provider-, systems-, and policy-levels exist, which restrict access to genetic testing resources and genetic counseling^10^. Innovative methods of mainstreaming genetic services may help overcome some of these challenges. Education and resources to support appropriate counseling for gBRCAm testing, as well as information on the implications of testing, and models for genetic test consent, are urgently needed to support the evolving clinical space.

In this review, we describe the implications of gBRCAm testing for potential surgical approaches and treatment in patients with breast cancer, summarize the various approaches to gBRCAm testing (including traditional and alternative models), provide practical resources to support mainstreaming of the gBRCAm testing pathway, and consider the relevance of genetic testing in breast cancer in the future.

## Biology of BRCAm in breast cancer

Hereditary breast and ovarian cancer (HBOC) syndrome accounts for approximately 10% of breast cancer cases^11^. *BRCA1* and *BRCA2* are the main genes involved in genetic susceptibility to breast cancer^12^. HBOC is associated with early-onset breast cancer, and an increased risk of other cancers, including ovarian, pancreatic, fallopian tube, and prostate^3^. The cumulative lifetime risk of developing breast cancer by age 80 years is high at 72 and 69% for *BRCA1* and *BRCA2* mutation carriers, respectively^13^. Female gBRCAm carriers also have a 44% (*BRCA1*) and 17% (*BRCA2*) cumulative risk of developing ovarian cancer^13^.

Patients harboring gBRCAm are more likely to develop breast cancer at a younger age, with approximately 12% of the cases arising in women ≤40 years of age attributed to pathogenic or likely pathogenic variants in *BRCA1* or *BRCA2*^14^. These breast cancers have distinct biological features: among individuals with g*BRCA1*m, breast cancers are typically hormone receptor-negative (~76%) and human epidermal growth factor receptor 2 (HER2)-negative (94%), while breast cancers developing in individuals with g*BRCA2*m are more frequently hormone receptor-positive (83%) and HER2-negative (89%)^14^.

## Goals of gBRCAm testing in breast cancer

**Fig. 1:**
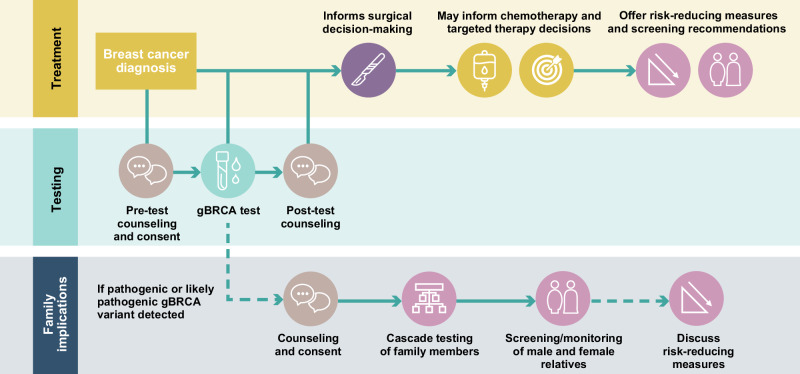
The pathway from gBRCAm testing to decisions relating to risk-reducing measures, choice of surgery, and systemic therapies.

Available evidence regarding surgical and systemic treatment outcomes in patients with gBRCAm breast cancer highlights the importance of determining gBRCAm status prior to finalizing treatment decisions. Clinical practice guidelines further reinforce the role of gBRCAm testing in the context of treatment decision-making, beyond its importance for risk management and cascade testing^11,15^. The presence of gBRCAm may impact decisions about risk-reducing measures, choice of surgery, and systemic therapies (Fig. 1).

### Surgical decision-making

#### Breast-conserving surgery (BCS)

BCS aims to remove the breast tumor, with clear margins, in a manner that is cosmetically acceptable to the patient^16^. Although BCS is recommended for most patients with early-stage operable breast cancer^15^, the best approach for patients harboring gBRCAm is unclear. Practice guidelines recommend that gBRCAm status should not preclude the use of BCS as a surgical option for breast cancer^17^. However, these patients should be counseled regarding the risk of ipsilateral breast cancer recurrence, new primary breast cancer in the treated breast, and contralateral breast cancer, noting that intensified surveillance is a reasonable treatment strategy for breast cancer^11,17^.

#### Contralateral risk-reducing mastectomy (CRRM)

Some women with a confirmed gBRCAm opt for CRRM over BCS, which is removal of the unaffected breast to reduce the risk of contralateral breast cancer, with or without the option of breast reconstruction^18^. A meta-analysis of outcomes in patients with gBRCAm found that CRRM reduced the relative risk of contralateral breast cancer by 93% versus surveillance and significantly increased overall survival (OS) versus surveillance^19^. It should be noted that benefit from CRRM was not maintained in all studies after adjusting for confounding factors^20^, and the absolute survival benefits of mastectomy (both ipsilateral and contralateral) are heavily dependent on patient prognosis; patients with aggressive types of disease, and especially those with little response from neoadjuvant systemic therapy regimens, are at higher risk from distant metastasis than local recurrence or a new primary in the contralateral breast.

#### Risk-reducing salpingo-oophorectomy (RRSO)

While RRSO is indicated in gBRCAm carriers, its effect on breast cancer risk reduction is not clear^21^. A recent systematic review and meta-analysis of 21,022 patients demonstrated a 37 and 49% reduction in the risk of developing breast cancer following RRSO compared with no RRSO in patients with g*BRCA1*m and g*BRCA2*m, respectively, with the effect particularly pronounced in younger women with gBRCAm^22^. A retrospective analysis in 676 women harboring gBRCAm showed that oophorectomy decreased mortality in patients with g*BRCA1*m and decreased breast cancer-specific mortality in patients with estrogen receptor (ER)-negative gBRCAm breast cancer^23^. Other studies have failed to demonstrate a benefit of RRSO on breast cancer risk^24,25^.

### Systemic treatment decision-making

#### Chemotherapy

gBRCAm advanced breast cancers are sensitive to platinum-based and non-platinum-based chemotherapy regimens^26–29^. For early breast cancer, patients with gBRCAm are treated with anthracycline/taxane-based regimens, similar to those individuals with sporadic breast cancers^30^. The clinical value of adding platinum therapy to neoadjuvant chemotherapy for patients with gBRCAm tumors is inconclusive. The phase 3 BrighTNess trial concluded that the addition of carboplatin, with or without veliparib, to neoadjuvant chemotherapy significantly improved pathological complete response (pCR) rates among patients with triple-negative breast cancer (TNBC), regardless of gBRCA status^31^. Furthermore, a meta-analysis of neoadjuvant regimens in patients with gBRCAm TNBC reported improved pCR rates when platin derivatives were combined with anthracyclines and taxanes, although it was unclear if this combination offered a clinically meaningful benefit over standard chemotherapy alone^32^. However, GeparSixto and INFORM did not show a benefit to adding carboplatin or cisplatin, respectively, to neoadjuvant chemotherapy for patients with gBRCAm early breast cancer^26,33^. Exploratory translational analyses of BrighTNess sought to elucidate the differences in benefit observed for patients with breast cancer and gBRCAm^34^. Higher PAM50 proliferation score, CIN70 score, and GeparSixto immune signature were associated with higher odds of pCR for both patients with and without gBRCAm, and thus have been proposed as potentially useful biomarkers for determining addition of carboplatin to neoadjuvant chemotherapy^34^, but have yet to be validated for clinical practice.

#### PARP inhibition

PARP inhibitors block the enzyme that has a vital role in repairing DNA single-strand breaks. They exploit the double-strand break repair deficiency of BRCAm cells, which accumulate unrepaired, toxic DNA double-strand breaks, thus resulting in tumor cell death (i.e., synthetic lethality). Olaparib is licensed for the adjuvant treatment of gBRCAm, HER2-negative high-risk early breast cancer, and for gBRCAm (tumor BRCAm in Japan), HER2-negative locally advanced (EU) or metastatic (EU and US) breast cancer. Talazoparib is approved for the treatment of gBRCAm, HER2-negative locally advanced or metastatic breast cancer in the US, Europe, and several other countries worldwide.

For advanced gBRCAm HER2-negative breast cancer, PARP inhibitors were approved based on the results of the OlympiAD (olaparib) and EMBRACA (talazoparib) clinical trials^5,6,35,36^. In OlympiAD, olaparib had significantly improved median progression-free survival (PFS) versus standard chemotherapy treatment of physician’s choice (7.0 months vs 4.2 months; HR 0.58 [95% CI 0.43–0.80]; *P* < 0.001) in patients with gBRCAm HER2-negative metastatic breast cancer^5^. Median OS was 19.3 months for olaparib and 17.1 months for standard chemotherapy (HR 0.89 [95% CI 0.67–1.18])^35^. In subanalyses, a potential OS benefit with first-line olaparib versus chemotherapy was observed (median 22.6 vs 14.7 months; HR 0.55 [95% CI 0.33–0.95]), with 3-year survival at 40.8% with olaparib and 12.8% with treatment of physician’s choice, which, notably, did not include a platinum regimen^5,35^. In EMBRACA, talazoparib significantly improved median PFS versus standard chemotherapy (8.6 vs 5.6 months; HR 0.54 [95% CI 0.41–0.71]; *P* < 0.001) in patients with gBRCAm advanced breast cancer^6^, with no observed improvements in OS^37^.

For early breast cancer, olaparib was approved based on the results of the phase 3 OlympiA study in patients with high-risk early gBRCAm HER2-negative breast cancer who had completed local treatment and neoadjuvant or adjuvant chemotherapy^7,38^. In the second prespecified analysis of OlympiA, adjuvant olaparib was associated with significantly improved OS versus placebo, with a 32% reduced risk of death (HR 0.68; 98.5% CI 0.47–0.97; *P* = 0.009)^7^. Significantly improved invasive disease-free survival (IDFS; HR 0.63; 95% CI 0.50–0.78) was also shown, consistent with the significantly improved IDFS reported at the first prespecified analysis (HR 0.58; 99.5% CI 0.41–0.82; *P* = 0.001)^7^.

These positive results in the adjuvant setting raised the question of whether PARP inhibitors may also have a place for neoadjuvant treatment of HER2-negative early breast cancer; however, trials have reported mixed results. In the BrighTNess trial, described above, addition of veliparib did not add benefit over neoadjuvant carboplatin/paclitaxel alone^31^. The phase 2 GeparOLA study comparing neoadjuvant paclitaxel plus olaparib to paclitaxel/carboplatinum in patients with HER2-negative breast cancer and homologous recombinant deficiency did not meet its primary endpoint (exclusion of a pCR rate of ≤55%)^39^, but did report a numerically improved pCR rate with paclitaxel/olaparib followed by epirubicin/cyclophosphamide (55.1%) versus paclitaxel/carboplatinum (48.6%) followed by epirubicin/cyclophosphamide, and a more favorable tolerability profile for paclitaxel/olaparib^39^. In the single-arm neoTALA trial, patients with gBRCAm, early-stage TNBC were treated with talazoparib followed by definitive surgery^40^. Although neoadjuvant talazoparib was active, the pCR rates did not meet the prespecified threshold of efficacy^40^. Other neoadjuvant trials are ongoing to enhance our understanding of the potential use of PARP inhibitors in early breast cancer. Of potential interest is the opportunity to evaluate alternative PARP inhibitor combinations (e.g., with immunotherapy), and tailor therapy according to the patient. For example, in the ongoing OlympiaN trial (NCT05498155) patients with deleterious/suspected deleterious BRCAm and operable, early-stage, HER2-negative, ER-negative/ER-low breast cancer are assigned olaparib (lower-risk cohort) or olaparib plus durvalumab (higher-risk cohort), and assessed for pCR^41^.

PARP inhibitors are an important treatment strategy for gBRCAm breast cancer and rely on timely access to genetic testing to guide the most appropriate treatment selection, particularly in the early breast cancer setting.

#### Cyclin-dependent kinase 4/6 (CDK4/6) inhibitors

A CDK4/6 inhibitor in combination with endocrine therapy is a recommended option for first-line treatment for certain patients with hormone receptor-positive/HER2-negative advanced or metastatic breast cancer^15,42^. Use of CDK4/6 inhibitors has also extended into earlier lines of treatment, with abemaciclib plus endocrine therapy a treatment option in the adjuvant setting for patients with hormone receptor-positive/HER2-negative, high-risk breast cancer^15^, and positive results having been reported for ribociclib (NATALEE)^43^. While the optimal sequence is not known, recent guideline updates note that when patients are eligible for both adjuvant olaparib and abemaciclib then olaparib should be given first^30,44^. Real-world evidence has suggested that patients with hormone receptor-positive advanced breast cancer and gBRCAm may have inferior outcomes with CDK4/6 inhibition or endocrine therapy versus those without gBRCAm^45–49^. This emerging finding highlights the potential importance of early detection of gBRCAm in patients with hormone receptor-positive breast cancer ahead of treatment selection, especially in light of recent CDK4/6 inhibitor approval in the early breast cancer setting.

#### Immunotherapy

There is limited evidence on the effectiveness of immunotherapy in patients with gBRCAm breast cancer. A recent substudy from the phase 3 IMpassion130 trial of the anti-programmed death-ligand 1 (PD-L1) antibody atezolizumab showed that, in combination with nab-paclitaxel, patients with PD-L1-positive advanced TNBC had an OS and PFS benefit regardless of *BRCA1/2* mutation status (germline or somatic)^50^. The efficacy of neoadjuvant PARP inhibition in combination with immunotherapy is under investigation; for example, olaparib in combination with durvalumab is being investigated in the aforementioned OlympiaN study^41^.

### Screening and counseling for family members

The burden of gBRCAm in breast cancer extends beyond the affected individual, with other family members facing decisions regarding gBRCAm testing, as well as considerations of family planning. In case of a familial association, genetic testing is recommended by the NCCN Clinical Practice Guidelines in Oncology (NCCN Guidelines^®^) for unaffected family members^21^. If a pre-symptomatic individual is identified as a carrier of gBRCAm, intensified surveillance for breast cancer is recommended, which differs per guideline but may include regular magnetic resonance imaging (MRI), ultrasound, mammography, and/or clinical breast exam, with guidance provided based on age^11,21^. For patients harboring gBRCAm with a diagnosis of breast cancer who have not undergone bilateral mastectomy, National Comprehensive Cancer Network^®^ (NCCN^®^) recommends that breast MRI and mammography should continue as recommended, based on age^21^.

For individuals undergoing pre-symptomatic testing (known gBRCAm in a family member), it is recommended that pre-test counseling topics include options for screening and early detection, the benefits and disadvantages of risk-reducing surgery (including the extent of cancer risk reduction, risks associated with surgery, management of menopausal symptoms with RRSO, psychosocial and quality-of-life impacts, and life expectancy), the benefits and limitations of reconstructive surgery and reproductive options, and the psychological implications of pre-symptomatic diagnosis^11,21^. Consideration is required with regard to reproductive concerns and the psychosocial impact of undergoing RRSO in gBRCAm carriers^21^.

## gBRCAm counseling and testing in clinical practice

### Implementation of guideline recommendations for gBRCAm counseling and testing

**Fig. 2:**
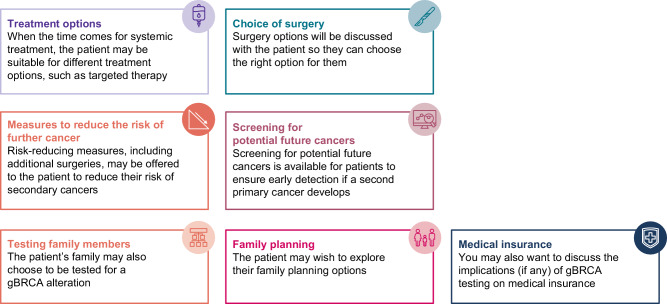
gBRCAm counseling and testing in clinical practice.

Practice guidelines for genetic counseling and gBRCAm testing are predominantly based on personal and family history of breast, ovarian, pancreatic, and/or prostate cancer; young age at diagnosis; male breast cancer; and multiple tumors (breast and ovarian) in the same patient^21^. More than 32 guidelines for gBRCAm testing relevant to breast cancer exist worldwide^11,21,51,52^, and the recommendations are often inconsistent. Many guidelines do not include recommendations for genetic counseling, or only provide counseling recommendations for patients who have been identified as carriers of gBRCAm^51^. Some guidelines recommend gBRCAm testing after genetic counseling and personalized risk assessment, and/or if the result is likely to influence the individual’s choice of primary treatment^51^. Some guidelines recommend testing based upon percentage risk of harboring a BRCA mutation, but there is a lack of consensus on the threshold used to determine whether an individual is eligible for genetics clinic referral/testing (10% vs 5%)^21,53^, and some guidelines propose testing all patients under certain circumstances (e.g., with ER-positive advanced breast cancer and resistance to endocrine therapy), considering that PARP inhibitors have a greater risk-benefit ratio than chemotherapy^54^. There are limited treatment recommendations and algorithms for women with gBRCAm-associated advanced breast cancer^51^. Greater consensus and cohesion of guidelines would be useful for patients and the medical community covering the topics highlighted in Fig. 2.

### Disparities in gBRCAm testing in clinical practice

There has been a systemic underuse of gBRCAm testing over the past two decades, which has led to inappropriate and inconsistent testing and, consequently, missed opportunities for cancer prevention and management^55^. Historically, NCCN criteria have been seen to be the least restrictive of the models, identifying a larger percentage of carriers compared with other models. However, the complex nature of the NCCN criteria render them difficult to implement in real-world clinical practice^51^, with low adherence rates reported^56^. Expansion of NCCN criteria to include all women diagnosed at ≤65 years of age was shown to improve sensitivity of the selection criteria, without requiring testing of all women with breast cancer^57^.

**Fig. 3:**
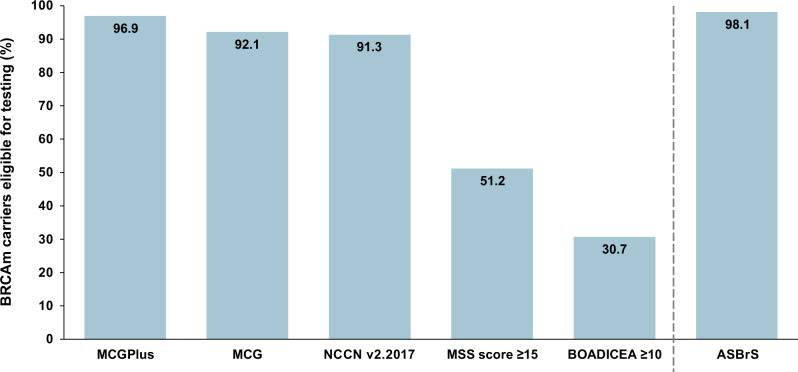
Eligibility for BRCAm testing using different testing criteria. The graph shows estimates of patient eligibility for BRCA testing among BRCAm carriers. Data to the left of the dashed line is reproduced from a report in 2019 by the MCG group assessing rates of testing eligibility by different criteria^55^, while the bar to the right of the dashed line illustrates the result of an analysis by ASBrS published in 2020, examining the effect of including all individuals meeting NCCN criteria v1.2020 plus those diagnosed with breast cancer at ≤65 years^57^. ASBrS, American Society of Breast Surgeons; BOADICEA, Breast and Ovarian Analysis of Disease Incidence and Cancer Estimation Algorithm (≥10 refers to a 10% or greater probability that a BRCA1 or BRCA2 mutation is present); MCG, Mainstreaming Cancer Genetics; MSS, Manchester Scoring System; NCCN, National Comprehensive Cancer Network^®^ (NCCN^®^).

Although recent data from some centers and countries suggest widespread routine gBRCAm testing^58^, a number of reports highlight the need for broader eligibility criteria for gBRCAm testing to ensure that more individuals can have access^55,57,59,60^. Notably, patient eligibility for gBRCAm testing has been shown to vary depending on different testing criteria and recommendations, ranging from over 98% using recent guidelines published by the American Society of Breast Surgeons (ASBrS) to only around 30% eligibility using the Breast and Ovarian Analysis of Disease Incidence and Cancer Estimation Algorithm (BOADICEA) criteria^55,57^ (Fig. 3). Simplified, cost-effective eligibility criteria for gBRCAm testing, based on individual rather than family history criteria, have been proposed by the Mainstreaming Cancer Genetics (MCG) group. The five eligibility criteria include: (1) ovarian cancer diagnosis, (2) breast cancer diagnosed ≤45 years of age, (3) two primary breast cancers, both diagnosed ≤60 years of age, (4) TNBC diagnosis, and (5) male breast cancer diagnosis^55^. In an analysis of different guidelines, using these criteria would have tested 92% of people and detected 100% of gBRCAm carriers^55^. An additional sixth criteria (breast cancer, plus a parent, sibling, or child meeting any of the other criteria) further improved the eligibility rate to 97% (MCGplus)^55^, while expansion of NCCN criteria (v1.2020) to include individuals diagnosed at ≤65 years of age, as recommended by ASBrS, increased testing eligibility to include over 98% of BRCAm carriers^57^ (Fig. 3). Both the MCG and MCGplus criteria were deemed cost-effective, with cost-effective ratios of $1330 and $1225 per discounted quality-adjusted life year for the MCG and MCGplus criteria, respectively^55^. Additional studies have sought to investigate the cost-effectiveness of BRCA testing in all patients with breast cancer, with several studies conducted in countries such as Australia, China, Norway, Malaysia, the UK, and the US finding this to be a potentially cost-effective strategy^61–65^.

### Traditional genetic counseling and testing pathway

**Fig. 4:**
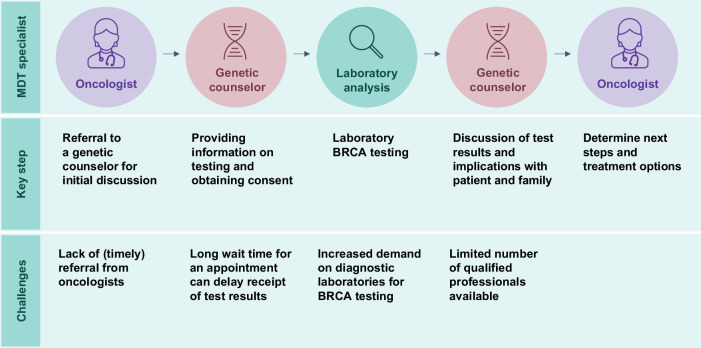
Key steps and challenge points in traditional gBRCAm testing pathways. MDT, multidisciplinary team.

The traditional pathway of genetic testing involves individualized patient referral to the genetics department for the management of pre-test genetic counseling, consenting, sample acquisition, and return of results (Fig. 4). Pre-test counseling, and the process of informed consent, focuses on giving patients sufficient information about the test, its limitations, and the consequences (including psychological) of a positive result, to enable an informed decision as to whether or not to proceed^9^. Patients who test positive for gBRCAm receive post-test support from a geneticist/genetic counselor/expert^9,66^.

Genetic professionals offering counseling include both medical genetic physicians (professionals with advanced training, such as an MD with a specialization in genetic medicine) and genetic counselors (professionals with a specialized Masters degree in genetic counseling)^67,68^. Genetic counseling by a trained genetics clinician has been shown to improve patient knowledge, understanding, and satisfaction among patients^69^, and is recommended in multiple guidelines^11,21^. While advantages of this type of care are clear, disadvantages include that it can be time-consuming, and a limited number of professionals are appropriately trained. When rapid access to test results is required to inform treatment decisions in a time-sensitive manner, especially for those undergoing upfront surgery, it may not be possible to maintain this workflow, and innovative alternatives may be required^70^.

Although genetic counseling is recommended, a dearth of adequately trained professionals in this field may limit access^71^, with some countries imposing legal requirements for practicing genetic counseling^72^. Where possible, non-geneticist physicians might feel the need to counsel and test patients themselves without support, despite increasing demands on their time and shorter appointment times^69,71^. Across Canada and the US, there are approximately 1.5 genetic counselors per 100,000 individuals, and it is estimated that double the workforce will be needed to meet future demands^73^. There has been an increase in genetic counselors reporting the use of multiple types of delivery models, including telephone and telegenetics, with an aim of improving access and efficiency of genetic counseling; however, barriers remain that can hinder implementation of these models^74^. In a large, US population-based study, only 62% of high-risk patients with newly diagnosed breast cancer who were tested had a genetic counselor session^75^. Furthermore, 66% of all patients, and 81% of high-risk patients, wanted testing but only 29 and 53% received it, respectively^75^. The most common reason for high-risk patients not being tested was “my doctor didn’t recommend it”^75^. Wait times to see genetic specialists can also be substantial. In the UK, the Nottingham University Hospitals National Health Service (NHS) Trust reports wait times of 12–14 weeks for an initial appointment and 4–6 months to receive results^76^. This highlights the need for alternative models of counseling and consenting of patients to ensure all eligible patients receive testing in a timely manner.

Systemic and societal barriers can impede equitable access to the benefits of genetic testing. Suboptimal testing rates among individuals of low socioeconomic status have been largely attributed to perceived/actual financial costs of genetic testing, with patients and healthcare providers often unclear as to whether genetic counseling services and follow-up care are covered by health insurance^10,77^. Strategies to improve testing rates in this patient demographic include the integration of genetic counselors into primary care settings to reduce travel time and costs to the patient^78^, and lobbying for expanded health insurance coverage for genetic counseling and testing services^79^.

Reports from US ovarian and breast cancer centers have consistently found racial/ethnic disparity in access to genetic testing, with referral rates being higher for non-Hispanic White women than for women of other races^80–82^. Lower awareness of the genetic basis of risk, incomplete family history, and mistrust of medical confidentiality may contribute to racial/ethnic disparities in referrals for genetic testing^79,83^. In addition, the detection of pathogenic variants may be decreased, and variants of uncertain significance increased, in non-White individuals^84–86^, as genomic reference databases provide poor genetic representation of non-White populations^87,88^. Whilst initiatives have been established to address gaps in the diversity of genomic data^89^, additional strategies are required to increase genetic testing rates among non-White populations. These include the development of culturally and linguistically tailored educational material, extended appointment availability, increased training of primary care-based specialists to mitigate unconscious or implicit biases, and a drive to recruit and train more healthcare providers from minority backgrounds^79,80,90^.

### Mainstream genetic counseling and testing pathways

**Fig. 5:**
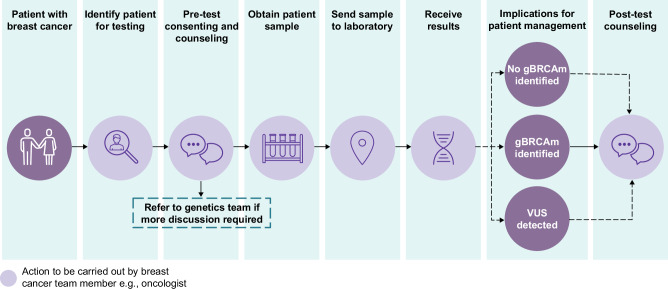
Example gBRCAm testing pathway to illustrate the mainstream genetic testing pathway. VUS, variant of uncertain significance.

In mainstream genetic testing pathways, medical oncology teams are responsible for pre-test genetic counseling, obtaining consent, scheduling the genetic test, and using the results to guide treatment decisions (Fig. 5)^55,91,92^. Implementation of mainstream models has enabled more efficient testing of patients with ovarian cancer and has significantly increased the proportion of patients being offered genetic testing^93–95^.

Mainstream genetic testing models for patients with breast cancer have also proven effective, with high pathogenic variant detection rates and a reduced burden on genetic services observed^55,66,76^. A Canadian study reported a significant decrease in wait time from referral to the return of genetic test results using an oncology clinic-based model compared with a traditional model in patients with breast or ovarian cancer (403 vs 191 days; *P* < 0.001)^96^. Other studies support that oncologist-led mainstreaming results in increased testing uptake and shorter test-turnaround times^97,98^. A systematic review of 15 studies in patients with breast, ovarian, endometrial, or prostate cancer showed that turnaround times with the mainstream approach are lower than those with the traditional pathway, with results typically obtained 3–6 weeks after discussing and ordering the genetic test^92^. Another study in patients with breast cancer measured an 85% reduction in time to test result using the mainstream model compared with the traditional model (4 vs 25 weeks)^55^. A mainstreaming program in Australia had successful uptake with a notable gBRCAm detection rate and a reduced burden on the center, enabling reallocation of resources to streamline the genetic testing process^66^. Mainstream models also reduce genetic consultation requirements versus traditional models^55,66^.

#### Perspectives of the multidisciplinary team

Oncogenetic partnership models, in which clinical teams order genetic testing in collaboration with geneticists and implement counseling at both an individual and group level, have been shown to improve access to counseling and reduce turnaround times for genetic testing^99^. However, the feasibility of implementing new testing strategies may vary by region.

As part of the MCG program in breast cancer in the UK and Malaysia, 100% of team members (12 oncologists, 8 surgeons, and 3 nurse specialists) reported feeling confident to approve patients for genetic testing, and believed that the process worked well^55^. Similar experiences have been reported among ovarian cancer teams^9,91^. However, another study surveying oncologists, clinical geneticists, and surgeons found that while oncologists and clinical geneticists were mainly positive about the introduction of mainstream approaches, surgeons were not keen to implement mainstream services in their breast clinic, feeling that they did not have the expertise, time, or capacity to undertake the extra responsibility, and that genetic testing did not have much relevance for their treatment decision-making^100^.

Nurses play an integral role within the oncology team, with clinical nurse specialists often being the key point of contact for patients throughout the cancer pathway and thus ideally placed to deliver information on gBRCAm testing. A single-center UK study assessing the use of clinical nurse specialists in consenting patients with ovarian cancer for gBRCAm testing showed that there was no difference in patient-reported satisfaction compared with oncologist-led consenting, and nurses felt confident in counseling, consenting, and returning results^9^. A specialist, nurse-led breast cancer MCG service established at the Nottingham Breast Institute, UK, has reduced wait time from the date of testing to the date of results to 36 days compared with an historical wait time of 4–6 months, while also delivering continuity of care for patients, releasing oncologists’ time, and allowing oncologists and patients to consider treatment options at an earlier time point^76^. The potential for nurses to play a role in decision coaching in healthy individuals who are carriers of gBRCAm is being explored, with preliminary results suggesting the approach is feasible^101,102^. We provide an educational guide for nurses to outline the role that nurses can play in the gBRCA testing pathway and support conversations around nurse-obtained consent (supplementary information**:** *Nurse consenting guide for germline BRCA testing*).

#### The patients’ perspective

Genetic testing in mainstream oncology units is widely accepted by patients^55,66,91,103–106^. In the MCG breast cancer program, 96% of patients were happy that genetic testing was performed by their cancer team^55^. Some patients reported a preference for their medical oncologist or their oncology nurse to deliver pre-test counseling, because medical oncologists could use the information gained through genetic testing for treatment decisions, and because nurses are more familiar with, and better understand, the individual patient experience^105^.

#### Educational needs for non-genetic specialists

Ensuring appropriate training on an ongoing basis for those involved in consenting and arranging genetic testing is paramount to the success of mainstream gBRCAm testing^66^. An early study evaluating patient experiences of gBRCAm testing in the US (all tumor types) found that the quality of information given to patients by non-certified genetic healthcare professionals (HCPs) was not as consistent as that given by certified genetic HCPs, with far fewer patients in mainstream testing versus traditional counseling recalling having had a pre-test discussion, and what that included^107^. The types of training required by non-genetic specialists include generic consent training, plus specific genetics training, which involves learning how to identify eligible patients for gBRCAm testing, the relevance of gBRCAm testing, the significance for patients with a positive or negative result, the significance of a gBRCAm variant requiring evaluation, and implications of a positive test for family members^9^. Workshops designed to improve HCP knowledge and self-confidence have been shown to significantly enhance ability to overcome communication difficulties in relation to genetic testing and counseling^108^. We provide educational guides to support healthcare providers in their understanding of the gBRCA testing and consenting pathway (supplementary information: *HCP guide to genetic counseling: Understanding germline BRCA testing and its clinical implications in breast cancer* and *Germline BRCA testing pathway infographic*), as well as useful language to help explain the process to patients (supplementary information: *Patient-HCP flipbook: What you need to know about BRCA testing*).

### Use of digital tools

Digital tools are being increasingly used across the genetic testing pathway for clinical assessment, family history taking, education, post-test counseling, and follow-up, and include web-based tools, mobile applications, chatbots, videos, and games^73,109,110^. They have been shown to improve access to genetic testing (particularly for patients in under-served areas), reduce waiting times, enhance continuity of care, increase patient engagement, and free up time for other patient-centered consultations^73,110^. Digital tools are associated with positive patient outcomes, including increased knowledge and reduced decision conflict, and achieve similar patient outcomes to in-person consultations^109^.

There are no digital tools that offer a comprehensive solution across the entire genetic counseling and testing pathway, with most tools developed for use in the pre-test counseling phase only^109^. The Genetics Navigator is currently in development and aims to supplement in-person consultations and support the full genetic testing pathway, including pre-test counseling, education, decision support, laboratory reporting, personalized return of results, and post-test counseling^110^. A digital pathway has also been integrated into the UK NHS clinical, laboratory, and informatics systems for delivery of gBRCAm testing to cancer patients and has been piloted as part of the BRCA-DIRECT study^111^. Results demonstrated that uptake of genetic testing using the digital pathway was non-inferior to those receiving pre-test information via telephone, with similarly good patient satisfaction and knowledge and low anxiety scores^111^.

## The future of genetic testing in breast cancer

### Germline versus somatic mutation testing

Genetic testing of tumor tissue has the potential to identify both germline and somatic pathogenic (or likely pathogenic) variants, and thus identify more people who might benefit from targeted therapies. Indeed, several studies have demonstrated clinical benefit with PARP inhibitor treatment for somatic BRCAm (sBRCAm) breast cancers^112–114^. High concordance between germline and tumor BRCAm testing in breast and ovarian cancer has been observed^115–120^; however, while sBRCAm and gBRCAm can be mutually exclusive in breast cancer, and not all mutation types can be detected by current clinical testing methods, it is possible that patients with metastatic breast cancer could benefit more from tumor testing than germline testing, as other abnormalities and targets could be identified^121–123^. For example, approvals of alpelisib plus fulvestrant for the treatment of *PIK3CA*m advanced or metastatic breast cancer^124^, capivasertib plus fulvestrant for advanced or metastatic breast cancer with *PIK3CA*m, *AKT1*m, or *PTEN*m^125^, and pembrolizumab for the treatment of unresectable or metastatic solid tumors of any type with high tumor mutational burden^126^ may have led to an increase in patient referrals for tumor testing using a gene panel assay. An increasing number of patients with BRCAm breast cancer could, therefore, receive an incidental positive result for BRCAm and be subsequently offered a gBRCA test to confirm the somatic or germline status, in accordance with NCCN Guidelines^®^^21^.

Parallel testing of normal and tumor material offers an alternative approach that allows direct differentiation of somatic versus germline pathogenic (or likely pathogenic) variants, leading to timely treatment selection and genetic counseling that may otherwise be delayed with germline- or tumor-only testing^127^. Somatic testing alone would not distinguish between germline and somatic pathogenic (or likely pathogenic) variants, and thus may not be useful for determining future surveillance/surgery options for the patient, and may not benefit family members^128^. Therefore, an increasing number of centers are moving toward parallel testing for patients with a breast cancer diagnosis^127^. Analysis of circulating tumor DNA has the potential to identify both somatic and germline variants, and may offer a non-invasive alternative to tissue testing^129^.

### Genetic testing beyond BRCA

Panel testing allows for the screening of multiple genes beyond *BRCA1* and *BRCA2* that may be associated with tumor development and/or treatment response^130^. For example, several other factors in the homologous recombination pathway have emerged as clinically relevant in surgical and treatment decision-making^131,132^. Pathogenic variants in breast cancer susceptibility genes beyond *BRCA1* and *BRCA2* are increasingly being considered in clinical trials with targeted therapies^113,133,134^ and further recommendations for risk reduction, screening, and treatment strategies for carriers of these variants are being incorporated into clinical practice guideline updates and risk assessment tools^11,21,52^. For example, current NCCN Guidelines recommend discussion of risk-reducing mastectomy with patients found to harbor pathogenic or likely pathogenic variants in *CDH1, PALB2, PTEN, STK11*, or *TP53*, and consideration of RRSO at 45–50 years of age in patients with pathogenic or likely pathogenic variants in *PALB2*, *RAD51C*, or *RAD51D*^21^. The web-based CanRisk tool, which integrates the presence of pathogenic variants in eight breast cancer susceptibility genes with several other risk factors to estimate the personal risk of breast cancer, is currently endorsed by multiple clinical guidelines^135,136^.

To summarize, advancements in patient information and care, in particular the introduction of PARP inhibitors for the treatment of breast and other cancers, have resulted in a substantial increase in demand for genetic testing. This demand is supported by the evidence that gBRCA testing in breast cancer management is a cost-effective strategy. However, without a substantial increase in personnel, traditional, genetics-led models of counseling and consenting are unable to meet the growing demand. A case can be made to increase the number of genetically trained HCPs but, even if possible, there will be a certain time lag before they are available. Mainstreaming models and the use of digital tools have demonstrated potential in providing efficient, patient-centered care that can meet the increasing needs of patients. In the future, we may see a move toward more widespread and comprehensive testing for germline and tumor alterations, raising further challenges as to how this can be effectively incorporated into comprehensive cancer care.

## Supplementary information

Supplementary information

## References

[1] Wooster, R. et al. Identification of the breast cancer susceptibility gene BRCA2. *Nature* **378**, 789–792 (1995). 8524414 10.1038/378789a0

[2] Albertsen, H. et al. Genetic mapping of the BRCA1 region on chromosome 17q21. *Am. J. Hum. Genet.* **54**, 516–525 (1994). 8116621 PMC1918118

[3] Roy, R., Chun, J. & Powell, S. N. BRCA1 and BRCA2: different roles in a common pathway of genome protection. *Nat. Rev. Cancer* **12**, 68–78 (2011). 22193408 10.1038/nrc3181 PMC4972490

[4] Wang, S. S. Y., Jie, Y. E., Cheng, S. W., Ling, G. L. & Ming, H. V. Y. PARP inhibitors in breast and ovarian cancer. *Cancers* **15**, 2357 (2023). 10.3390/cancers15082357 PMC10137187 37190285

[5] Robson, M. et al. Olaparib for metastatic breast cancer in patients with a germline BRCA mutation. *N. Engl. J. Med.* **377**, 523–533 (2017). 28578601 10.1056/NEJMoa1706450

[6] Litton, J. K. et al. Talazoparib in patients with advanced breast cancer and a germline BRCA mutation. *N. Engl. J. Med.* **379**, 753–763 (2018). 30110579 10.1056/NEJMoa1802905 PMC10600918

[7] Geyer, C. E. Jr et al. Overall survival in the OlympiA phase III trial of adjuvant olaparib in patients with germline pathogenic variants in BRCA1/2 and high risk, early breast cancer. *Ann. Oncol.* **33**, 1250–1268 (2022). 36228963 10.1016/j.annonc.2022.09.159 PMC10207856

[8] Scheinberg, T. et al. Mainstream consent programs for genetic counseling in cancer patients: a systematic review. *Asia Pac. J. Clin. Oncol.* **17**, 163–177 (2021). 32309911 10.1111/ajco.13334

[9] Percival, N. et al. The integration of BRCA testing into oncology clinics. *Br. J. Nurs.* **25**, 690–694 (2016). 27345073 10.12968/bjon.2016.25.12.690 PMC5993185

[10] Dusic, E. J. et al. Barriers, interventions, and recommendations: improving the genetic testing landscape. *Front. Digit. Health* **4**, 961128 (2022). 36386046 10.3389/fdgth.2022.961128 PMC9665160

[11] Sessa, C. et al. Risk reduction and screening of cancer in hereditary breast-ovarian cancer syndromes: ESMO Clinical Practice Guideline. *Ann. Oncol.* **34**, 33–47 (2023). 36307055 10.1016/j.annonc.2022.10.004

[12] Ford, D. et al. Genetic heterogeneity and penetrance analysis of the BRCA1 and BRCA2 genes in breast cancer families. The Breast Cancer Linkage Consortium. *Am. J. Hum. Genet.* **62**, 676–689 (1998). 9497246 10.1086/301749 PMC1376944

[13] Kuchenbaecker, K. B. et al. Risks of breast, ovarian, and contralateral breast cancer for BRCA1 and BRCA2 mutation carriers. *JAMA* **317**, 2402–2416 (2017). 28632866 10.1001/jama.2017.7112

[14] Lambertini, M. et al. Clinical behavior and outcomes of breast cancer in young women with germline BRCA pathogenic variants. *NPJ Breast Cancer* **7**, 16 (2021). 33579978 10.1038/s41523-021-00224-w PMC7880991

[15] Referenced with permission from the NCCN Clinical Practice Guidelines in Oncology (NCCN Guidelines^®^) for Breast Cancer Version 2.2024. © National Comprehensive Cancer Network, Inc. 2024. All rights reserved. Accessed May 17, 2024. To view the most recent and complete version of the guideline, go online to NCCN.org. NCCN makes no warranties of any kind whatsoever regarding their content, use or application and disclaims any responsibility for their application or use in any way.

[16] Schwartz, G. F. et al. Consensus conference on breast conservation, Milan, Italy, April 28-May 1, 2005. *Breast J.* **12**, 398–407 (2006). 16848863 10.1111/j.1075-122X.2006.00292.x

[17] Tung, N. M. et al. Management of hereditary breast cancer: American Society of Clinical Oncology, American Society for Radiation Oncology, and Society of Surgical Oncology Guideline. *J. Clin. Oncol.* **38**, 2080–2106 (2020). 32243226 10.1200/JCO.20.00299

[18] Scheepens, J. C. C., Veer, L. V., Esserman, L., Belkora, J. & Mukhtar, R. A. Contralateral prophylactic mastectomy: a narrative review of the evidence and acceptability. *Breast* **56**, 61–69 (2021). 33621798 10.1016/j.breast.2021.02.003 PMC7907889

[19] Jia, Z. et al. Contralateral risk-reducing local therapy in breast cancer patients with BRCA1/2 mutations: systemic review and meta-analysis. *Cancer Cell Int.* **21**, 512 (2021). 34563200 10.1186/s12935-021-02194-2 PMC8466340

[20] van Sprundel, T. C. et al. Risk reduction of contralateral breast cancer and survival after contralateral prophylactic mastectomy in BRCA1 or BRCA2 mutation carriers. *Br. J. Cancer* **93**, 287–292 (2005). 16052221 10.1038/sj.bjc.6602703 PMC2361560

[21] Referenced with permission from the NCCN Clinical Practice Guidelines in Oncology (NCCN Guidelines®) for Genetic/Familial High-Risk Assessment: Breast, Ovarian, and Pancreatic V.3.2024. © National Comprehensive Cancer Network, Inc. 2024. All rights reserved. Accessed May 17, 2024. To view the most recent and complete version of the guideline, go online to NCCN.org. NCCN makes no warranties of any kind whatsoever regarding their content, use or application and disclaims any responsibility for their application or use in any way.

[22] Wang, Y., Song, Z., Zhang, S., Wang, X. & Li, P. Risk-reducing salpingo-oophorectomy and breast cancer risk in BRCA1 or BRCA2 mutation carriers: a systematic review and meta-analysis. *Eur. J. Surg. Oncol.* **48**, 1209–1216 (2022). 35216860 10.1016/j.ejso.2022.02.019

[23] Metcalfe, K. et al. Effect of oophorectomy on survival after breast cancer in BRCA1 and BRCA2 mutation carriers. *JAMA Oncol.* **1**, 306–313 (2015). 26181175 10.1001/jamaoncol.2015.0658

[24] Terry, M. B. et al. Risk-reducing oophorectomy and breast cancer risk across the spectrum of familial risk. *J. Natl Cancer Inst.* **111**, 331–334 (2019). 30496449 10.1093/jnci/djy182 PMC6410936

[25] Heemskerk-Gerritsen, B. A. et al. Breast cancer risk after salpingo-oophorectomy in healthy BRCA1/2 mutation carriers: revisiting the evidence for risk reduction. *J. Natl Cancer Inst.* **107**, djv033 (2015). 10.1093/jnci/djv033 25788320

[26] Hahnen, E. et al. Germline mutation status, pathological complete response, and disease-free survival in triple-negative breast cancer: secondary analysis of the GeparSixto randomized clinical trial. *JAMA Oncol.* **3**, 1378–1385 (2017). 28715532 10.1001/jamaoncol.2017.1007 PMC5710508

[27] Tutt, A. et al. Carboplatin in BRCA1/2-mutated and triple-negative breast cancer BRCAness subgroups: the TNT Trial. *Nat. Med.* **24**, 628–637 (2018). 29713086 10.1038/s41591-018-0009-7 PMC6372067

[28] Pohl-Rescigno, E. et al. Association of germline variant status with therapy response in high-risk early-stage breast cancer: a secondary analysis of the GeparOcto randomized clinical trial. *JAMA Oncol.* **6**, 744–748 (2020). 32163106 10.1001/jamaoncol.2020.0007 PMC7068666

[29] Fasching, P. A. et al. BRCA1/2 mutations and bevacizumab in the neoadjuvant treatment of breast cancer: response and prognosis results in patients with triple-negative breast cancer from the GeparQuinto study. *J. Clin. Oncol.* **36**, 2281–2287 (2018). 29791287 10.1200/JCO.2017.77.2285 PMC6067803

[30] Loibl, S. et al. Early breast cancer: ESMO Clinical Practice Guideline for diagnosis, treatment and follow-up. *Ann. Oncol.* **35**, 159–182 (2024). 38101773 10.1016/j.annonc.2023.11.016

[31] Loibl, S. et al. Addition of the PARP inhibitor veliparib plus carboplatin or carboplatin alone to standard neoadjuvant chemotherapy in triple-negative breast cancer (BrighTNess): a randomised, phase 3 trial. *Lancet Oncol.* **19**, 497–509 (2018). 29501363 10.1016/S1470-2045(18)30111-6

[32] Caramelo, O. et al. Efficacy of different neoadjuvant treatment regimens in BRCA-mutated triple negative breast cancer: a systematic review and meta-analysis. *Hered. Cancer Clin. Pract.* **20**, 34 (2022). 36085046 10.1186/s13053-022-00242-0 PMC9463858

[33] Tung, N. et al. TBCRC 031: randomized phase II study of neoadjuvant cisplatin versus doxorubicin-cyclophosphamide in germline BRCA carriers with HER2-negative breast cancer (the INFORM trial). *J. Clin. Oncol.* **38**, 1539–1548 (2020). 32097092 10.1200/JCO.19.03292 PMC8462533

[34] Metzger-Filho, O. et al. Matched cohort study of germline BRCA mutation carriers with triple negative breast cancer in brightness. *NPJ Breast Cancer* **7**, 142 (2021). 34764307 10.1038/s41523-021-00349-y PMC8586340

[35] Robson, M. E. et al. OlympiAD extended follow-up for overall survival and safety: olaparib versus chemotherapy treatment of physician’s choice in patients with a germline BRCA mutation and HER2-negative metastatic breast cancer. *Eur. J. Cancer* **184**, 39–47 (2023). 36893711 10.1016/j.ejca.2023.01.031 PMC10585240

[36] Robson, M. E. et al. OlympiAD final overall survival and tolerability results: olaparib versus chemotherapy treatment of physician’s choice in patients with a germline BRCA mutation and HER2-negative metastatic breast cancer. *Ann. Oncol.* **30**, 558–566 (2019). 30689707 10.1093/annonc/mdz012 PMC6503629

[37] Litton, J. K. et al. Talazoparib versus chemotherapy in patients with germline BRCA1/2-mutated HER2-negative advanced breast cancer: final overall survival results from the EMBRACA trial. *Ann. Oncol.* **31**, 1526–1535 (2020). 32828825 10.1016/j.annonc.2020.08.2098 PMC10649377

[38] Tutt, A. N. J. et al. Adjuvant olaparib for patients with BRCA1- or BRCA2-mutated breast cancer. *N. Engl. J. Med.* **384**, 2394–2405 (2021). 34081848 10.1056/NEJMoa2105215 PMC9126186

[39] Fasching, P. A. et al. Neoadjuvant paclitaxel/olaparib in comparison to paclitaxel/carboplatinum in patients with HER2-negative breast cancer and homologous recombination deficiency (GeparOLA study). *Ann. Oncol.* **32**, 49–57 (2021). 33098995 10.1016/j.annonc.2020.10.471

[40] Litton, J. K. et al. Neoadjuvant talazoparib in patients with germline BRCA1/2 mutation-positive, early-stage triple-negative breast cancer: results of a phase II study. *Oncologist* **28**, 845–855 (2023). 37318349 10.1093/oncolo/oyad139 PMC10546823

[41] Balmaña, J. et al. Abstract OT2-18-02: OlympiaN: a phase 2, multicenter, open-label study to assess the efficacy and safety of neoadjuvant olaparib monotherapy and olaparib plus durvalumab in patients with BRCA mutations and early-stage HER2-negative breast cancer. *Cancer Res.* **83**, OT2-18-02–OT12-18-02 (2023). 10.1158/1538-7445.SABCS22-OT2-18-02

[42] Cardoso, F. et al. 5th ESO-ESMO international consensus guidelines for advanced breast cancer (ABC 5). *Ann. Oncol.* **31**, 1623–1649 (2020). 32979513 10.1016/j.annonc.2020.09.010 PMC7510449

[43] Slamon, D. et al. Ribociclib and endocrine therapy as adjuvant treatment in patients with HR./HER2- early breast cancer: primary results from the phase III NATALEE trial. *J. Clin. Oncol.* **41**, LBA500 (2023). 10.1200/JCO.2023.41.17_suppl.LBA500

[44] Curigliano, G. et al. Understanding breast cancer complexity to improve patient outcomes: the St Gallen International Consensus Conference for fhe Primary Therapy of Individuals with Early Breast Cancer 2023. *Ann. Oncol.* **34**, 970–986 (2023). 37683978 10.1016/j.annonc.2023.08.017

[45] Collins, J. M. et al. A real-world evidence study of CDK4/6 inhibitor treatment patterns and outcomes in metastatic breast cancer by germline BRCA mutation status. *Oncol. Ther.* **9**, 575–589 (2021). 34308518 10.1007/s40487-021-00162-4 PMC8593114

[46] Park, Y. H. et al. Longitudinal multi-omics study of palbociclib resistance in HR-positive/HER2-negative metastatic breast cancer. *Genome Med.* **15**, 55 (2023). 37475004 10.1186/s13073-023-01201-7 PMC10360358

[47] Bruno, L. et al. Cyclin-dependent kinase 4/6 inhibitor outcomes in patients with advanced breast cancer carrying germline pathogenic variants in DNA repair-related genes. *JCO Precis. Oncol.* **6**, e2100140 (2022). 35235412 10.1200/PO.21.00140

[48] Antrás, J. F. et al. Impact of pathogenic germline BRCA1/2 and PALB2 mutations and tumor aneuploidy in patients with HR+/HER2- metastatic breast cancer treated with CDK4/6 inhibitors. *J. Clin. Oncol.* **41**, 1075 (2023).

[49] Park, S. Y. et al. Prognostic role of tumor subtype and germline BRCA mutation in advanced breast cancer patients treated with palbociclib plus endocrine therapy. *Breast Cancer Res. Treat.* **196**, 121–128 (2022). 36070058 10.1007/s10549-022-06566-8

[50] Emens, L. A. et al. Atezolizumab and nab-paclitaxel in advanced triple-negative breast cancer: biomarker evaluation of the IMpassion130 study. *J. Natl Cancer Inst.* **113**, 1005–1016 (2021). 33523233 10.1093/jnci/djab004 PMC8328980

[51] Forbes, C., Fayter, D., de Kock, S. & Quek, R. G. A systematic review of international guidelines and recommendations for the genetic screening, diagnosis, genetic counseling, and treatment of BRCA-mutated breast cancer. *Cancer Manag. Res.* **11**, 2321–2337 (2019). 30962720 10.2147/CMAR.S189627 PMC6434912

[52] Bedrosian, I. et al. Germline testing in patients with breast cancer: ASCO-Society of Surgical Oncology guideline. *J. Clin. Oncol*. **42**, 584–604 (2024). 10.1200/JCO.23.02225 38175972

[53] NICE. Familial breast cancer: classification, care and managing breast cancer and related risks in people with a family history of breast cancer. (National Institute for Health and Care Excellence). Available from https://www.nice.org.uk/guidance/cg164/resources/familial-breast-cancer-classification-care-and-managing-breast-cancer-and-related-risks-in-people-with-a-family-history-of-breast-cancer-pdf-35109691767493 (Accessed August 2024), 2023. 31940157

[54] Pujol, P. et al. Clinical practice guidelines for BRCA1 and BRCA2 genetic testing. *Eur. J. Cancer* **146**, 30–47 (2021). 33578357 10.1016/j.ejca.2020.12.023

[55] Kemp, Z. et al. Evaluation of cancer-based criteria for use in mainstream BRCA1 and BRCA2 genetic testing in patients with breast cancer. *JAMA Netw. Open* **2**, e194428 (2019). 31125106 10.1001/jamanetworkopen.2019.4428 PMC6632150

[56] Alberty-Oller, J. J. et al. Adherence to NCCN Guidelines for genetic testing in breast cancer patients: who are we missing? *Ann. Surg. Oncol.* **28**, 281–286 (2021). 32918176 10.1245/s10434-020-09123-z

[57] Yadav, S. et al. Evaluation of germline genetic testing criteria in a hospital-based series of women with breast cancer. *J. Clin. Oncol.* **38**, 1409–1418 (2020). 32125938 10.1200/JCO.19.02190 PMC7193748

[58] Lux, M. P. & Fasching, P. A. Breast cancer and genetic BRCA1/2 testing in routine clinical practice: why, when and for whom? *Geburtshilf. Frauenheilkd.* **83**, 310–320 (2023). 10.1055/a-1929-2629 PMC9998182 36908286

[59] Manahan, E. R. et al. Consensus guidelines on genetic testing for hereditary breast cancer from the American Society of Breast Surgeons. *Ann. Surg. Oncol.* **26**, 3025–3031 (2019). 31342359 10.1245/s10434-019-07549-8 PMC6733830

[60] Childers, C. P., Childers, K. K., Maggard-Gibbons, M. & Macinko, J. National estimates of genetic testing in women with a history of breast or ovarian cancer. *J. Clin. Oncol.* **35**, 3800–3806 (2017). 28820644 10.1200/JCO.2017.73.6314 PMC5707208

[61] Tuffaha, H. W. et al. Cost-effectiveness analysis of germ-line BRCA testing in women with breast cancer and cascade testing in family members of mutation carriers. *Genet. Med.* **20**, 985–994 (2018). 29300376 10.1038/gim.2017.231

[62] Lim, K. K. et al. Is BRCA mutation testing cost effective for early stage breast cancer patients compared to routine clinical surveillance? The case of an upper middle-income country in Asia. *Appl. Health Econ. Health Policy* **16**, 395–406 (2018). 29572724 10.1007/s40258-018-0384-8

[63] Norum, J. et al. BRCA mutation carrier detection. A model-based cost-effectiveness analysis comparing the traditional family history approach and the testing of all patients with breast cancer. *ESMO Open* **3**, e000328 (2018). 29682331 10.1136/esmoopen-2018-000328 PMC5905828

[64] Sun, L. et al. A cost-effectiveness analysis of multigene testing for all patients with breast cancer. *JAMA Oncol.* **5**, 1718–1730 (2019). 31580391 10.1001/jamaoncol.2019.3323 PMC6777250

[65] Wu, H. L. et al. All HER2-negative breast cancer patients need gBRCA testing: cost-effectiveness and clinical benefits. *Br. J. Cancer* **128**, 638–646 (2023). 36564566 10.1038/s41416-022-02111-y PMC9938252

[66] Beard, C., Monohan, K., Cicciarelli, L. & James, P. A. Mainstream genetic testing for breast cancer patients: early experiences from the Parkville Familial Cancer Centre. *Eur. J. Hum. Genet.* **29**, 872–880 (2021). 33723355 10.1038/s41431-021-00848-3 PMC8111023

[67] Abacan, M. et al. The global state of the genetic counseling profession. *Eur. J. Hum. Genet.* **27**, 183–197 (2019). 30291341 10.1038/s41431-018-0252-x PMC6336871

[68] Maiese, D. R. et al. The 2019 medical genetics workforce: a focus on laboratory geneticists. *Genet. Med.* **25**, 100834 (2023). 36999554 10.1016/j.gim.2023.100834

[69] Armstrong, J. et al. Utilization and outcomes of BRCA genetic testing and counseling in a national commercially insured population: the ABOUT study. *JAMA Oncol.* **1**, 1251–1260 (2015). 26426480 10.1001/jamaoncol.2015.3048

[70] Torr, B. et al. A digital pathway for genetic testing in UK NHS patients with cancer: BRCA-DIRECT randomised study internal pilot. *J. Med. Genet.* **59**, 1179–1188 (2022). 35868849 10.1136/jmg-2022-108655 PMC9691828

[71] Delikurt, T., Williamson, G. R., Anastasiadou, V. & Skirton, H. A systematic review of factors that act as barriers to patient referral to genetic services. *Eur. J. Hum. Genet.* **23**, 739–745 (2015). 25205405 10.1038/ejhg.2014.180 PMC4795051

[72] Ormond, K. E. et al. Genetic counseling globally: where are we now? *Am. J. Med. Genet. C. Semin. Med. Genet.* **178**, 98–107 (2018). 29575600 10.1002/ajmg.c.31607 PMC5947883

[73] Shickh, S. et al. The role of digital tools in the delivery of genomic medicine: enhancing patient-centered care. *Genet. Med.* **23**, 1086–1094 (2021). 33654192 10.1038/s41436-021-01112-1 PMC9306004

[74] Greenberg, S. E., Boothe, E., Delaney, C. L., Noss, R. & Cohen, S. A. Genetic counseling service delivery models in the United States: assessment of changes in use from 2010 to 2017. *J. Genet. Couns.* **29**, 1126–1141 (2020). 32314856 10.1002/jgc4.1265

[75] Kurian, A. W. et al. Genetic testing and counseling among patients with newly diagnosed breast cancer. *JAMA* **317**, 531–534 (2017). 28170472 10.1001/jama.2016.16918 PMC5530866

[76] Scott, N., O’Sullivan, J., Asgeirsson, K., Macmillan, D. & Wilson, E. Changing practice: moving to a specialist nurse-led service for BRCA gene testing. *Br. J. Nurs.* **29**, S6–S13 (2020). 32463748 10.12968/bjon.2020.29.10.S6

[77] Dusic, E. J. et al. Socioeconomic status and interest in genetic testing in a US-based sample. *Healthcare* **10**, 880 (2022). 10.3390/healthcare10050880 PMC9141316 35628017

[78] Slomp, C. et al. The stepwise process of integrating a genetic counsellor into primary care. *Eur. J. Hum. Genet.* **30**, 772–781 (2022). 35095102 10.1038/s41431-022-01040-x PMC8801315

[79] Rodriguez, N. J., Ricker, C., Stoffel, E. M. & Syngal, S. Barriers and facilitators to genetic education, risk assessment, and testing: considerations on advancing equitable genetics care. *Clin. Gastroenterol. Hepatol.* **21**, 3–7 (2023). 36549838 10.1016/j.cgh.2022.10.025 PMC10609510

[80] Chapman-Davis, E. et al. Racial and ethnic disparities in genetic testing at a hereditary breast and ovarian cancer center. *J. Gen. Intern. Med.* **36**, 35–42 (2021). 32720237 10.1007/s11606-020-06064-x PMC7859010

[81] Peterson, J. M. et al. Racial disparities in breast cancer hereditary risk assessment referrals. *J. Genet. Couns.* **29**, 587–593 (2020). 32196827 10.1002/jgc4.1250

[82] Cragun, D. et al. Racial disparities in BRCA testing and cancer risk management across a population-based sample of young breast cancer survivors. *Cancer* **123**, 2497–2505 (2017). 28182268 10.1002/cncr.30621 PMC5474124

[83] Hann, K. E. J. et al. Awareness, knowledge, perceptions, and attitudes towards genetic testing for cancer risk among ethnic minority groups: a systematic review. *BMC Public Health* **17**, 503 (2017). 28545429 10.1186/s12889-017-4375-8 PMC5445407

[84] Tatineni, S. et al. Racial and ethnic variation in multigene panel testing in a cohort of BRCA1/2-negative individuals who had genetic testing in a large urban comprehensive cancer center. *Cancer Med.* **11**, 1465–1473 (2022). 35040284 10.1002/cam4.4541 PMC8921894

[85] Caswell-Jin, J. L. et al. Racial/ethnic differences in multiple-gene sequencing results for hereditary cancer risk. *Genet. Med.* **20**, 234–239 (2018). 28749474 10.1038/gim.2017.96

[86] Jones, T. et al. Racial and ethnic differences in BRCA1/2 and multigene panel testing among young breast cancer patients. *J. Cancer Educ.* **36**, 463–469 (2021). 31802423 10.1007/s13187-019-01646-8 PMC7293107

[87] Landry, L. G., Ali, N., Williams, D. R., Rehm, H. L. & Bonham, V. L. Lack of diversity in genomic databases is a barrier to translating precision medicine research into practice. *Health Aff.* **37**, 780–785 (2018). 10.1377/hlthaff.2017.1595 29733732

[88] Evans, D. G. et al. The importance of ethnicity: are breast cancer polygenic risk scores ready for women who are not of White European origin? *Int. J. Cancer* **150**, 73–79 (2022). 34460111 10.1002/ijc.33782 PMC9290473

[89] Schuster, A. L. et al. Priorities to promote participant engagement in the Participant Engagement and Cancer Genome Sequencing (PE-CGS) Network. *Cancer Epidemiol. Biomarers* **32**, 487–495 (2023). 10.1158/1055-9965.EPI-22-0356 PMC10068438 36791345

[90] Canedo, J. R. et al. Barriers and facilitators to dissemination and adoption of precision medicine among Hispanics/Latinos. *BMC Public Health* **20**, 603 (2020). 32357943 10.1186/s12889-020-08718-1 PMC7195743

[91] George, A. et al. Implementing rapid, robust, cost-effective, patient-centred, routine genetic testing in ovarian cancer patients. *Sci. Rep.* **6**, 29506 (2016). 27406733 10.1038/srep29506 PMC4942815

[92] Bokkers, K. et al. The feasibility of implementing mainstream germline genetic testing in routine cancer care-a systematic review. *Cancers* **14**, 1059 (2022). 10.3390/cancers14041059 PMC8870548 35205807

[93] Ip, E. et al. Evaluation of a mainstream genetic testing program for women with ovarian or breast cancer. *Asia Pac. J. Clin. Oncol.* **18**, e414–e419 (2022). 35098668 10.1111/ajco.13741

[94] Bokkers, K. et al. Mainstream germline genetic testing for patients with epithelial ovarian cancer leads to higher testing rates and a reduction in genetics-related healthcare costs from a healthcare payer perspective. *Gynecol. Oncol.* **167**, 115–122 (2022). 36031452 10.1016/j.ygyno.2022.08.011

[95] Bokkers, K. et al. Mainstream genetic testing for women with ovarian cancer provides a solid basis for patients to make a well-informed decision about genetic testing. *Hered. Cancer Clin. Pract.* **20**, 33 (2022). 36076240 10.1186/s13053-022-00238-w PMC9461259

[96] Richardson, M. et al. Oncology clinic-based hereditary cancer genetic testing in a population-based health care system. *Cancers* **12**, 338 (2020). 10.3390/cancers12020338 PMC7072228 32028617

[97] Piedimonte, S. et al. BRCA testing in women with high-grade serous ovarian cancer: gynecologic oncologist-initiated testing compared with genetics referral. *Int. J. Gynecol. Cancer* **30**, 1757–1761 (2020). 32759180 10.1136/ijgc-2020-001261

[98] Rumford, M. et al. Oncologist-led BRCA ‘mainstreaming’ in the ovarian cancer clinic: a study of 255 patients and its impact on their management. *Sci. Rep.* **10**, 3390 (2020). 32098980 10.1038/s41598-020-60149-5 PMC7042365

[99] Lapointe, J. et al. A collaborative model to implement flexible, accessible and efficient oncogenetic services for hereditary breast and ovarian cancer: the C-MOnGene study. *Cancers* **13**, 2729 (2021). 10.3390/cancers13112729 PMC8198545 34072979

[100] Hallowell, N. et al. Moving into the mainstream: healthcare professionals’ views of implementing treatment focussed genetic testing in breast cancer care. *Fam. Cancer* **18**, 293–301 (2019). 30689103 10.1007/s10689-019-00122-y PMC6560008

[101] Isselhard, A. et al. Implementation and evaluation of a nurse-led decision-coaching program for healthy breast cancer susceptibility gene (BRCA1/2) mutation carriers: a study protocol for the randomized controlled EDCP-BRCA study. *Trials* **21**, 501 (2020). 32513307 10.1186/s13063-020-04431-x PMC7278068

[102] Berger-Hoger, B. et al. Nurse-led decision coaching by specialized nurses for healthy BRCA1/2 gene mutation carriers - adaptation and pilot testing of a curriculum for nurses: a qualitative study. *BMC Nurs.* **21**, 42 (2022). 35139834 10.1186/s12912-022-00810-8 PMC8829999

[103] Wright, S. et al. Patients’ views of Treatment-Focused Genetic Testing (TFGT): some lessons for the mainstreaming of BRCA1 and BRCA2 testing. *J. Genet. Couns.* **27**, 1459–1472 (2018). 29752676 10.1007/s10897-018-0261-5 PMC6209051

[104] Nilsson, M. P. et al. High patient satisfaction with a simplified BRCA1/2 testing procedure: long-term results of a prospective study. *Breast Cancer Res. Treat.* **173**, 313–318 (2019). 30311024 10.1007/s10549-018-5000-y PMC6394590

[105] Gleeson, M. et al. Communication and information needs of women diagnosed with ovarian cancer regarding treatment-focused genetic testing. *Oncol. Nurs. Forum* **40**, 275–283 (2013). 23619104 10.1188/13.ONF.40-03AP

[106] Hoberg-Vetti, H. et al. BRCA1/2 testing in newly diagnosed breast and ovarian cancer patients without prior genetic counselling: the DNA-BONus study. *Eur. J. Hum. Genet.* **24**, 881–888 (2016). 26350514 10.1038/ejhg.2015.196 PMC4867439

[107] Cragun, D. et al. Differences in BRCA counseling and testing practices based on ordering provider type. *Genet. Med.* **17**, 51–57 (2015). 24922460 10.1038/gim.2014.75 PMC4264999

[108] Fallowfield, L. et al. Talking about Risk, Uncertainties of Testing IN Genetics (TRUSTING): development and evaluation of an educational programme for healthcare professionals about BRCA1 & BRCA2 testing. *Br. J. Cancer* **127**, 1116–1122 (2022). 35715636 10.1038/s41416-022-01871-x PMC9470577

[109] Lee, W. et al. Patient-facing digital tools for delivering genetic services: a systematic review. *J. Med. Genet.* **60**, 1–10 (2023). 36137613 10.1136/jmg-2022-108653

[110] Bombard, Y. & Hayeems, R. Z. How digital tools can advance quality and equity in genomic medicine. *Nat. Rev. Genet.* **21**, 505–506 (2020). 32601319 10.1038/s41576-020-0260-x PMC7322700

[111] Torr, B. et al. LBA101 BRCA-DIRECT: a randomised UK study evaluating a digital pathway for germline genetic testing and non-inferiority of digitally-delivered information in women with breast cancer. *Ann. Oncol.* **34**, S1339 (2023). 10.1016/j.annonc.2023.10.105

[112] Walsh, E. M. et al. Olaparib use in patients with metastatic breast cancer harboring somatic BRCA1/2 mutations or mutations in non-BRCA1/2, DNA damage repair genes. *Clin. Breast Cancer* **22**, 319–325 (2022). 35074264 10.1016/j.clbc.2021.12.007

[113] Tung, N. M. et al. TBCRC 048: phase II study of olaparib for metastatic breast cancer and mutations in homologous recombination-related genes. *J. Clin. Oncol.* **38**, 4274–4282 (2020). 33119476 10.1200/JCO.20.02151

[114] Batalini, F. et al. Analysis of real-world (RW) data for metastatic breast cancer (mBC) patients (pts) with somatic BRCA1/2 (sBRCA) or other homologous recombination (HR)-pathway gene mutations (muts) treated with PARP inhibitors (PARPi). *J. Clin. Oncol.* **39**, 10512–10512 (2021). 10.1200/JCO.2021.39.15_suppl.10512

[115] Hodgson, D. R. et al. Concordance of BRCA mutation detection in tumor versus blood, and frequency of bi-allelic loss of BRCA in tumors from patients in the phase III SOLO2 trial. *Gynecol. Oncol.* **163**, 563–568 (2021). 34742578 10.1016/j.ygyno.2021.10.002

[116] Blum, J. L. et al. Determinants of response to talazoparib in patients with HER2-negative, germline BRCA1/2-mutated breast cancer. *Clin. Cancer Res.* **28**, 1383–1390 (2022). 35091441 10.1158/1078-0432.CCR-21-2080 PMC9365365

[117] Bekos, C. et al. Reliability of tumor testing compared to germline testing for detecting BRCA1 and BRCA2 mutations in patients with epithelial ovarian cancer. *J. Pers. Med.* **11**, 593 (2021). 10.3390/jpm11070593 PMC8305542 34202525

[118] Callens, C. et al. Concordance between tumor and germline BRCA status in high-grade ovarian carcinoma patients in the phase III PAOLA-1/ENGOT-ov25 trial. *J. Natl Cancer Inst.* **113**, 917–923 (2021). 33372675 10.1093/jnci/djaa193 PMC8246800

[119] Hodgson, D. et al. Analysis of mutation status and homologous recombination deficiency in tumors of patients with germline BRCA1 or BRCA2 mutations and metastatic breast cancer: OlympiAD. *Ann. Oncol.* **32**, 1582–1589 (2021). 34500047 10.1016/j.annonc.2021.08.2154

[120] Rivera, D. et al. Implementing NGS-based BRCA tumour tissue testing in FFPE ovarian carcinoma specimens: hints from a real-life experience within the framework of expert recommendations. *J. Clin. Pathol.* **74**, 596–603 (2021). 32895300 10.1136/jclinpath-2020-206840

[121] Polak, P. et al. A mutational signature reveals alterations underlying deficient homologous recombination repair in breast cancer. *Nat. Genet.* **49**, 1476–1486 (2017). 28825726 10.1038/ng.3934 PMC7376751

[122] Lai, Z. et al. Landscape of homologous recombination deficiencies in solid tumours: analyses of two independent genomic datasets. *BMC Cancer* **22**, 13 (2022). 34979999 10.1186/s12885-021-09082-y PMC8722117

[123] Zehir, A. et al. Mutational landscape of metastatic cancer revealed from prospective clinical sequencing of 10,000 patients. *Nat. Med.* **23**, 703–713 (2017). 28481359 10.1038/nm.4333 PMC5461196

[124] US Food and Drug Administration. Alpelisib in combination with fulvestrant for the treatment of PIK3CA-mutated, advanced or metastatic breast cancer - approval letter. Available from https://www.accessdata.fda.gov/drugsatfda_docs/nda/2019/212526Orig1s000Approv.pdf (Accessed August 2024), 2019.

[125] US Food and Drug Administration. Capivasertib in combination with fulvestrant for the treatment of advanced or metastatic breast cancer with one or more PIK3CA/AKT1/PTEN-alteration - approval letter. Available from https://www.accessdata.fda.gov/drugsatfda_docs/appletter/2023/218197Orig1s000ltr.pdf (Accessed August 2024), 2023.

[126] US Food and Drug Administration. FDA approves pembrolizumab for adults and children with TMB-H solid tumors. Available from https://www.fda.gov/drugs/drug-approvals-and-databases/fda-approves-pembrolizumab-adults-and-children-tmb-h-solid-tumors (Accessed August 2024), 2020.

[127] Liu, Y. L. & Stadler, Z. K. The future of parallel tumor and germline genetic testing: is there a role for all patients with cancer? *J. Natl Compr. Cancer Netw.* **19**, 871–878 (2021). 10.6004/jnccn.2021.7044 PMC11123333 34340209

[128] Green, M. F. et al. Concordance between genomic alterations detected by tumor and germline sequencing: results from a tertiary care academic center molecular tumor board. *Oncologist* **28**, 33–39 (2023). 35962742 10.1093/oncolo/oyac164 PMC9847540

[129] Slavin, T. P. et al. Identification of incidental germline mutations in patients with advanced solid tumors who underwent cell-free circulating tumor DNA sequencing. *J. Clin. Oncol.* **36**, JCO1800328 (2018). 30339520 10.1200/JCO.18.00328 PMC6286162

[130] Anaclerio, F. et al. Clinical usefulness of NGS multi-gene panel testing in hereditary cancer analysis. *Front. Genet.* **14**, 1060504 (2023). 37065479 10.3389/fgene.2023.1060504 PMC10104445

[131] Comeaux, J. G. et al. Risk-reducing mastectomy decisions among women with mutations in high- and moderate- penetrance breast cancer susceptibility genes. *Mol. Genet. Genom. Med.* **10**, e2031 (2022). 10.1002/mgg3.2031 PMC9544212 36054727

[132] Breast Cancer Association, Consortium et al. Breast cancer risk genes - association analysis in more than 113,000 women. N. Engl. J.Med. **384**, 428–439 (2021).. 10.1056/NEJMoa1913948 PMC7611105 33471991

[133] Gruber, J. J. et al. A phase II study of talazoparib monotherapy in patients with wild-type BRCA1 and BRCA2 with a mutation in other homologous recombination genes. *Nat. Cancer* **3**, 1181–1191 (2022). 36253484 10.1038/s43018-022-00439-1 PMC9586861

[134] Eikesdal, H. P. et al. Olaparib monotherapy as primary treatment in unselected triple negative breast cancer. *Ann. Oncol.* **32**, 240–249 (2021). 33242536 10.1016/j.annonc.2020.11.009

[135] Tsoulaki, O. et al. Joint ABS-UKCGG-CanGene-CanVar consensus regarding the use of CanRisk in clinical practice. *Br. J. Cancer* **130**, 2027–2036 (2024). 10.1038/s41416-024-02733-4 PMC11183136 38834743

[136] Carver, T. et al. CanRisk tool-A web interface for the prediction of breast and ovarian cancer risk and the likelihood of carrying genetic pathogenic variants. *Cancer Epidemiol. Biomarkers Prev.* **30**, 469–473 (2021). 33335023 10.1158/1055-9965.EPI-20-1319 PMC7611188

