# Supplementary material for: BRCA genetic testing and counseling in breast cancer: how do we meet our patients’ needs?
Source: NPJ Breast Cancer. 2024 Sep 5;10:77. doi: 10.1038/s41523-024-00686-8 (PMC11377442; doi:10.1038/s41523-024-00686-8)

Understanding germline BRCA testing and its clinical implications in breast cancer

This educational material provides healthcare professionals with an overview of the importance of germline BRCA testing and its clinical implications in early and metastatic breast cancer.

DoP: March 2022

JBN: Z4-41806

Disclaimer: This proactive material for local market use only following review with local nominated signatory

Developed by HCPs for HCPs

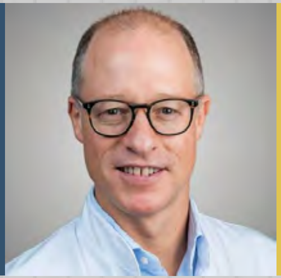

Peter Dubsky
Surgeon
Hirslanden Klinik
St. Anna, Lucerne,
Switzerland

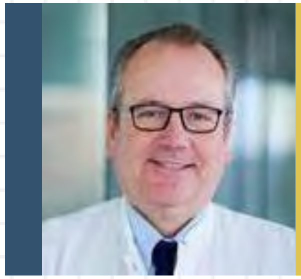

Christian Jackisch
Gynaecological Oncologist
Sana Klinikum Offenbach
Offenbach am Main,
Germany

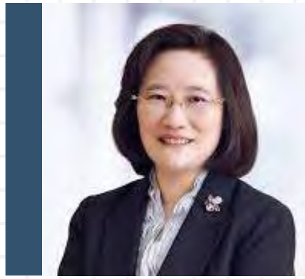

Seock-Ah Im
Medical Oncologist
Seoul National University
Seoul, Rep. of Korea

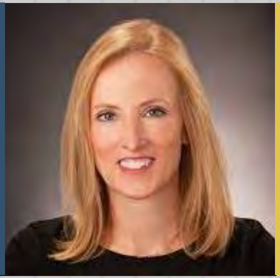

Kelly Hunt
Surgeon
MD Anderson
Cancer Center,
Houston, Texas, USA

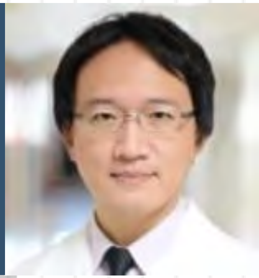

Chien-Feng Li
Pathologist
Chi Mei Medical Center
Tainan, Taiwan

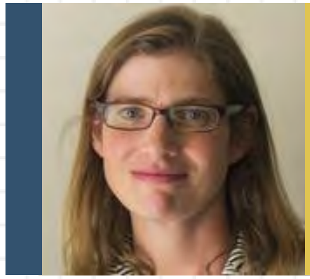

Sheila Unger
Geneticist
Lausanne University
Hospital
Lausanne, Switzerland

What's included?

- Prognostic implications of germline mutations in breast cancer
- The prevalence of germline BRCA (gBRCA) mutations in breast cancer
- Characteristics of breast cancers with a gBRCA mutation
- gBRCA mutation status informing a patient's care plan
- Wider implications of gBRCA testing

In this resource the term "gBRCA mutation" refers to germline pathogenic and likely pathogenic variants in the *BRCA1* and *BRCA2* genes that have potential clinical implications

If a patient harbours a variant of unknown significance (VUS), it should be reassessed by a genetics laboratory every ~3 years to determine whether the VUS has been reclassified

BRCA mutations have implications beyond breast cancer

Mutations in *BRCA1* or *BRCA2* genes are associated with an increased risk of cancer in both men and women compared to the general population, including:[1]

- ♀♂ Breast cancer
- ♂ Prostate cancer
- ♀ Ovarian cancer
- ♀♂ Pancreatic cancer

Patients with gBRCA mutations can develop breast cancer of any subtype

Most breast cancer patients have **HR-positive, HER2-negative disease**[2]

Twice as many gBRCA mutation carriers have HR-positive, HER2-negative breast cancer compared with TNBC (HR-negative, HER2-negative)[3,4]

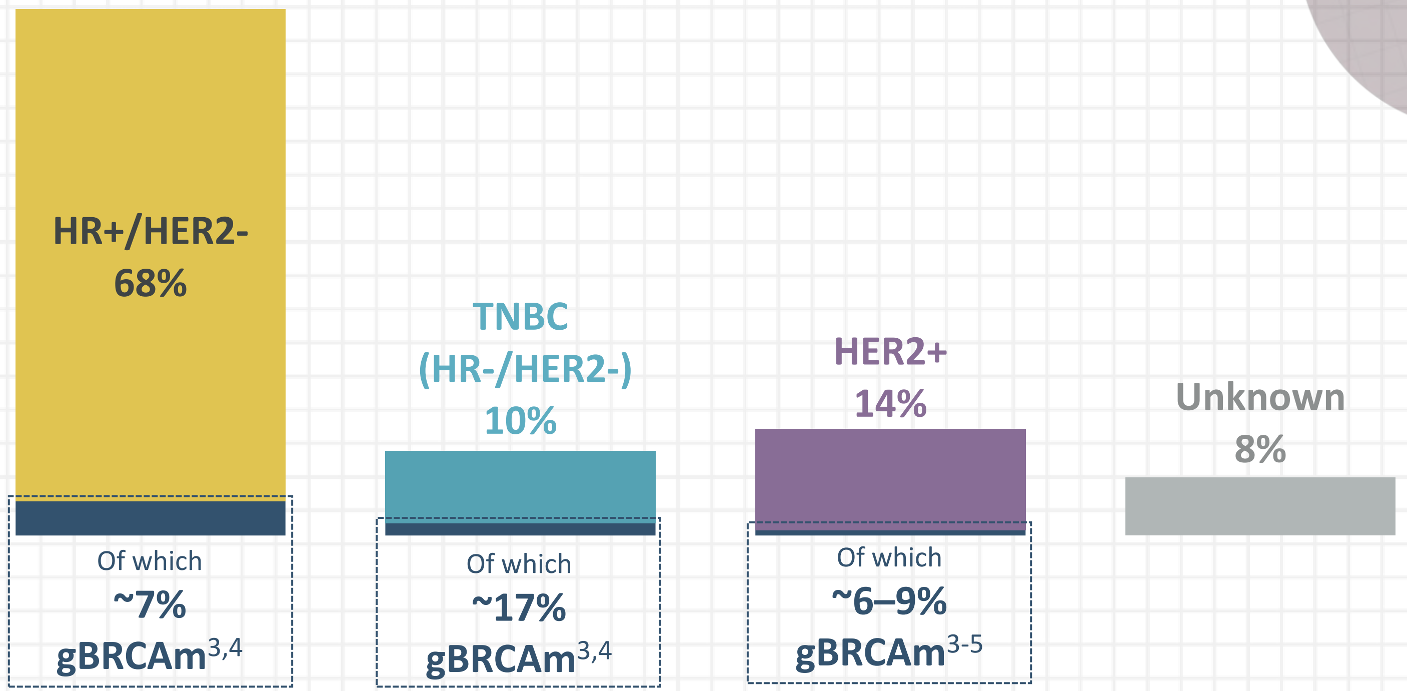

The prevalence of gBRCA mutations is higher in patients with a family history of breast and/or ovarian cancer[6]

***Please note these schematics are representative only**
HR=hormone receptor; HER2=human epidermal growth factor receptor 2; gBRCA(m)=germline BRCA (mutation); TNBC=triple-negative breast cancer.

Breast cancers with gBRCA mutations may behave differently than sporadic breast cancers

Breast cancer with a gBRCA mutation is often associated with:

- Younger age at diagnosis[7]
- Higher risk of developing metastatic disease[8]
- Higher risk of recurrence[9-11]
- Increased risk of second primary malignancies[12]

If the gBRCA status is known early, informed decisions can be made to tailor surgery and systemic treatment for the patient and underlying tumour biology[13-15]

gBRCAm=germline BRCA mutation.

Knowledge of gBRCA status prior to surgery is important to guide surgical decision-making

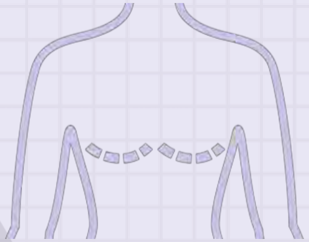

Additional risk-reducing surgeries such as contralateral mastectomy may be adopted over partial mastectomy or unilateral mastectomy for patients who are gBRCA mutation carriers. This can **reduce the risk of recurrent or new breast cancers**[13]

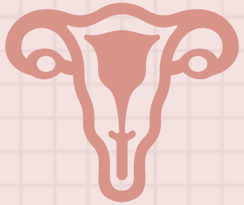

Patients with a gBRCA mutation may opt for **removal of fallopian tubes and ovaries** (salpingo-oophorectomy) at some point after diagnosis depending on age, family planning, hormonal status and ovarian cancer risk[16]

gBRCAm=germline BRCA mutation.

Systemic treatment decisions can be impacted by gBRCA status in both the early and advanced disease settings

gBRCA mutated breast cancer may be more sensitive and responsive to DNA-damaging agents such as cytotoxic chemotherapy[17,18]

Patients with gBRCA mutated breast cancer may be eligible for targeted therapy with PARP inhibitors*[14,19,20]

*Please check local approvals

gBRCA=germline BRCA; PARP=poly(ADP-ribose) polymerase.

Future cancers may be prevented through testing and identification of breast cancer patients with gBRCA mutations

If a patient is found to carry a gBRCA mutation, they may be offered:[21]

Close monitoring and follow-up, including breast cancer screening

Risk-reducing surgeries, where indicated and agreed with the patient

Their family can also be offered cascade testing which can identify those who are at increased risk of BRCA-related cancers, allowing for risk-reducing measures to be put in place, as well as informing family planning decisions[22-24]

gBRCA=germline BRCA.

Many guidelines fail to identify the majority of patients with breast cancer that carry a gBRCA mutation[25,26]

There is **wide variation in guidelines** regarding who is eligible for gBRCA mutation testing. Eligibility criteria often focus on a number of factors including:[22,27,28]

- Family history of BRCA-associated cancers
- Age at diagnosis
- Subtype of breast cancer e.g. TNBC

Testing is also often recommended for patients who may benefit from a targeted therapy such as a PARP inhibitor[22,28]

Studies have shown that guidelines may miss between **20% to 77% of gBRCA mutation carriers as they would not meet restrictive testing criteria**[25,26]

gBRCA=germline BRCA; PARP=poly(ADP ribose) polymerase; TNBC=triple-negative breast cancer.

gBRCA testing offers the opportunity to personalise care for the patient and their family

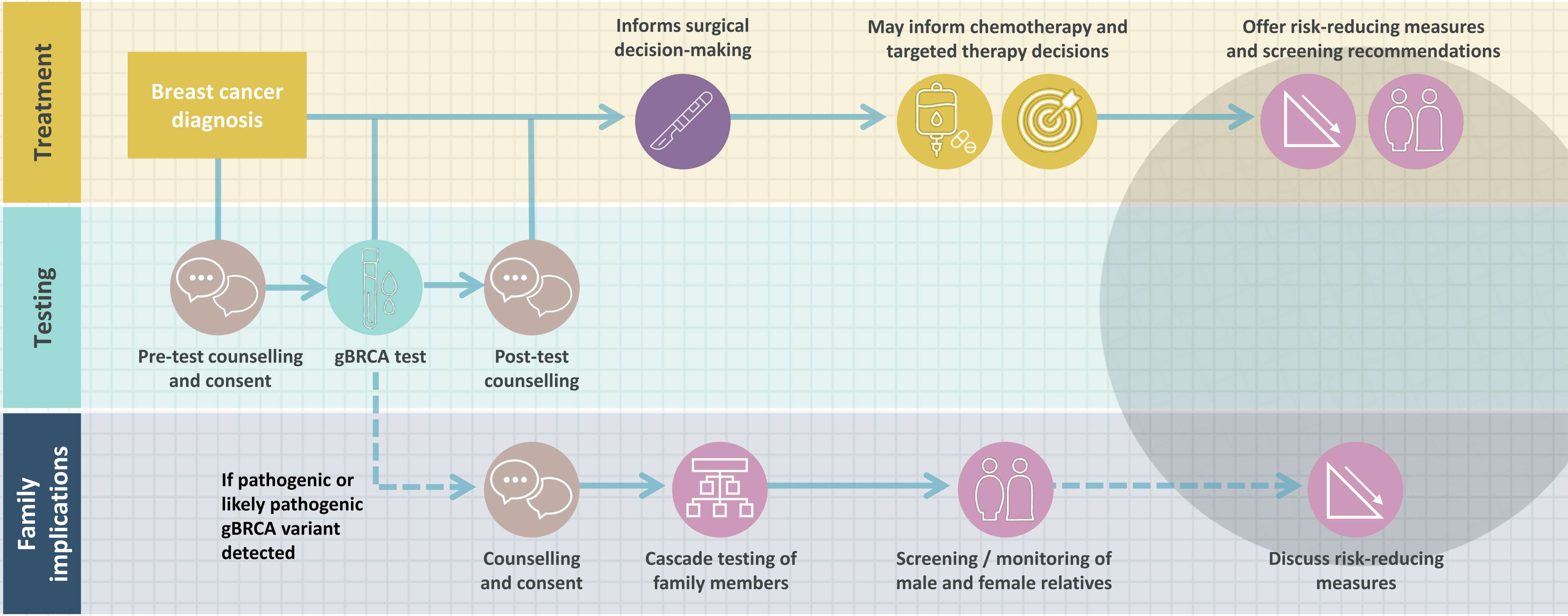

gBRCA=germline BRCA.

For more information, please visit ID.BRCA.com

Introducing the latest insights in BRCA testing for breast cancer

In the past, BRCA testing was mainly used to assess hereditary risk. But now, BRCA testing has moved on and can be used intelligently to inform crucial treatment decisions.

Discover more about who, when and how to test below

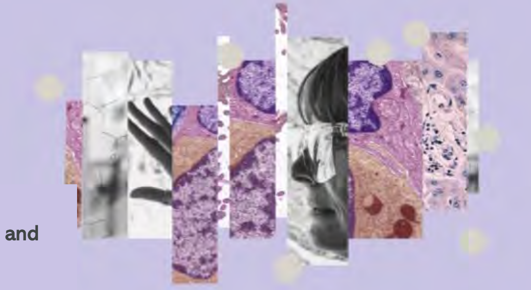

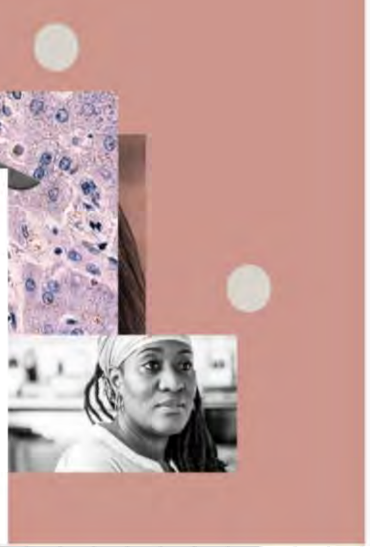

Why do I need to perform early BRCA testing?

BRCA mutation testing can reveal important information about familial risk and help to inform potential treatment options of BRCA-related cancers.

Test routinely to inform treatment decisions.

Read More

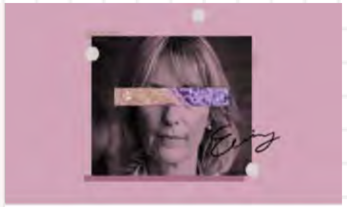

Who needs early BRCA testing?

Testing Patients' BRCA mutation status based on their age, disease subtype and family history can help inform treatment strategies.

Numerous factors impact eligibility for BRCA testing.

Read More

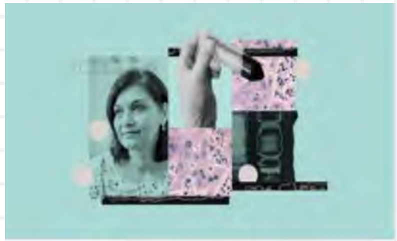

How do I conduct BRCA testing?

In breast cancer, genetic testing generally involves testing for germline BRCA1/2 mutations in BRCA in addiction to other genes.

Appropriate testing can help determine treatment options.

Read More

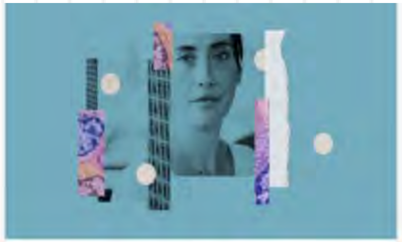

How do I interpret the results of a BRCA test and what does it mean for patients?

Genetic test results can be challenging to interpret due to the large number of genetic variations in tumour DNA, which are unique to any given patient. Proper understanding and communication of test results are critical to patient care.

BRCA result can impact prognosis and treatment options.

Read More

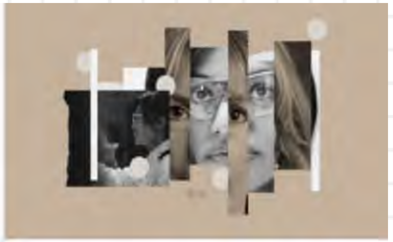

How can genetic counselling be streamlined by the multidisciplinary team?

Test turnaround times and genetic counselling can be barriers to BRCA testing. The oncogenetic pathway can provide patients earlier access to counselling.

The oncogenetic pathway aims to streamline testing

Read More

References

1. American Cancer Society. Genetic counselling and testing for breast cancer risk. Available from: https://www.cancer.org/cancer/breast-cancer/risk-and-prevention/genetic-testing.html. Accessed February 2022.
2. National Cancer Institute SEER. Cancer Stat Facts: Female Breast Cancer Subtypes. Available from: https://seer.cancer.gov/statfacts/html/breast-subtypes.html. Accessed February 2022.
3. Winter C, et al. *Ann Oncol*. 2016;27(8):1532-1538: Supplementary Appendix.
4. Cancer Genome Atlas Network. *Nature*. 2012 Oct 4;490(7418):61-70.
5. Klocker EV and Suppan C. *Breast Care*. 2020;15:586-593.
6. Kast K, et al. *J Med Genet*. 2016;53:465–471.
7. Becourt S, et al. *J Clin Oncol*. 2018;36(suppl; abstr e13522).
8. Mailliez, A, et al. Poster presented at: SABCS Virtual Symposium, December 8-11, 2020. Abstract PD10-08.
9. Force J, et al. Abstract presented at: SABCS San Antonio, Texas. December 4-8, 2018. Abstract P3-08-07.
10. Quek R, et al. *J Clin Oncol.* 2018;36(suppl; abstr e12575).
11. Liu M, Wang S. Abstract presented at: SABCS San Antonio, Texas. December 10-14, 2019. Abstract P3-08-51.
12. Menes TS, et al. *Breast Cancer Res Treat*. 2015;151:653-660.
13. Yadav S, et al. *Hered Cancer Clin Pract*. 2017;15:11.
14. Robson M, et al. *N Engl J Med*. 2017;377(6):523-533.
15. Dieras V, et al. *Lancet Oncol.* 2020;21(10):1269-1282.
16. Metcalfe K, et al. *JAMA Oncol.* 2015;1(3):306-313.
17. Tung, et al. Presented at SABCS San Antonio, Texas. December 10-14, 2019.
18. De Talhouet S, et al. *Sci Rep.* 2020;10:7073.
19. Litton et al, *N Engl J Med*. 2018;379(8):753-763
20. Tutt A, et al. *N Engl J Med.* 2021;384(25):2394-2405.
21. Centers for Disease Control and Prevention. Medical Options for Women with BRCA1 and BRCA2 Mutations. Available from: https://www.cdc.gov/genomics/disease/breast_ovarian_cancer/medical_options.htm. Accessed February 2022.
22. NCCN Guidelines Genetic/Familial High-Risk Assessment: Breast, Ovarian and Pancreatic. V1.2022.
23. Gaba F, et al. *J Med Genet.* 2022;59(2):122-132.
24. Paluch-Shimon S, et al. *Ann Oncol*. 2016;27:103-110.
25. Pujol P, et al. *Eur J Cancer.* 2021;146:30e47.
26. Li J, et al. *Int J Cancer*. 2019;144:1195–1204.
27. Cardoso F, et al. *Ann Oncol*. 2019;30:1194–1220.
28. Gennari A, et al. *Ann Oncol.* 2021;32(12)1475-1495.

What is the process of germline BRCA testing?

Made by **HCPs**, for **HCPs**

The development and funding of this material was funded by AstraZeneca. This is an HCP developed guide aiming to help you as an expert understand the various stages involved within the germline BRCA testing pathway

Developed by HCPs for HCPs

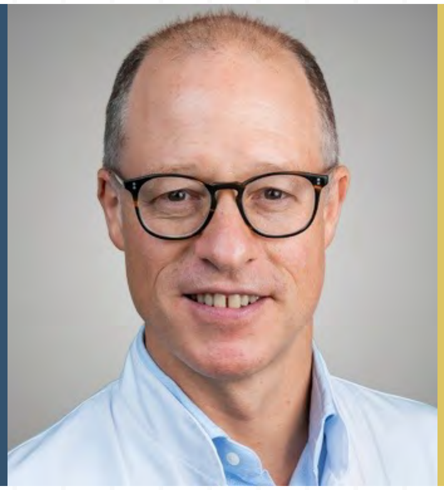

Peter Dubsky
Surgeon
Hirslanden Klinik
St. Anna, Lucerne,
Switzerland

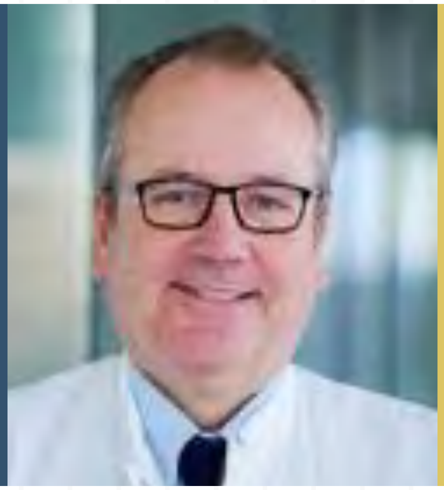

Christian Jackisch
Gynaecological
Oncologist
Sana Klinikum
Offenbach
Offenbach am Main,
Germany

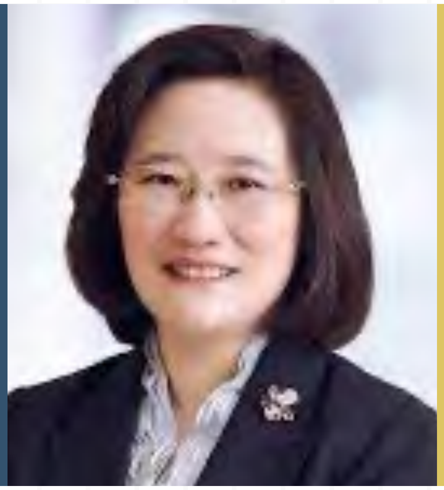

Seock-Ah Im
Medical Oncologist
Seoul National
University
Seoul, Rep. of Korea

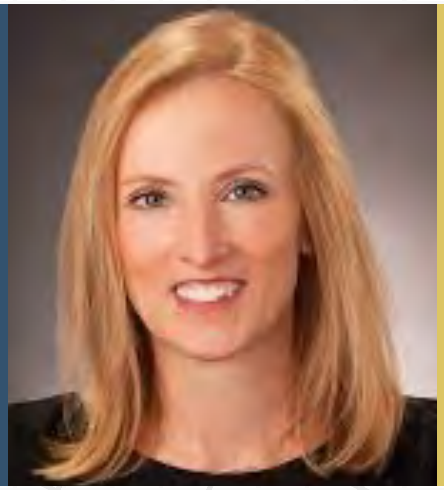

Kelly Hunt
Surgeon
MD Anderson Cancer
Center,
Houston TX, USA

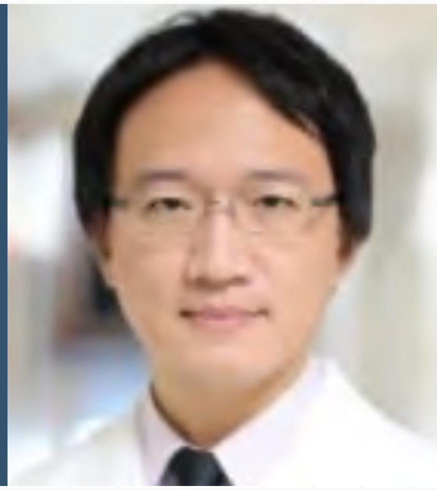

Chien-Feng Li
Pathologist
Chi Mei Medical
Center
Tainan, Taiwan

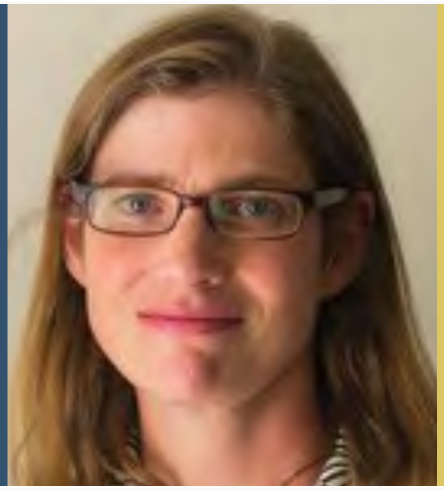

Sheila Unger
Geneticist
Lausanne University
Hospital
Lausanne, Switzerland

Knowledge of germline BRCA mutation status guides treatment decisions and may prevent future cancers

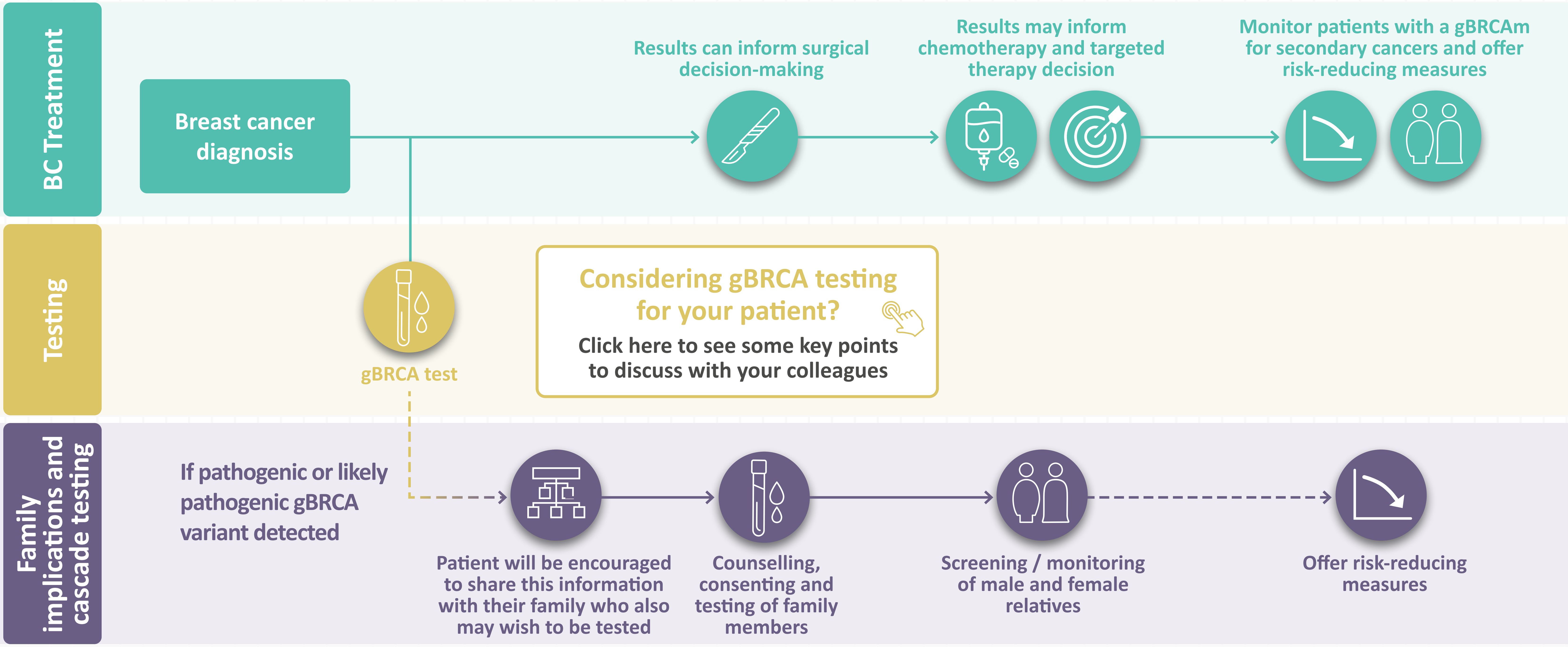

BC=breast cancer; gBRCA=germline BRCA

Knowledge of germline BRCA mutation status guides treatment decisions and may prevent future cancers

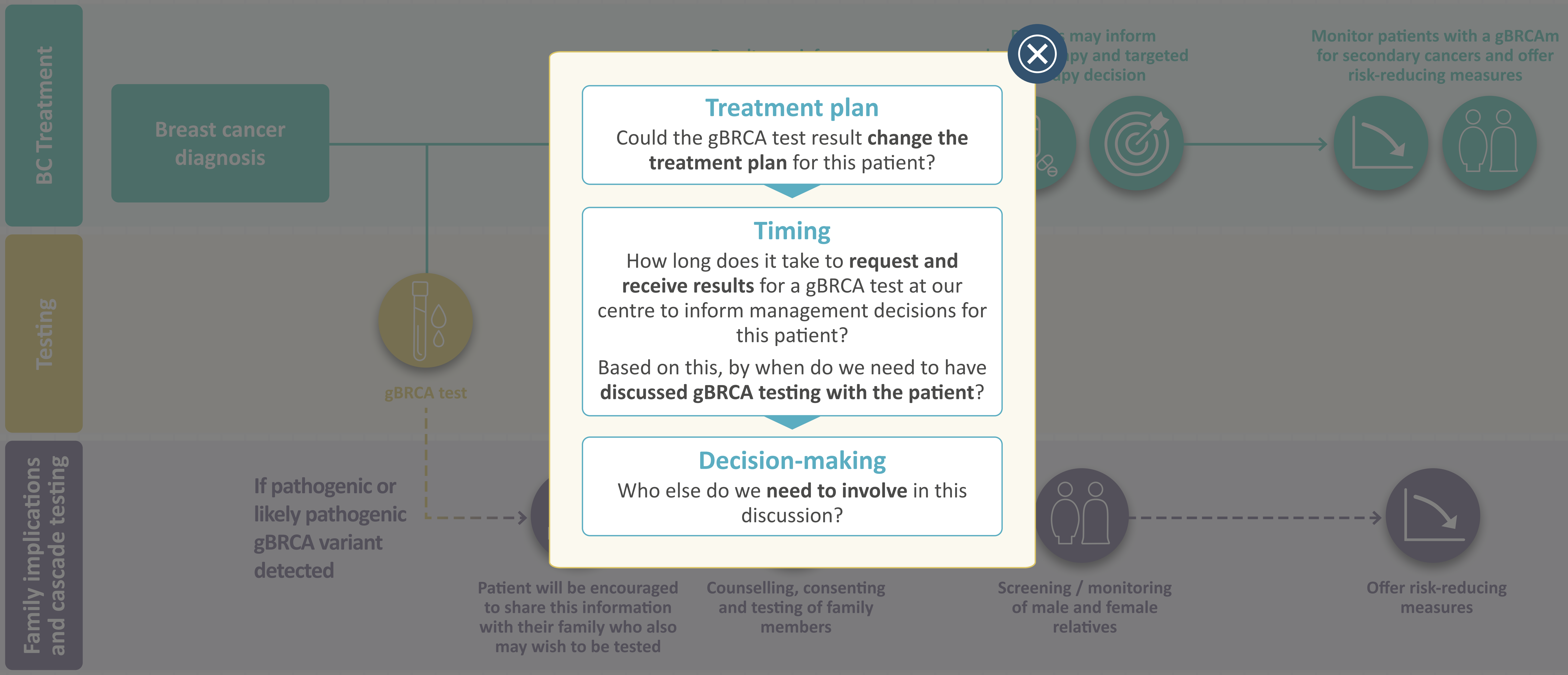

Treatment plan

Could the gBRCA test result **change the treatment plan** for this patient?

Timing

How long does it take to **request and receive results** for a gBRCA test at our centre to inform management decisions for this patient?

Based on this, by when do we need to have **discussed gBRCA testing with the patient**?

Decision-making

Who else do we **need to involve** in this discussion?

BC=breast cancer; gBRCA=germline BRCA

How to use this asset

Click to reveal a pop-up with more information about a specific point in the pathway

Click to exit the pop-up and return to the main pathway

Click to move to the next stage of the pathway

Pathway for germline BRCA testing in breast cancer

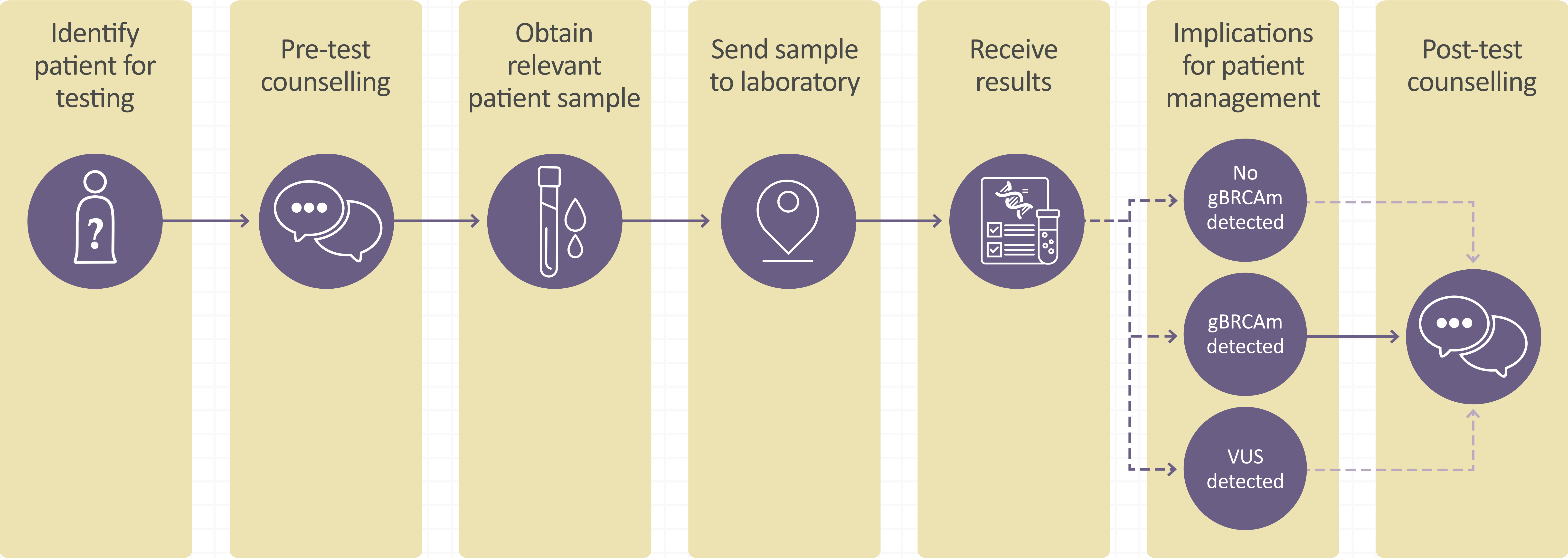

Click on each circle for more information

gBRCAm=germline BRCA mutation; VUS=variant of unknown significance

Pathway for germline BRCA testing in breast cancer

Identify patient for testing

Pre-test

Obtain

Send sample

Receive

Implications

Post-test

Identify patient for testing

Consider which patients with breast cancer you plan to test at your centre

Increasingly testing is being offered to more patients, especially if it can inform treatment decisions or if there is a family history

Some useful guidelines you may wish to review include:

- NCI Cancer Genetics Risk Assessment and Counselling
- NCCN Genetic/Familial High-Risk Assessment: Breast, Ovarian and Pancreatic
- Clinical practice guidelines for BRCA1 and BRCA2 genetic testing (Pujol P et al. *Eur J Cancer*. 2021)
- Germline Genetic Testing for Breast Cancer Risk (Yadav S and Couch FJ. *ASCO Educational Book*. 2019)

Consider in your centre who is responsible for identifying patients who are eligible for testing and who is responsible for requesting a gBRCA test

It could be:

- Medical oncologist
- Surgeon
- Specialist nurse
- Genetic counsellor/geneticist
- Other member of the patient's healthcare team (MDT)

Click to see the next stage in the pathway: Counselling patients on testing

gBRCAm=germline BRCA mutation

Pathway for germline BRCA testing in breast cancer

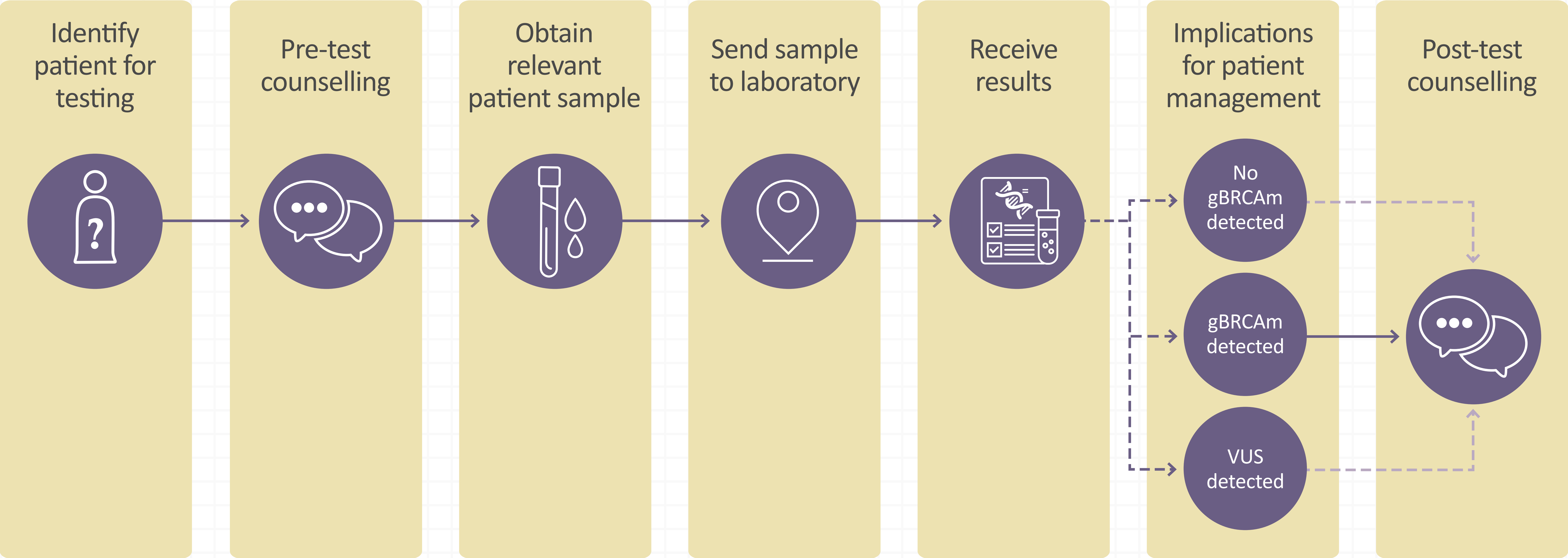

Click on each circle for more information

gBRCAm=germline BRCA mutation; VUS=variant of unknown significance

Pathway for germline BRCA testing in breast cancer

Identify patient for testing

Pre-test counselling

Obtain

Send sample

Receive

Implications

Post-test

Pre-test counselling

Consider who is responsible for counselling patients for a gBRCA test in your centre

This may be the same person that requests the test or someone different

Increasingly oncologists, nurses and surgeons are providing pre-test counselling if the results impact therapeutic decisions

Click to see the next stage in the pathway: Obtaining the appropriate sample for testing

You might need training before you can consent patients.

Find out your local regulations, and consider if the following could be used to train your team:

- Genomics Education Programme
- EDCP-BRCA study protocol
- Health Centre for genetics Education
- Royal College of Physicians

If appropriate, discuss potential costs/ insurance implications of gBRCA testing have a different version to show panels

gBRCAm=germline BRCA mutation
EDCP-BRCA, a German acronym meaning evaluation of a decision coaching program to support preventative decisions of healthy female BRCA1/2 gene mutation carriers

Pathway for germline BRCA testing in breast cancer

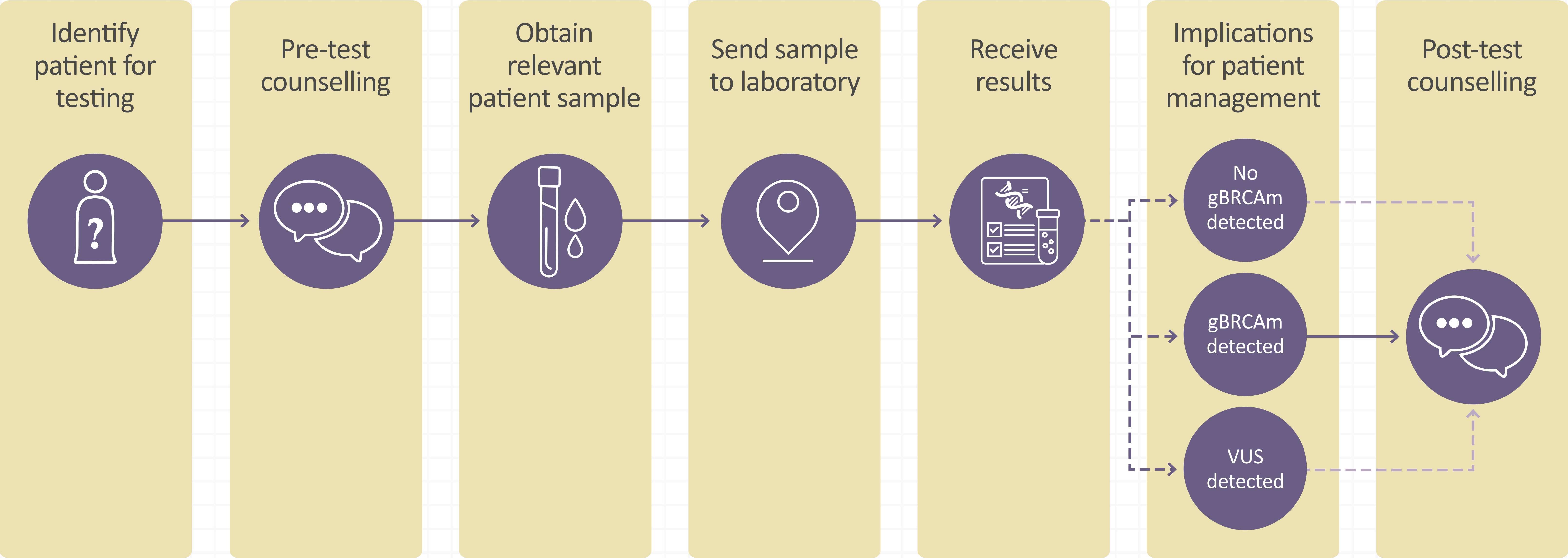

Click on each circle for more information

gBRCAm=germline BRCA mutation; VUS=variant of unknown significance

Pathway for germline BRCA testing in breast cancer

Identify patient for testing

Pre-test counselling

Obtain relevant patient sample

Send sample

Receive

Implications

Post-test

Obtain sample for testing

Patients who are eligible for testing should receive a gBRCA test

Therefore, a blood or buccal sample(s) is the only sample required

Click to see the next stage in the pathway: Sending the sample to the laboratory

gBRCA=germline BRCA

Pathway for germline BRCA testing in breast cancer

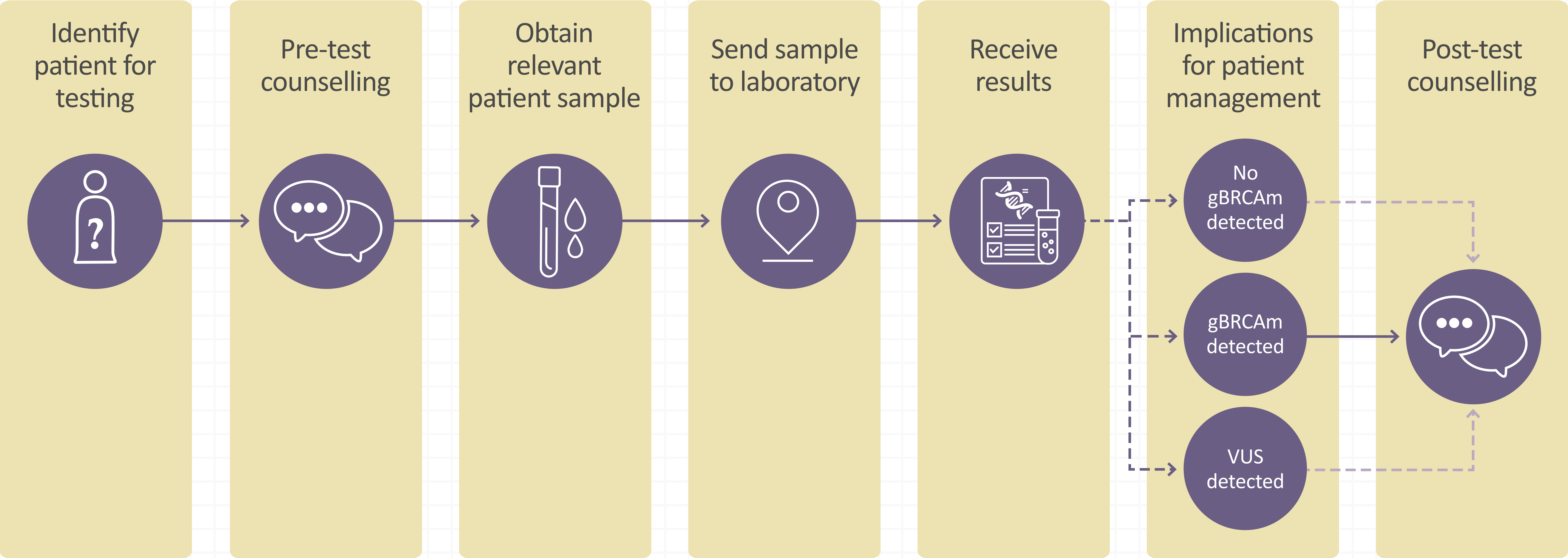

Click on each circle for more information

gBRCAm=germline BRCA mutation; VUS=variant of unknown significance

Pathway for germline BRCA testing in breast cancer

Identify patient for testing

Pre-test counselling

Obtain relevant patient sample

Send sample to laboratory

Receive

Implications [illegible]

Post-test

Send sample to laboratory

There are different approaches for gBRCA testing including:

- ***BRCA1/2* only testing**
- **Multigene panel testing**

- You will need to identify your local testing laboratory and check if they have local approval to carry out gBRCA testing
- The laboratory will be aware of the most appropriate technology to use in order to carry out a gBRCA test
- Check how long it will take for the results to be returned and how you will receive the results

Click to see the next stage in the pathway: Receiving results

gBRCA=germline BRCA

Pathway for germline BRCA testing in breast cancer

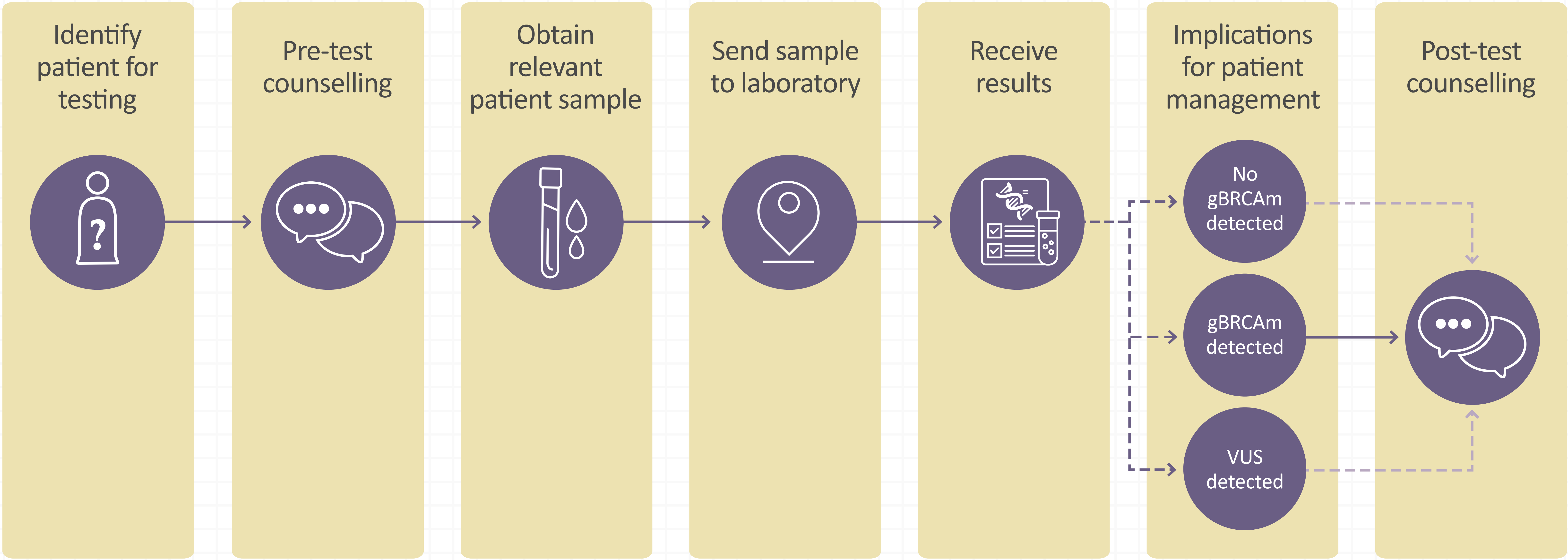

Click on each circle for more information

gBRCAm=germline BRCA mutation; VUS=variant of unknown significance

Pathway for germline BRCA testing in breast cancer

Identify

Pre-test

Obtain

Send sample

Receive results

- Make sure the patient receives the results in a timely manner and understands the implications
- As well as sharing the results, their care plan options should be discussed

Possible results include:

- **No mutation is found**
- **Variant of unknown significance (VUS)**
- **Pathogenic or likely pathogenic gBRCAm**

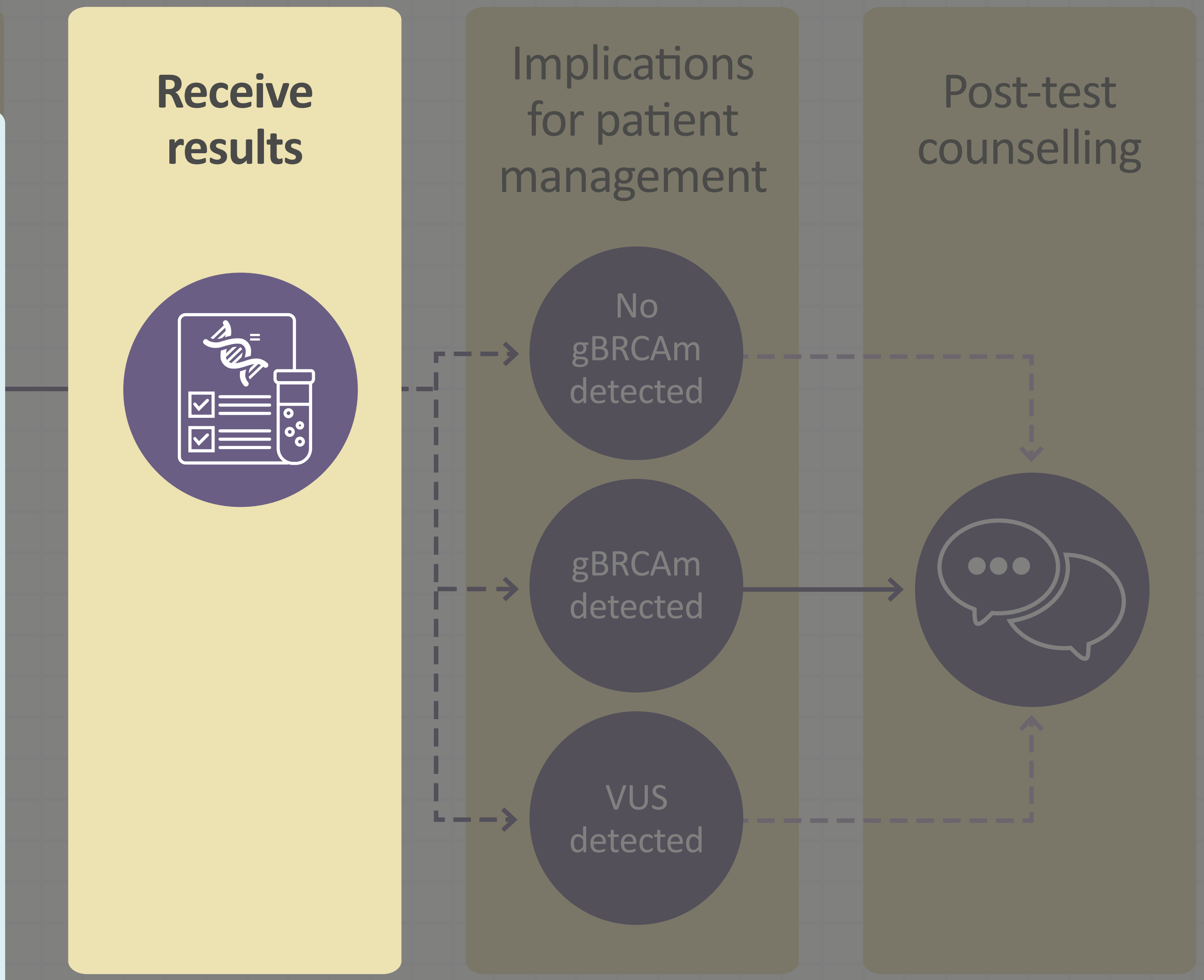

gBRCAm=germline BRCA mutation; VUS=variant of unknown significance

Pathway for germline BRCA testing in breast cancer

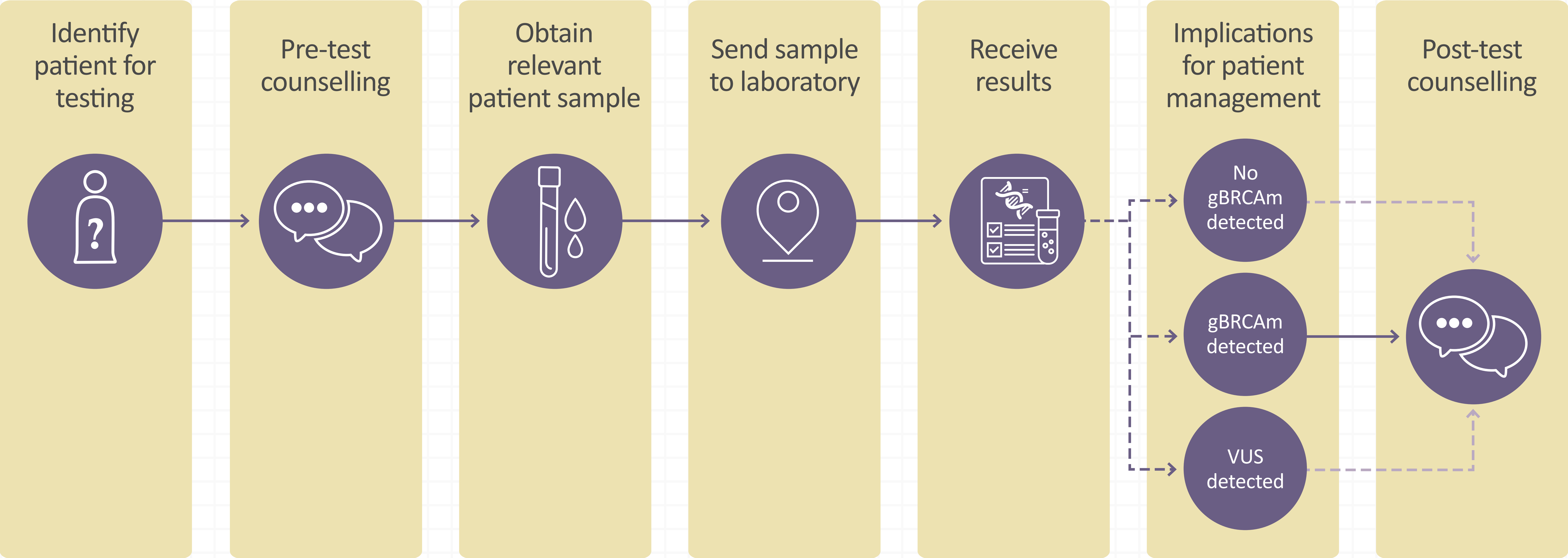

Click on each circle for more information

gBRCAm=germline BRCA mutation; VUS=variant of unknown significance

Pathway for germline BRCA testing in breast cancer

Identify | Pre-test | Obtain | Send sample | Receive

Implications for patient management

- No gBRCAm detected
- gBRCAm detected
- VUS detected

Post-test counselling

Consider implications for patient management

No gBRCAm is detected

- Consider appropriate surgical and treatment options
- Cascade testing for family members is not required
- Mutations in genes other than *BRCA1/2* are known to be implicated in breast cancer, therefore further genetic testing may be valuable if there is a strong family history of breast cancer

Click to see the next stage in the pathway: Post-test genetic counselling

gBRCAm=germline BRCA mutation; VUS=variant of unknown significance

Pathway for germline BRCA testing in breast cancer

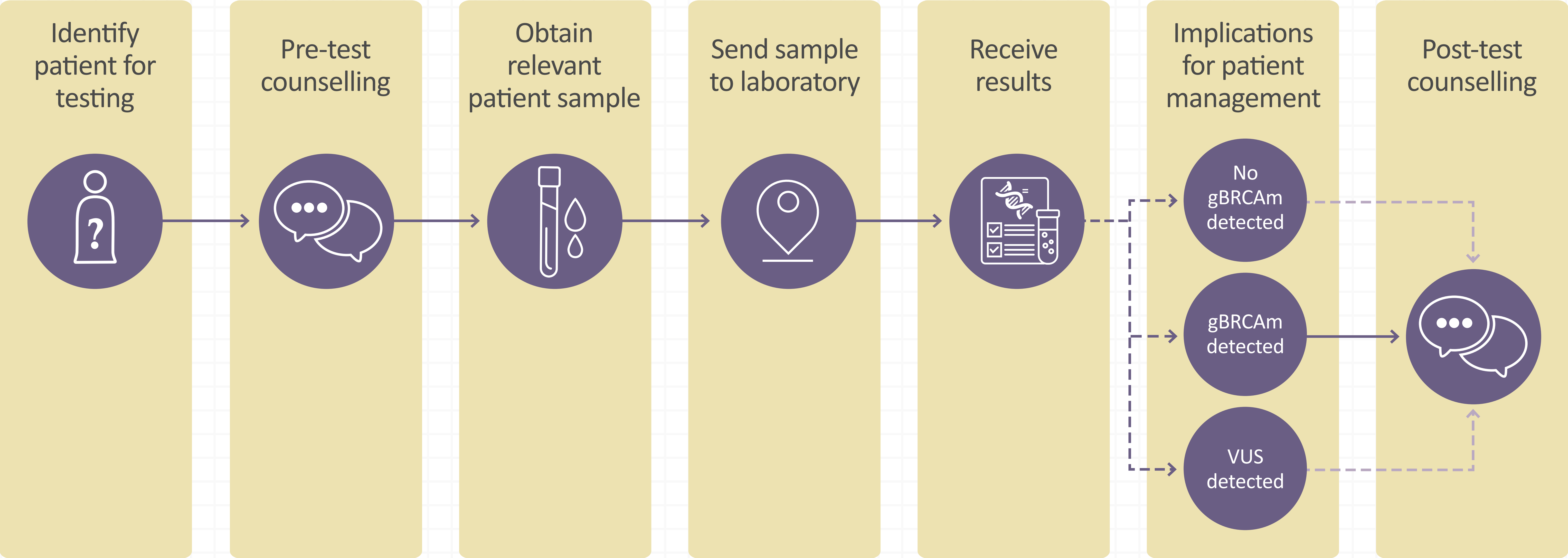

Click on each circle for more information

gBRCAm=germline BRCA mutation; VUS=variant of unknown significance

Pathway for germline BRCA testing in breast cancer

Consider implications for patient management

Pathogenic or likely pathogenic gBRCAm is detected

- Choice of surgical strategy and medical therapy may be impacted
- Risk-reducing surgery such as salpingo-oophorectomy may be offered
- Screening for potential future cancers may be offered
- Cascade testing for family members may be offered

Click to see the next stage in the pathway: Post-test genetic counselling

Pre-test | Obtain | Send sample | Receive | **Implications for patient management** | Post-test counselling

No gBRCAm detected

gBRCAm detected

VUS detected

gBRCAm=germline BRCA mutation; VUS=variant of unknown significance

Pathway for germline BRCA testing in breast cancer

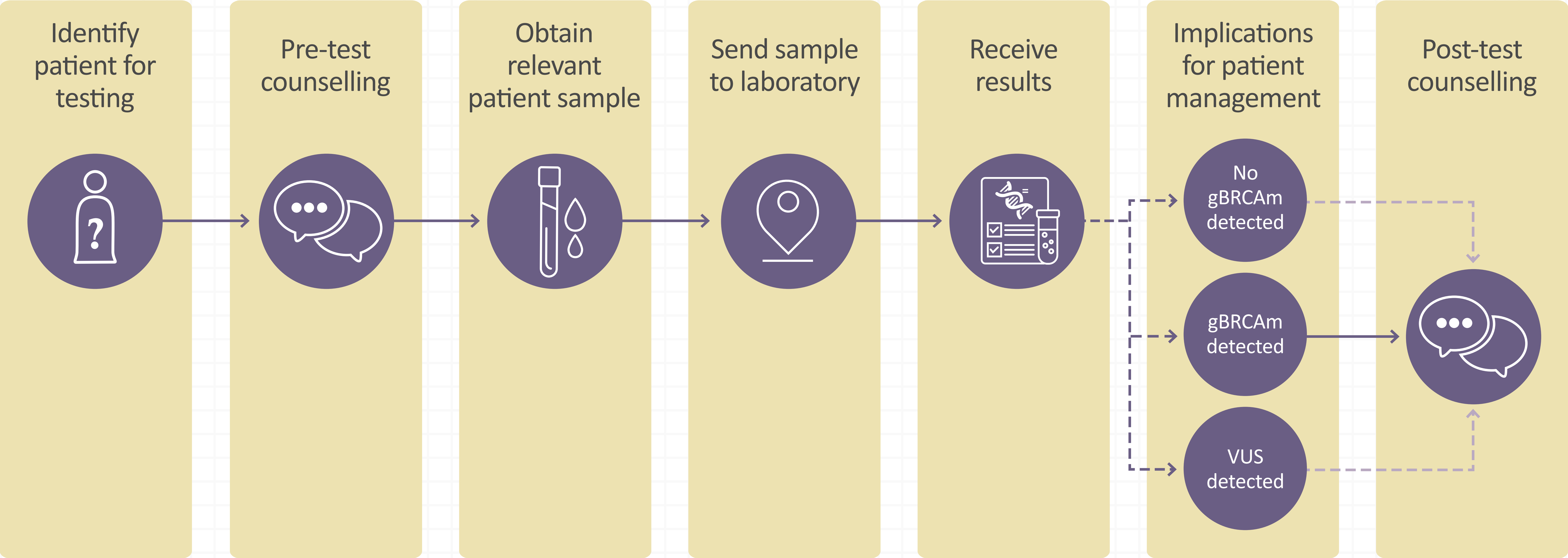

Click on each circle for more information

gBRCAm=germline BRCA mutation; VUS=variant of unknown significance

Pathway for germline BRCA testing in breast cancer

Identify

Pre-test

Obtain

Send sample

Receive

Implications for patient management

No gBRCAm detected

gBRCAm detected

VUS detected

Post-test counselling

Consider implications for patient management

VUS detected

- Consider appropriate surgical and treatment options
- Cascade testing for family members is not required
- VUS may need to be reassessed to determine their clinical relevance in the future
- Mutations in genes other than *BRCA1/2* are known to be implicated in breast cancer, therefore further genetic testing may be valuable if there is a strong family history of breast cancer

gBRCAm=germline BRCA mutation; VUS=variant of unknown significance

Pathway for germline BRCA testing in breast cancer

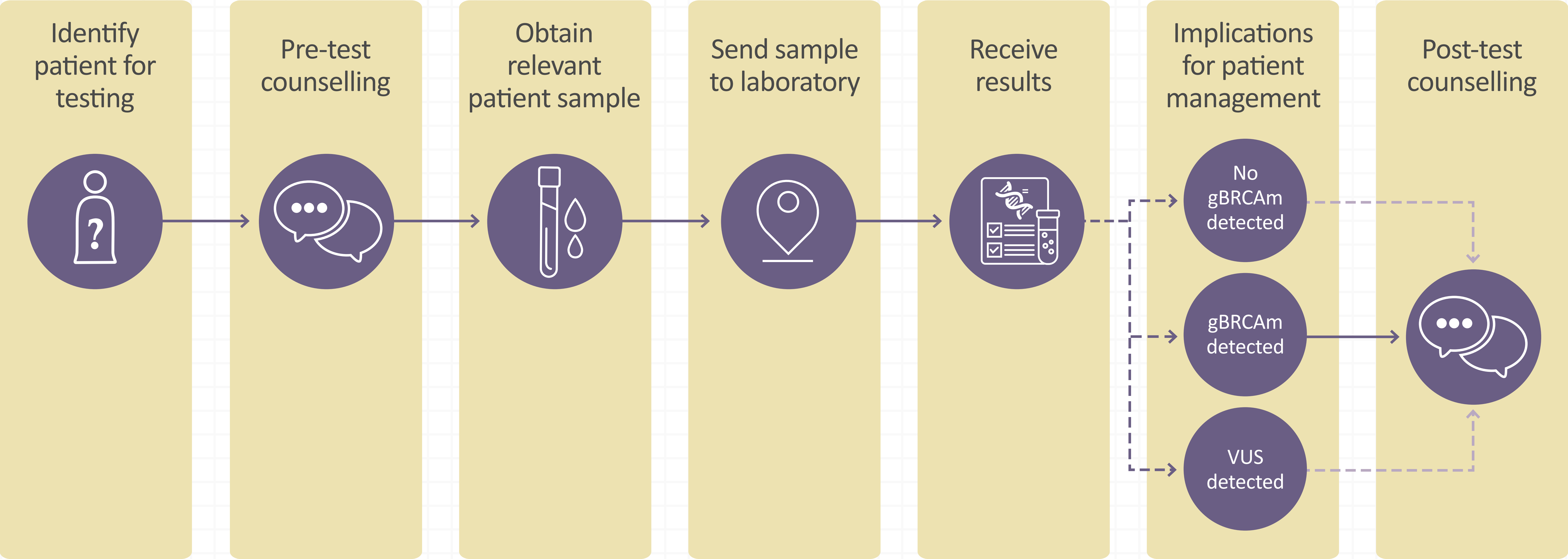

Click on each circle for more information

gBRCAm=germline BRCA mutation; VUS=variant of unknown significance

Pathway for germline BRCA testing in breast cancer

Identify patient for testing

Pre-test counselling

Obtain

Send sample

Receive

Implications

Post-test counselling

Refer patients for further genetic counselling & identification of at-risk family members

- For patients with a gBRCAm, post-test counselling should be provided by a genetic counsellor
- However, post-test counselling may also be required for patients who do not have a gBRCAm
- Testing and counselling should also be offered to family members where appropriate

gBRCAm=germline BRCA mutation

Nurse consenting guide for germline BRCA testing

This educational material provides nurses and other HCPs with an overview of the important role that nurses can play in the germline (g) BRCA testing pathway for patients with breast cancer, with the goal of providing the key information required for nurse-obtained gBRCA testing and consent.

Made by HCPs, for HCPs

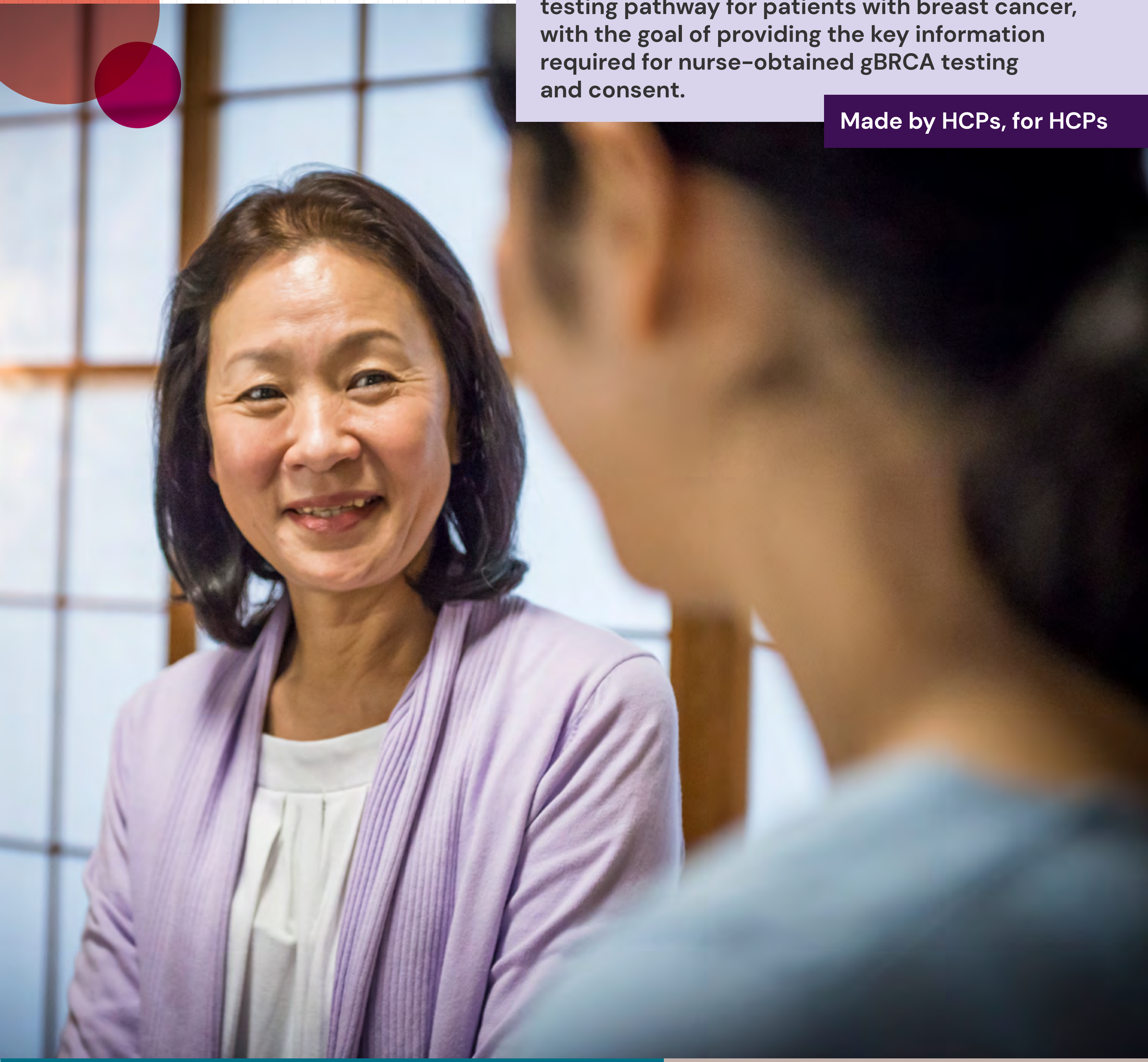

The development and funding of this material was organised by AstraZeneca. This material was built through co-operation between breast cancer physicians and specialist oncological nurses to provide an overview of the nurse role within the BRCA-positive breast cancer patient care pathway.

Z4-49918 | November 2022

What role can nurses play in the gBRCA testing pathway?

As demand for genetic testing for cancer patients increases, alternative genetic testing pathways are required. One approach is to adopt a mainstream consent pathway, whereby a member of the oncology team (rather than a genetic specialist) is responsible for pre-test counselling, consenting, and arranging genetic testing for cancer patients[1]

The benefits of the mainstreaming approach include:[1–3]

- Reduced waiting times for initial gBRCA testing
- Reduced turnaround time for results for patients receiving gBRCA testing
- Freeing up genetic counsellor time for patients who require post-test counselling
- High satisfaction from patients and clinicians

Nurses can play a key role in this process, and nurse-led mainstreaming cancer genetics services have shown:[1–3]

- That clinical nurse specialists are well placed to offer testing
- They are preferred by patients as nurses tend to be more familiar with them and are more likely to understand the pressures the patient is feeling
- That the addition of consenting patients for gBRCA testing was possible within the time allocated for nurse consultations

Nurse-led decision coaching can also enhance shared decision-making for people with breast cancer[4]. It is important for nurses to get involved in this process and advocate for their training in gBRCA testing and consenting

Example gBRCA testing pathway to illustrate potential nurse involvement[4–10]

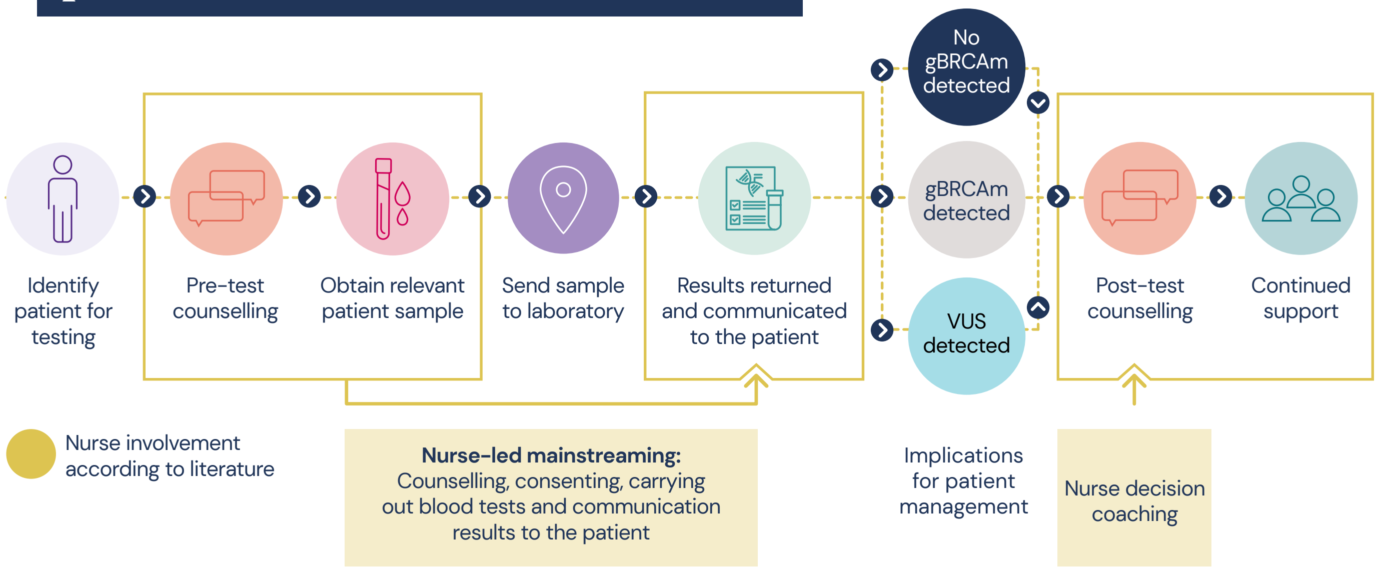

What is the process for genetic counselling, consenting, and testing?

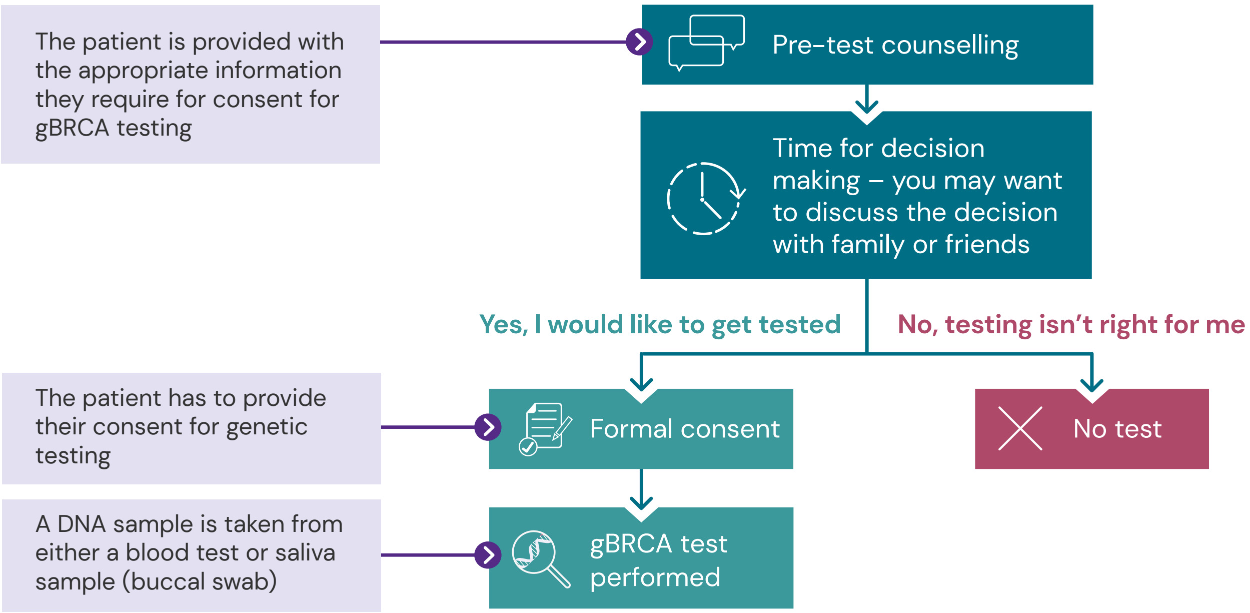

What key points should be discussed with the patient prior to consent to ensure they can make an informed decision?

Before starting a conversation with a patient on genetic testing, it is essential that the patient feels ready to have this conversation at the right time for them. It is important that discussions on genetic testing do not overwhelm the patient or cause them distress, especially if they have only recently received their cancer diagnosis.

It is also useful to understand a patient's motivations for getting a gBRCA test and what their goals of care are.

Ahead of gBRCA testing, it is important that a patient understands what they are providing consent for. Although local guidance may differ and should always be checked, generally the key information a patient should be provided with is:[11–15]

Background to genetics and BRCA mutations

- Some cancers are caused by inherited alterations
- The term hereditary describes a genetic change (or alteration/mutation/variant) which can be passed down/inherited from one generation to the next (or from a parent to a child). This is known as a "germline" alteration[16]
- The most frequently implicated genes for hereditary predisposition to breast cancer are *BRCA1* and *BRCA2*[17]

Relevance of a gBRCAm for their breast cancer

- Based on a population of 253 patients in south of Sweden only a relatively small proportion of people with breast cancer will have a gBRCAm (7%)[18]
- Having a gBRCAm does not mean someone will get cancer, only that their risk of developing certain cancers is greater[19]
- It is important to also highlight that the presence of a gBRCAm is not currently associated with a poor prognosis[19]

How gBRCA testing is conducted

- gBRCA testing is optional
- The patient can take time to make their decision, it does not need to be made on the day of the initial meeting
- A gBRCA test typically involves a blood test and the sample is analysed in a lab

Possible test results

A pathogenic (harmful) gBRCAm is detected and is known to be associated with cancer

↓ **Implications of finding a pathogenic gBRCAm:**

- **Knowledge of gBRCA status can help inform decisions to optimise the management of their cancer; it can help the healthcare team, and the patient, make informed decisions about the type of surgery and treatment options available for their breast cancer**
- Knowing a patient has a gBRCAm also enables future decisions around monitoring and screening, as well as other potential risk-reducing surgeries
- For patients who wish to have children in the future, they can explore reproductive options with their healthcare team
- In addition, as a gBRCAm is inherited, there is a risk that other members of the patient's family may also carry the same alteration
  - Knowledge of gBRCA status can be used to identify other family members at risk of having the mutation
  - It is recommended that family members consider testing for the same genetic alteration as there are options to help reduce their risk of developing cancer

A variant of unknown significance (VUS) is detected
It is also possible that the test result may not provide a definitive answer and a VUS is found (a VUS means that a gBRCA alteration has been detected, but it is unknown whether it is associated with cancer).[20]

If this is the case, the patients may receive further discussions with a genetic counsellor/member of the genetics team

No gBRCAm is detected.
However, the patient may choose to be tested for other genes that are associated with breast cancer

Studies have shown patients prefer to be provided with brief, personalised, positive, and straightforward information without statistics[11–13]

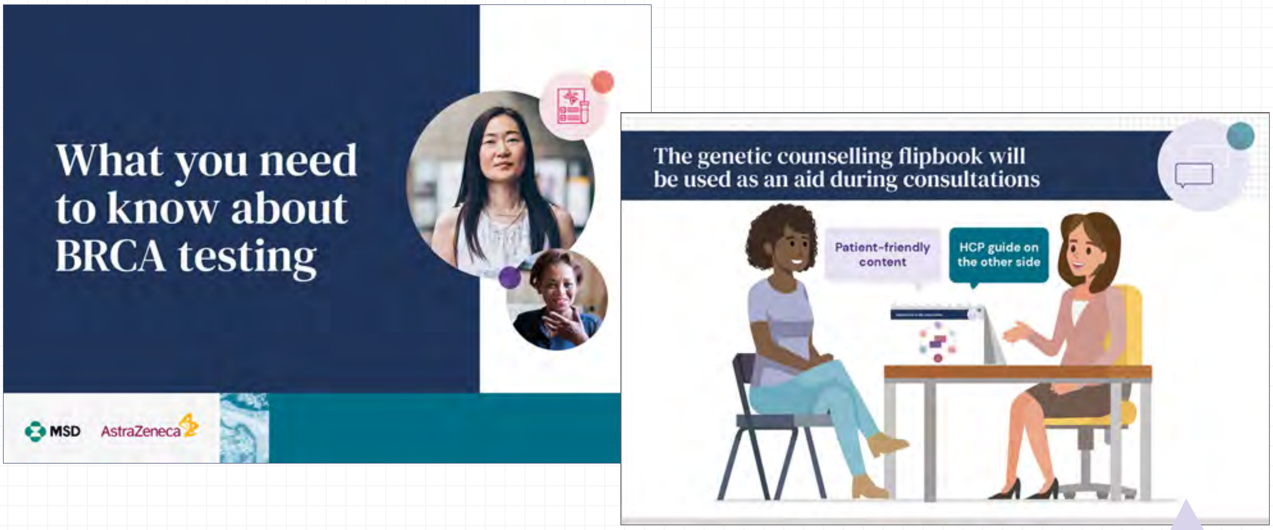

For more information on the key points to discuss with your patients prior to them consenting for BRCA testing please see: *The Genetic Counselling Best Practice Flipbook*

When should a patient be consented for gBRCA testing?

It is important to discuss genetic testing with patients as soon as possible after their breast cancer diagnosis, so they can consent for genetic testing[21–22]

If the patient's gBRCA status is known early, informed decisions can be made to tailor surgery and systemic treatment for the patient and their underlying tumour biology[23–27]

Discussing genetic testing may be difficult for some patients, especially those who have just received their diagnosis, so it is important to be mindful of this and use your personal judgement to assess when it is best to approach genetic testing and consenting conversations with your patients

Studies have shown that pre-test counselling shortly after diagnosis is acceptable for most patients and may reduce cancer-related distress because of the potential impact to optimise decision-making as a result of undergoing genetic testing[11–13, 26]

Nurses can be involved in pre-test counselling of patients to provide them with the appropriate information they require for consent for BRCA testing[2,3]

Why is consenting important?

Genetic consent is a process that ensures the person undergoing testing understands the nature, purpose and potential outcomes from a genetic test.

It is important to understand that genetic testing may have implications for:

The patient

- Test results inform cancer care/treatment decisions
- Test results inform future care including risk reducing measures and screening
- Test results help identify the risk of developing other cancers e.g. ovarian cancer

The patient's family

- If a patient has a gBRCAm then their family members may wish to be tested
- Test results inform family members of any increased risk they may have of developing cancer and guide screening and/or risk reduction strategies

Who can perform genetic consenting?

In addition to genetic counsellors, evidence has shown that genetic consenting may be delivered successfully by any member of the oncology team who has received appropriate training to do so, including oncologists, surgeons and clinical nurse specialists, working together as part of the multidisciplinary team[2]

Medical Oncologist

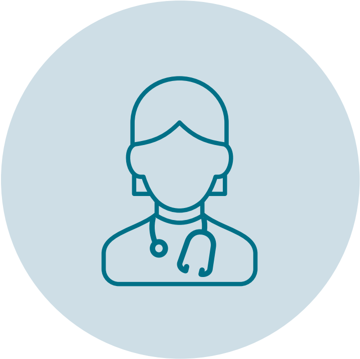

Surgeon

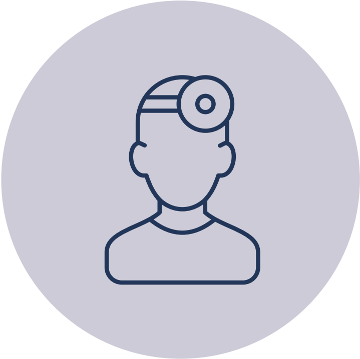

Specialist Nurse

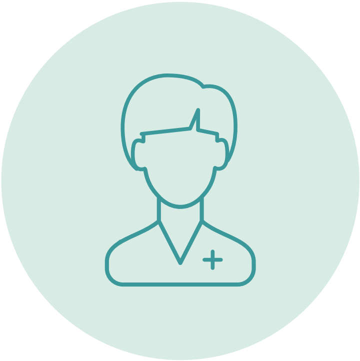

How should evidence of informed patient consent be documented?

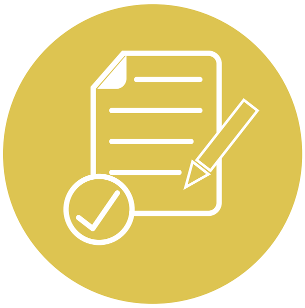

Consent is often integrated into a clinical consultation and should be evidenced by good documentation, either by a signature on a form or a recorded and informed consent discussion.

Consent should therefore be obtained from the patient and documented in a written format (signed) and recorded on the patient's medical notes,[27] in line with the local centre policy.

Useful resources

Please see the following publications for more information on the nurse's role in supporting pre-BRCA test genetic counselling for patients with breast cancer:

- Scheinberg T, et al. Mainstream consent programs for genetic counseling in cancer patients: A systematic review. *Asia-Pacific J Clin Oncol*. 2021;17(3):163–177
- Steven J & Donnelly J. Breast care nurses: a pivotal role in genetic testing in the breast clinic – an update. *Eur J Surg Oncol*. 2020;46(6):e9
- Scott N, et al. Changing practice: moving to a specialist nurse-led service for BRCA gene testing. *Br J Nurs*. 2020;29(10):S6-S13
- Kemp Z, et al. Evaluation of Cancer-Based Criteria for Use in Mainstream BRCA1 and BRCA2 Genetic Testing in Patients With Breast Cancer. *JAMA Network Open*. 2019;2(5):e194428
- Jacobs C, et al. Communication about genetic testing with breast and ovarian cancer patients: a scoping review. *Eur J Hum Genet*. 2019;27(4):511–524

For more information on pre-BRCA test genetic counselling, please see the following:

https://coursesandconferences.wellcomeconnectingscience.org/news_item/free-online-course-to-help-nurses-with-genetic-counselling-in-the-new-genomic-era/

https://www.genomicseducation.hee.nhs.uk/news/new-genomics-and-counselling-skills-course/

https://www.facingourrisk.org/webinars

https://mcgprogramme.com/brcatoolkit

https://www.idbrca.com/breast-home.html

https://www.idbrca.com/breast-home/streamlining-genetic-counselling.html

All resources live as of October 2022

Abbreviations

BRCAm=BRCA mutation; gBRCA=germline BRCA; gBRCAm=germline BRCA mutation; HCP=healthcare professional; VUS=variant of unknown significance.

References

1. Scheinberg T, et al. *Asia Pac J Clin Oncol*. 2021;17:163–177
2. Percival N, et al. *Br J Nurs*. 2016;25:690–694
3. George A, et al. *Sci Rep*. 2016;6:29506
4. Berger-Höger B, et al. *Int J Nurs Stud*. 2019;9:141–152
5. Scott N, et al. *Br J Nurs*. 2020;29:S6–S13
6. Isselhard A, et al. *Trials*. 2020;21:501
7. Berger-Höger B, et al. *BMC Nurs*. 2022;21:42
8. Kemp Z, et al. *JAMA Netw Open*. 2019;2:e194428
9. Visser A, et al. *Clin Nurse Spec*. 2015;29:E1–7
10. Bains S, et al. *Eur J Surg Oncol*. 2018;44:888
11. Jacobs C, et al. *Eur J Hum Genet*. 2019;27:511–524
12. Gleeson M, et al. *Oncol Nurs Forum*. 2013;40:275–283
13. Meiser B, et al. *Oncol Nurs Forum*. 2012;39:E101–111
14. Metcalfe KA, et al. *J Med Genet*. 2000;37:866–874
15. Lobb EA, et al. *Br J Cancer*. 2004;90:321–327
16. National Cancer Institute. Germline mutation. Available at: https://www.cancer.gov/publications/dictionaries/cancer-terms/def/germline-mutation Accessed October 2022
17. Manahan E, et al. *Ann Surg Oncol*. 2019;26:3025–3031
18. Winter C, et al. *Ann Oncol*. 2016;27:1532–1538
19. Tung NM, et al. J Clin Oncol. 2020;38(18):2080-2106
20. National Cancer Institute. Variant of unknown significance. Available at: https://www.cancer.gov/publications/dictionaries/cancerterms/def/variant-of-unknown-significance Accessed October 2022
21. Cardoso F, et al. *Ann Oncol*. 2018;29:1634–1657
22. Yadav S, et al. *Hered Cancer Clin Pract*. 2017;15:11
23. Robson M, et al. *N Engl J Med*. 2017;377:523–533
24. Diéras V, et al. *Lancet Oncol*. 2020;21:1269–1282
25. National Cancer Institute. BRCA Mutations: Cancer Risk and Genetic Testing. Available at: https://www.cancer.gov/about-cancer/causes-prevention/genetics/brca-fact-sheet Accessed October 2022
26. Christie J, et al. *Ann Surg Oncol*. 2012;19:4003–4011
27. The Royal College of Pathologists. Consent and confidentiality in genomic medicine. Available at: https://www.rcplondon.ac.uk/projects/outputs/consent-and-confidentiality-genomic-medicine Accessed October 2022

What you need to know about BRCA testing

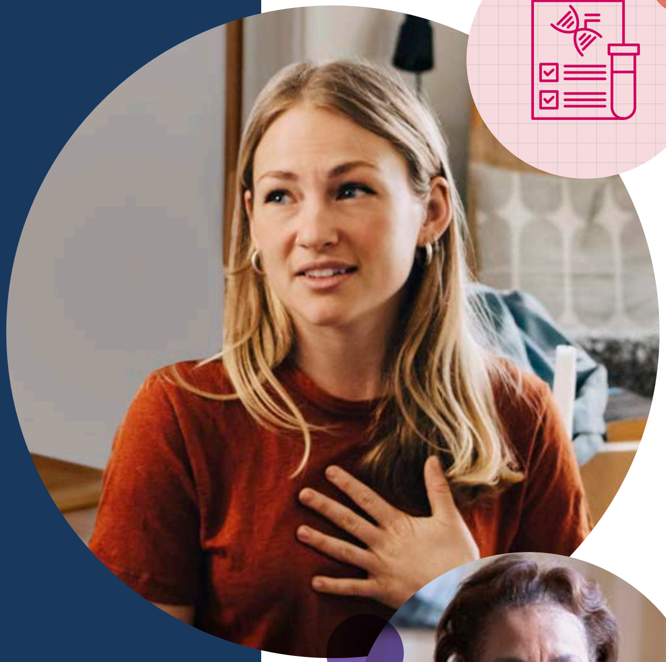

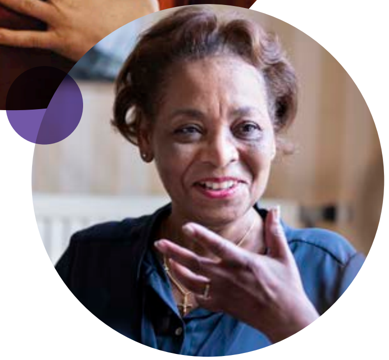

This booklet has been co-developed with healthcare professionals and patients to provide you with an overview of the importance of genetics and genetic testing in breast cancer. This Patient educational booklet is not intended to replace the advice of your Doctor. Please always speak to your doctor, nurse or pharmacist for further information.

Z4-48226 | November 2022

This genetic counselling flipbook will be used as an aid during consultations

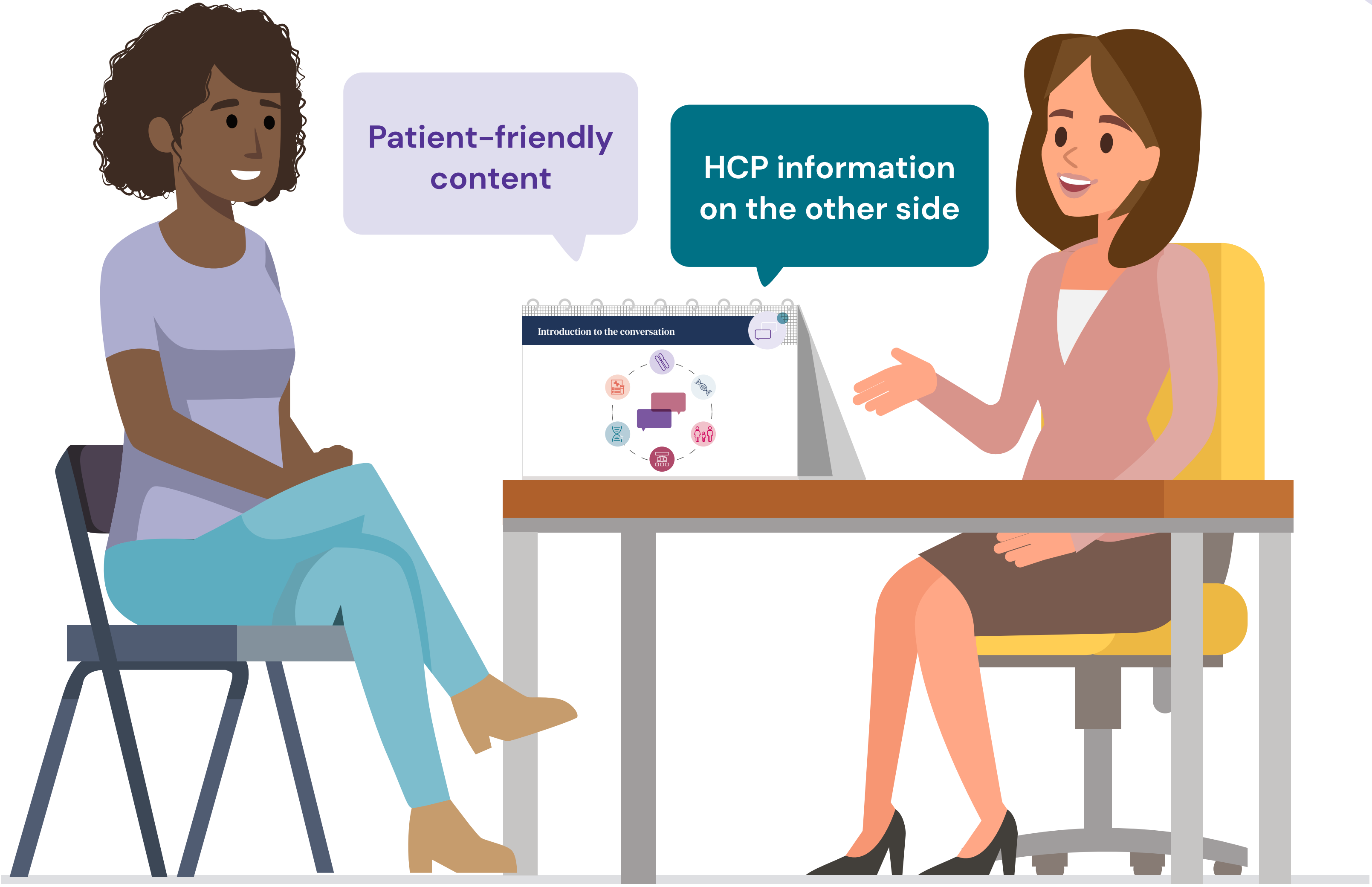

This genetic counselling flipbook will be used as an aid during consultations

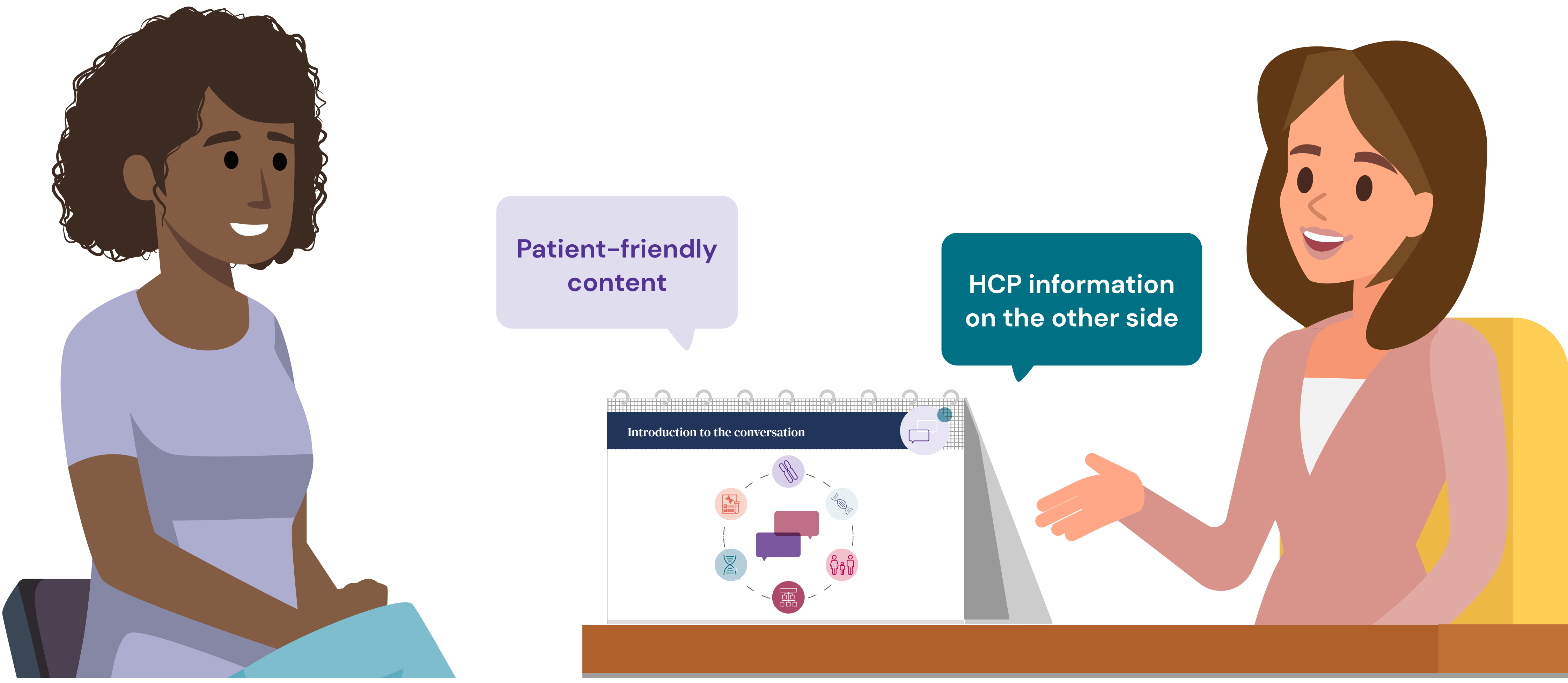

Introduction to the conversation

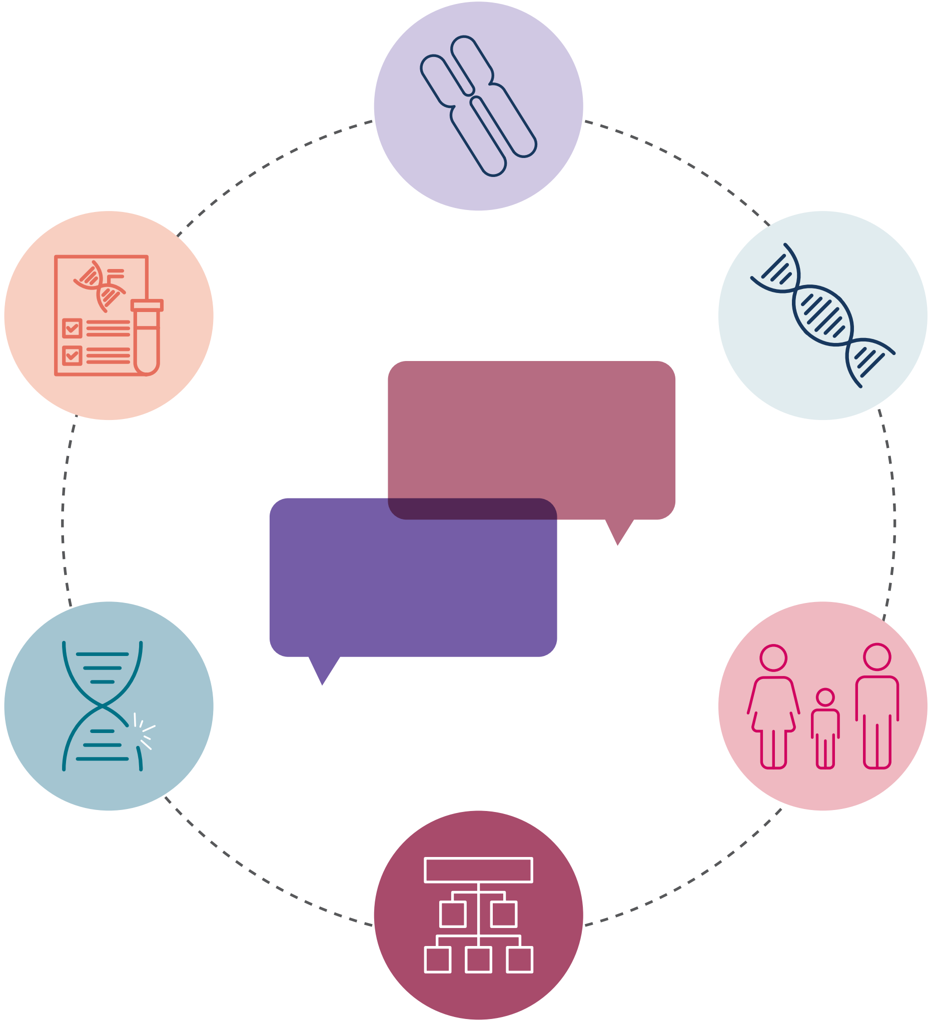

Introduction to the conversation

Before you start this conversation, it is important to communicate the following points:

- Germline BRCA (gBRCA) testing is **optional**
- A relatively small proportion (3–4%) of people with breast cancer harbour a gBRCA alteration
- Presence of a gBRCA alteration is **not associated with a poor prognosis**
- Knowledge of gBRCA status can help inform decisions to **optimise their cancer treatment**
- If a gBRCA alteration is detected, there are options for potentially affected **family members**

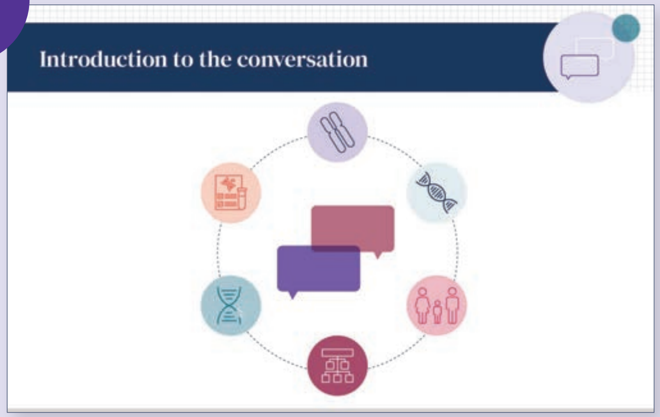

A visualisation of what your patient will see will be included on each of your pages

Patient pages are further identified by a decorative banner and HCP pages have a plain navy banner

Before you start, consider asking the patient what they would like to understand from this conversation and if they have any questions

Tung NM, et al. J Clin Oncol. 2020;38(18):2080–2106.

What can you expect from this conversation?

By the end of this conversation, you should have greater understanding of:

- The BRCA genes (BRCA1/BRCA2)
- Why the BRCA genes may be important in your breast cancer treatment
- How a germline BRCA (gBRCA) test is done
- The reasons why you might want to consider having a gBRCA test

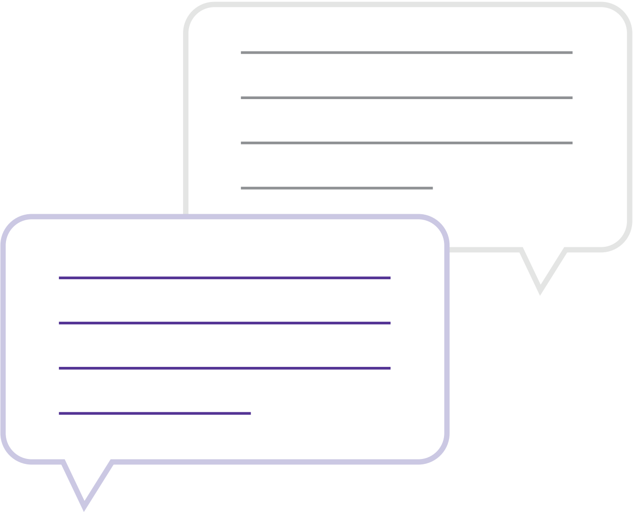

What is included in this support tool for pre-test counselling of patients with breast cancer?

This tool should help you discuss with your patient:

- What the BRCA genes are
- Why the BRCA genes may be important for their breast cancer treatment and decision making
- How a gBRCA test is done
- Why they might want to consider having a gBRCA test

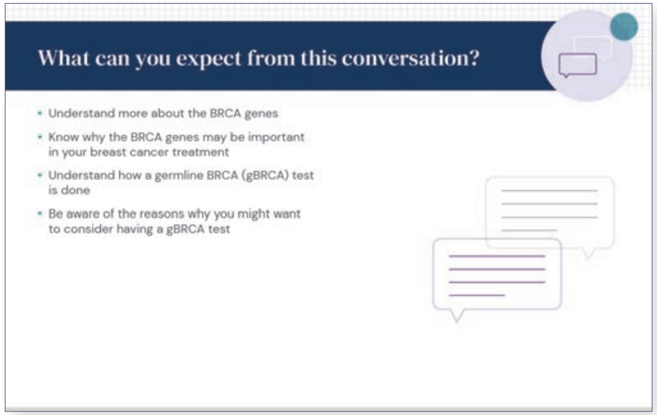

About the BRCA alteration

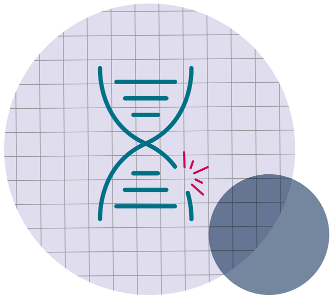

About the BRCA alteration

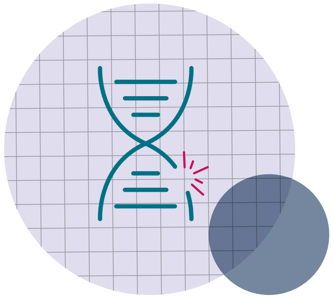

What is a gene?

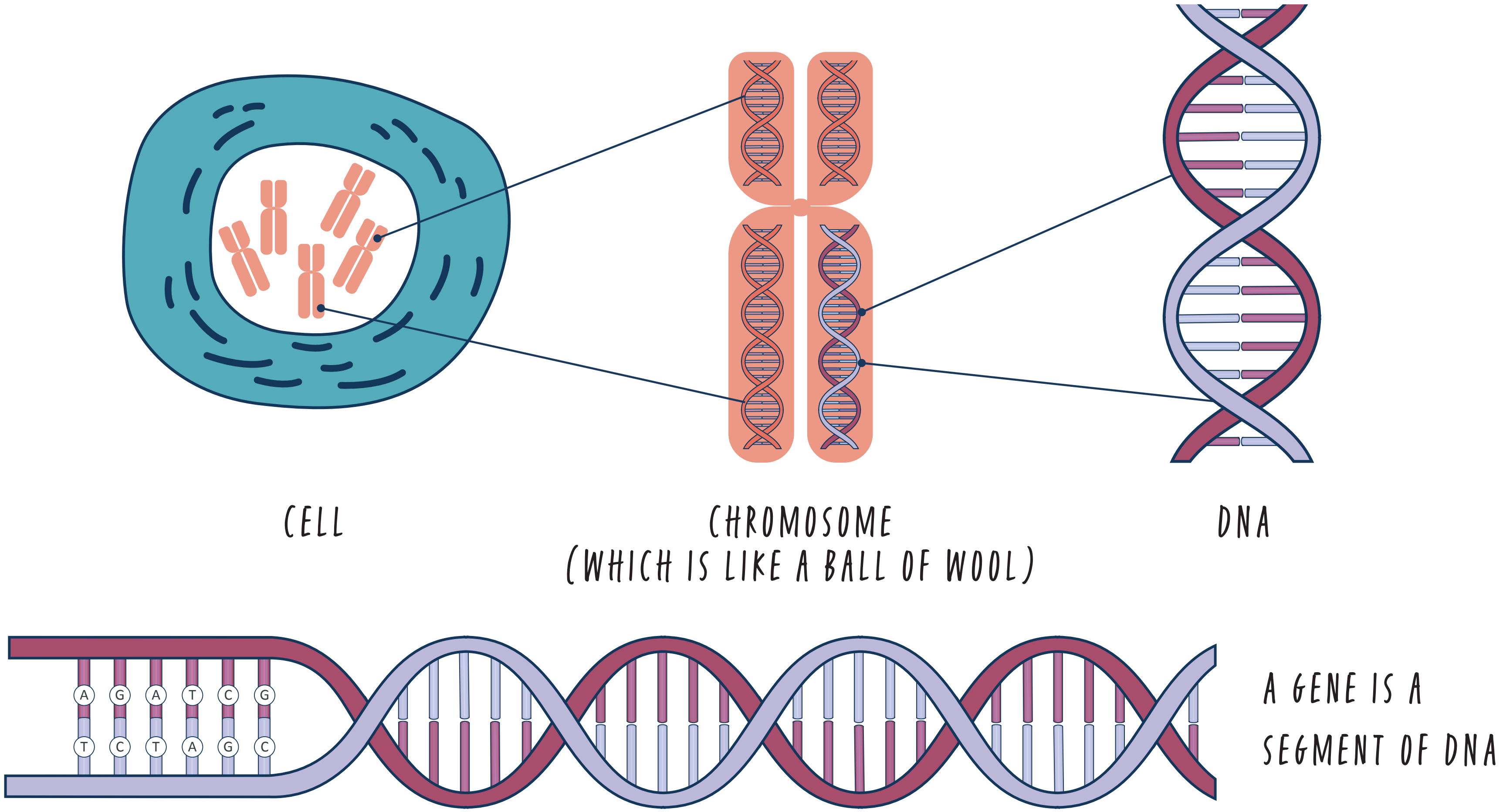

What is a gene?

You may want to discuss the following points:

- The body is made up of cells. Almost every cell in the body contains chromosomes which are made from DNA, the building blocks of life
- A gene is a segment of DNA which contains the instructions for making a specific protein

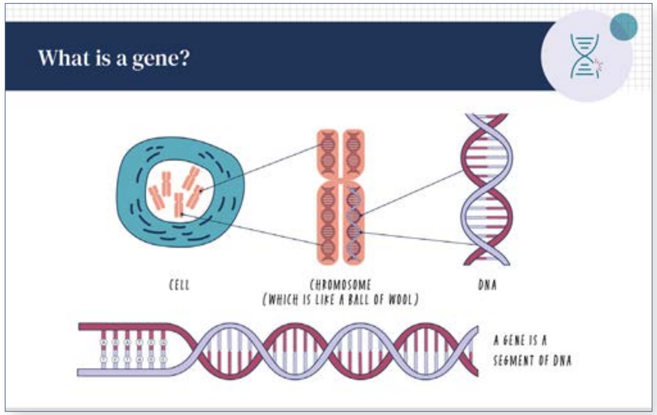

Why are genes important?

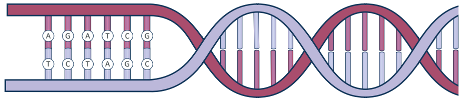

GENE

=

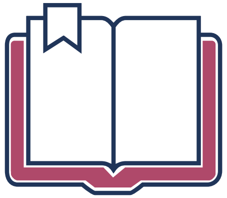

RECIPE

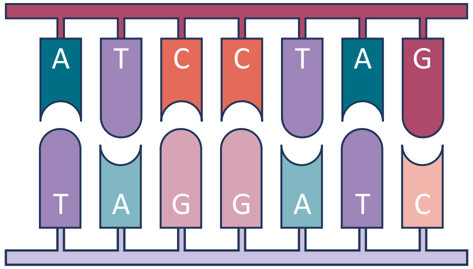

NUCLEOTIDES

=

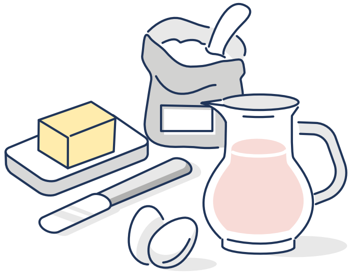

INGREDIENTS

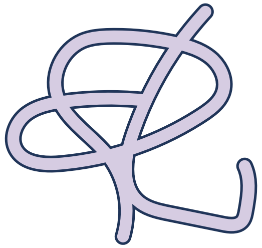

PROTEIN

=

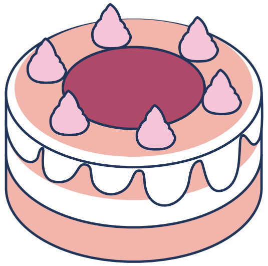

CAKE

Why are genes important?

This analogy can be used to support the patient's understanding of what genes and proteins are and why they are important

You may want to discuss the following points:

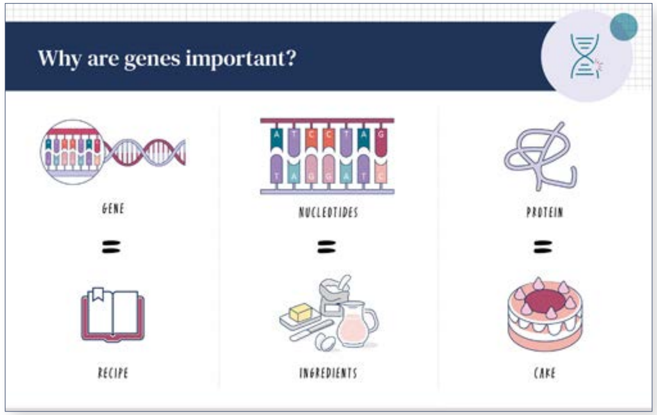

- Cells need proteins to help them work correctly
- We can think of genes as the instructions, or recipe, for cells to make a protein
- Each gene in the body is made up from a set of nucleotides, or 'ingredients'
- When genes are working correctly, they will make proteins which help cells to work correctly
- This is like making a cake, with the correct recipe and ingredients a good cake is made

What happens if a gene doesn't work correctly?

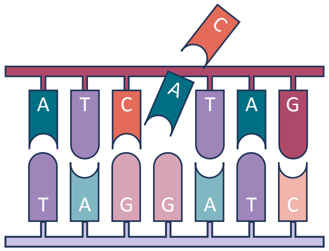

GENETIC CHANGE / ALTERATION IN GENE

=

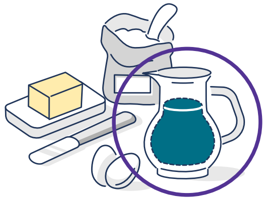

WRONG INGREDIENT

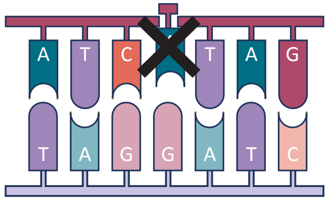

GENE IS MISSING

=

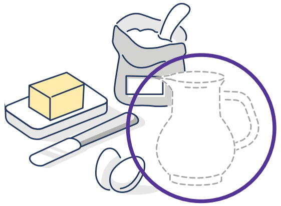

MISSING INGREDIENT

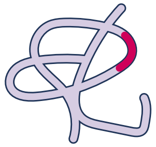

INCORRECT FUNCTIONING OF PROTEIN

=

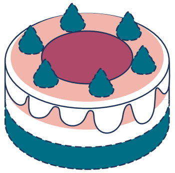

WRONG FLAVOUR CAKE

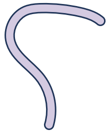

INCORRECT FUNCTIONING OF PROTEIN

=

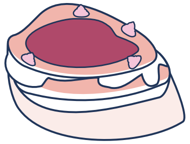

WRONG FLAVOUR CAKE

What happens if a gene doesn’t work correctly?

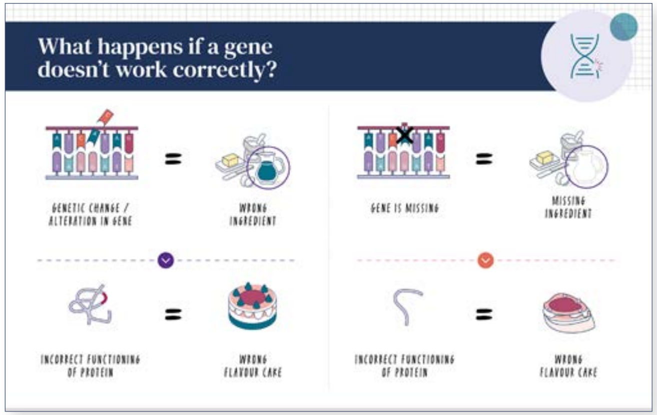

Explanation of analogy

You may want to discuss the following points:

- A genetic alteration is an error in a person’s DNA. This is sometimes referred to as a genetic mutation or a genetic variant
- Whilst most gene alterations are harmless, some are associated with an increased risk of cancer
- The BRCA genes are involved in the repair of damaged DNA within a cell
- Harmful alterations in the BRCA genes can lead to incorrect DNA repair which can result in the development of cancer
- In the examples here, if the ingredients to make a cake are wrong or missing there will be something wrong with the cake

The BRCA genes and breast cancer

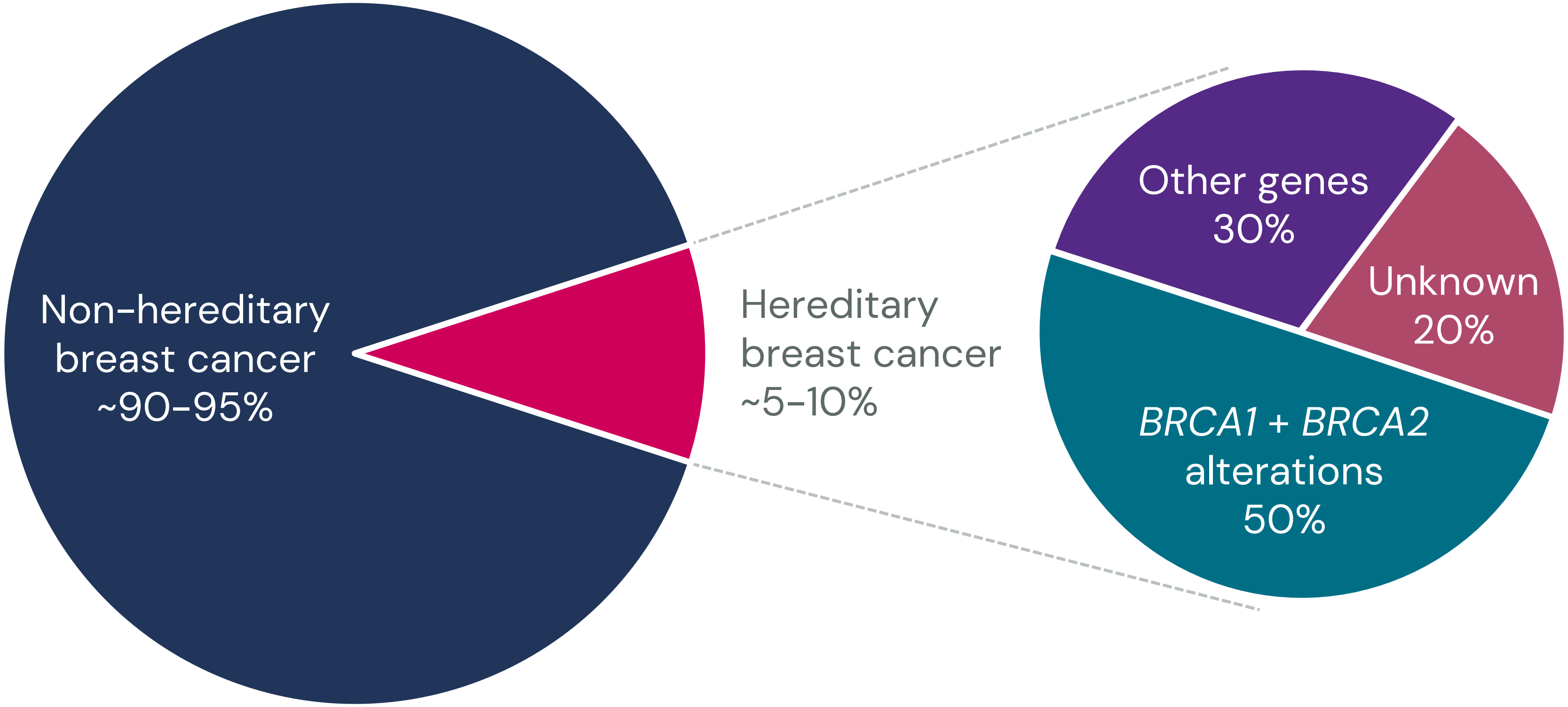

Some cancers are caused by inherited alterations, the most commonly associated genes with hereditary breast cancer are *BRCA1* and *BRCA2*

American Cancer Society. Available at: https://www.cancer.org/cancer/breast-cancer/risk-and-prevention/breast-cancer-risk-factors-you-cannot-change.html (Accessed September 2022).

The BRCA genes and breast cancer

You may want to discuss the following points:

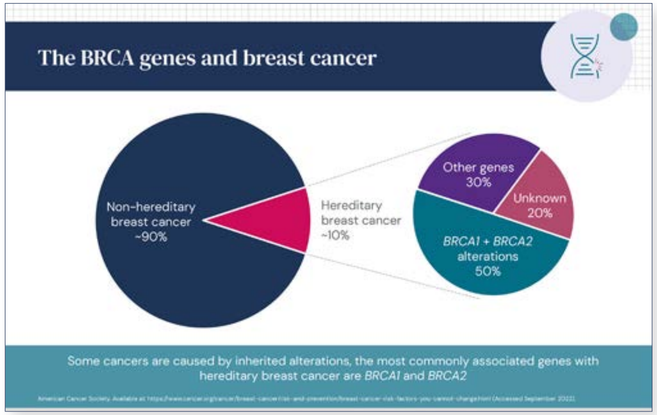

- Some types of breast cancer can be strongly influenced by genes and can run in families
- Out of all breast cancer cases, approximately 5–10% are hereditary
- The term 'hereditary' describes a genetic change (alteration/ mutation/variant) which can be passed down / inherited from one generation to the next (from a parent to a child). This is known as a 'germline' alteration
- Within hereditary breast cancers, around 50% are caused by alterations in the *BRCA1* and/or *BRCA2* genes
- There are also other genes associated with hereditary breast cancer, but these are not fully understood
- There are certain features that may suggest a patient is likely to have hereditary breast cancer – if the patient is younger, has a family history of cancer and / or has triple-negative breast cancer

?

You may want to ask your patient if they have heard of the BRCA gene

American Cancer Society. Available at: https://www.cancer.org/cancer/breast-cancer/risk-and-prevention/breast-cancer-risk-factors-you-cannot-change.html (Accessed September 2022).
Buys SS, et al. Cancer. 2017;123(10):1721-1730.

A gBRCA alteration may be passed down from a parent to their child

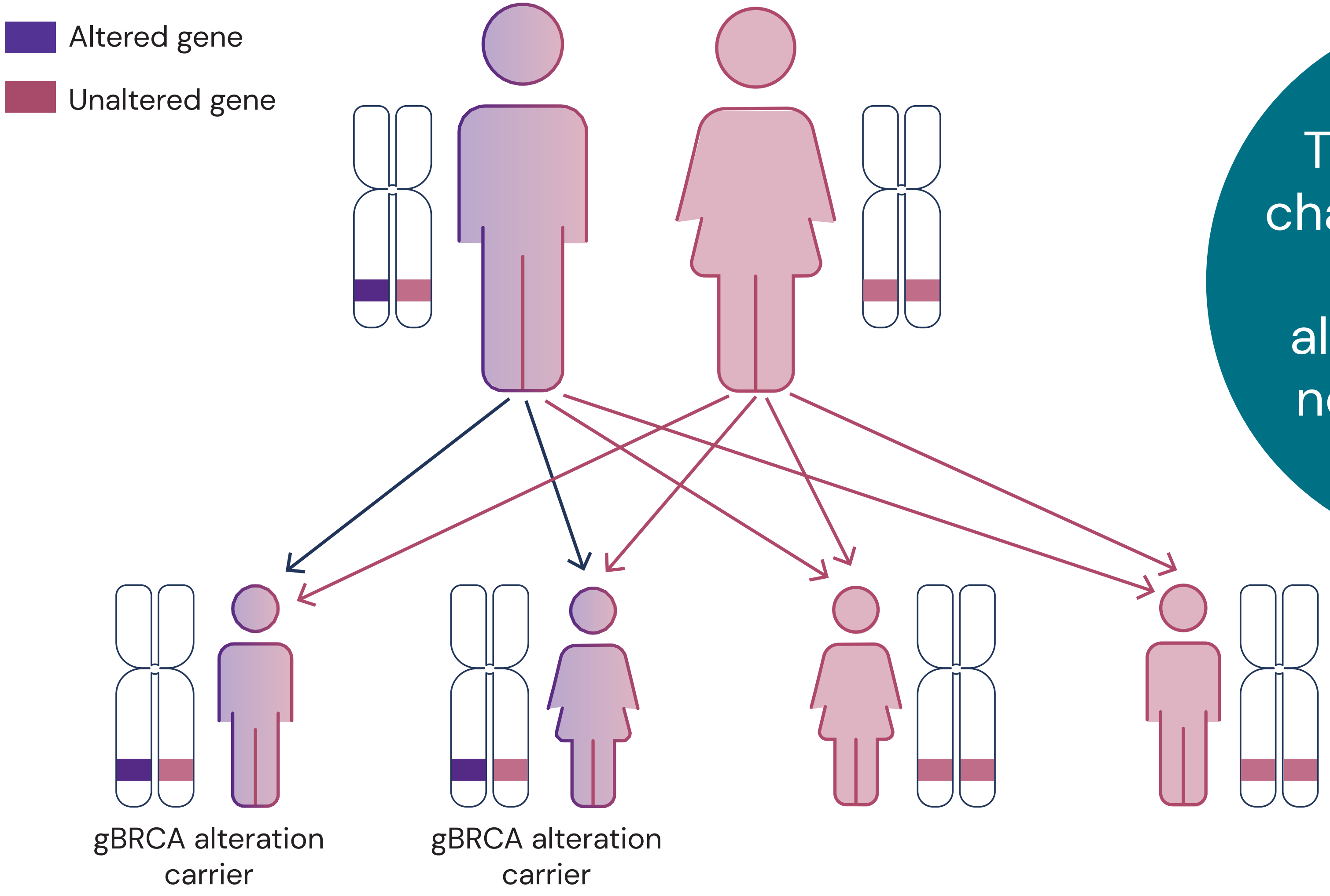

There is a **50%** chance of passing on a gBRCA alteration to the next generation

A gBRCA alteration may be passed down from a parent to their child

You may want to discuss the following points:

- Germline alterations in BRCA genes are inherited and can be passed down from either the maternal (mother) **OR** paternal (father) line
- If a patient harbours a gBRCA alteration there is a 50% chance of passing on the same alteration to each of their children. This can affect both male and female children
- This is why a detailed family history is taken for all types of cancer when the patient is diagnosed with breast cancer

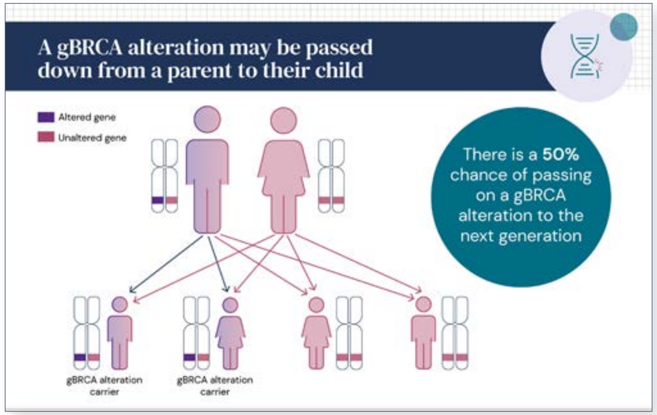

?

It is important to ask the patient if they are aware of any family history of cancer

The two-hit hypothesis

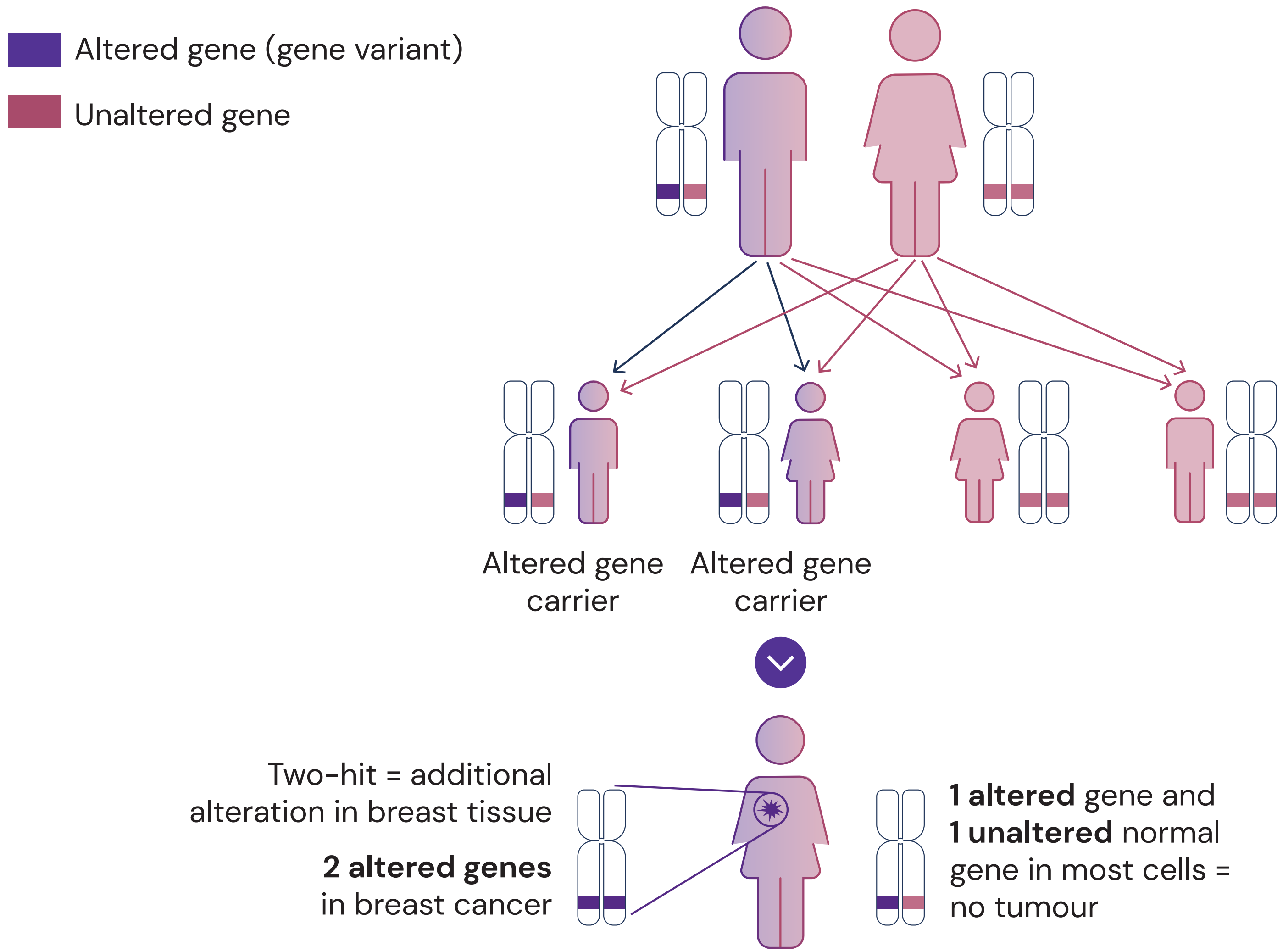

The two-hit hypothesis (back-up page)

Please use this page reactively if your patient has expressed an interest in this topic and wishes to understand the two-hit hypothesis in more detail

You may want to discuss the following points:

- In hereditary cancer, one damaged or altered gene is inherited from one parent which is described as the 'first-hit'

- If a subsequent change occurs in a specific cell type (e.g. breast tissue), this results in a second damaged/ mutated gene, which can lead to cancer. This is described as the 'second-hit'

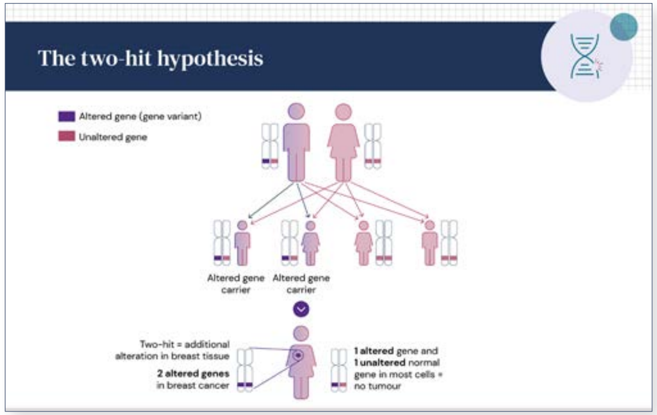

What is the lifetime risk of cancer in people with a gBRCA alteration?

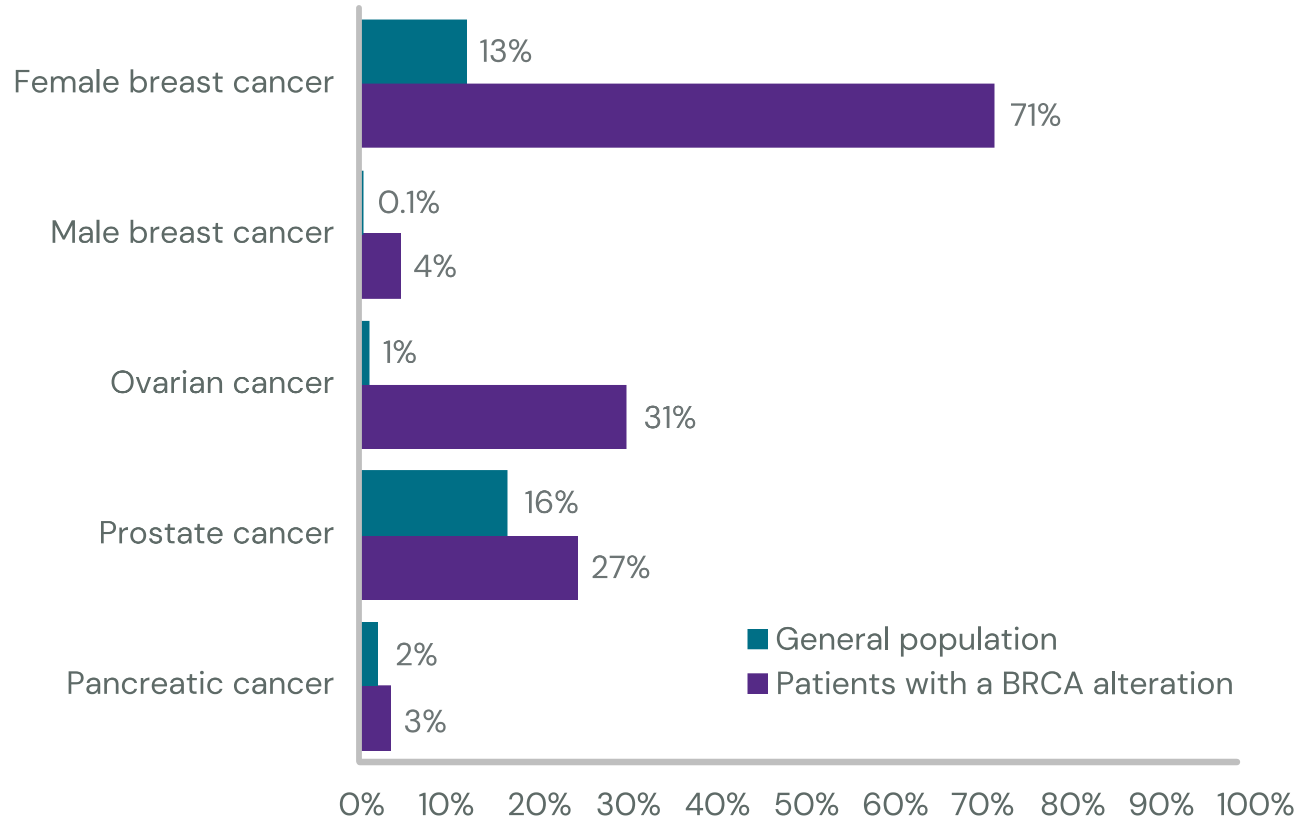

If a gBRCA alteration is found, action can be taken to screen for early detection and potentially reduce the risk of cancer developing

Some of these statistics are based on data from Switzerland

Bundesamt für Statistik. Schweizerischer Krebsbericht 2015 Stand und Entwicklungen; Kuchenbaecker KB et al. *JAMA*. 2017;317(23):2402–2416; Ibrahim M et al. *BMC Cancer*. 2018;18(1):179 Li et al. JCO 2022 40:14, 1529–1541.

What is the lifetime risk of cancer in people with a gBRCA alteration?

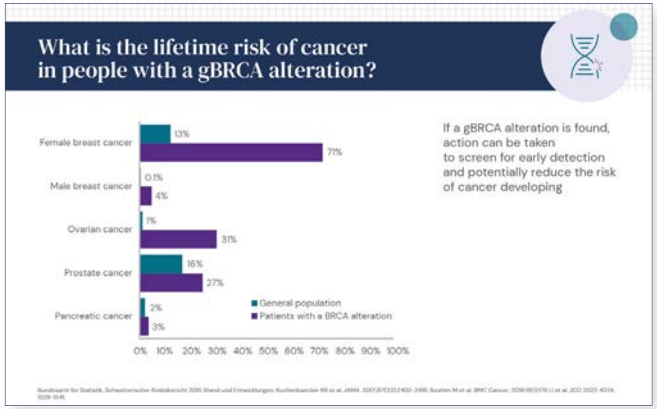

You may want to discuss the following points:

- A gBRCA alteration may have been the cause of the patient's current cancer, but it also increases the risk of future cancers
- People with a gBRCA alteration are at increased risk of developing breast cancer (regardless of their sex), ovarian cancer, prostate cancer and pancreatic cancer, compared with the general population
- The risk of developing cancer is not the same over time, as the risk increases with age. The information presented in this graph represents the lifetime risk of developing these cancers. The actual risk will vary from patient to patient and will change as they get older
- The lifetime risk of developing ovarian cancer is higher in people with a *BRCA1* alteration, whilst the lifetime risk of prostate and pancreatic cancer is higher in people with a *BRCA2* alteration
- Close monitoring and screening, as well as risk-reducing surgeries, may be adopted for family members who have a harmful gBRCA alteration

Kuchenbaecker KB et al, *JAMA* 2017;317:2402-2416; National Cancer Institute. Pancreatic Cancer. Available at: http://seer.cancer.gov/statfacts/html/pancreas.html (Accessed September 2022).

The benefits of BRCA testing for you and your family

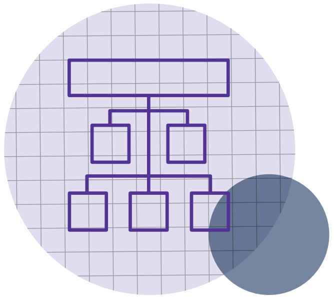

The benefits of BRCA testing for you and your family

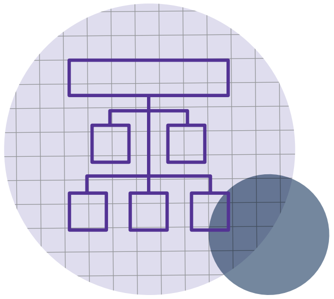

Why should you consider getting tested for a gBRCA alteration?

Inform cancer care / treatment options

Inform future care beyond this cancer

Help your family

Why should a patient consider getting tested for a gBRCA alteration?

You may want to provide a broad overview of the impact of a patient knowing their gBRCA status before going into more detail on each point

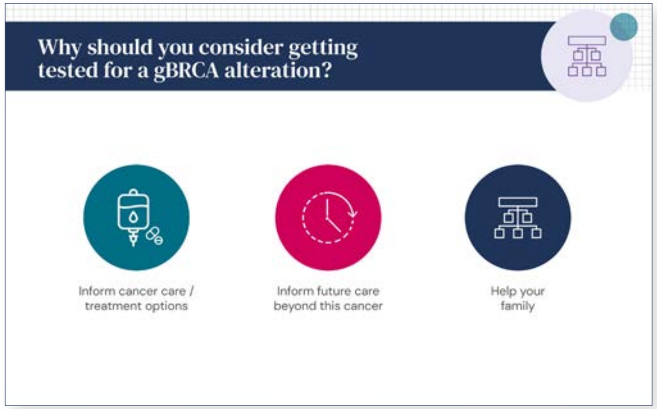

You may want to discuss the following points:

- It can help with early decisions on how the patient's cancer is managed, including what the preferred surgery or treatment might be
- It can also help inform future care beyond this cancer, and if they have any risks of developing other cancers
- It can help inform the patient's family of any increased risk they might have of developing cancer
- However, some people prefer not to know their gBRCA status and testing is optional; at the patient's discretion

Why should you consider getting tested for a gBRCA alteration?

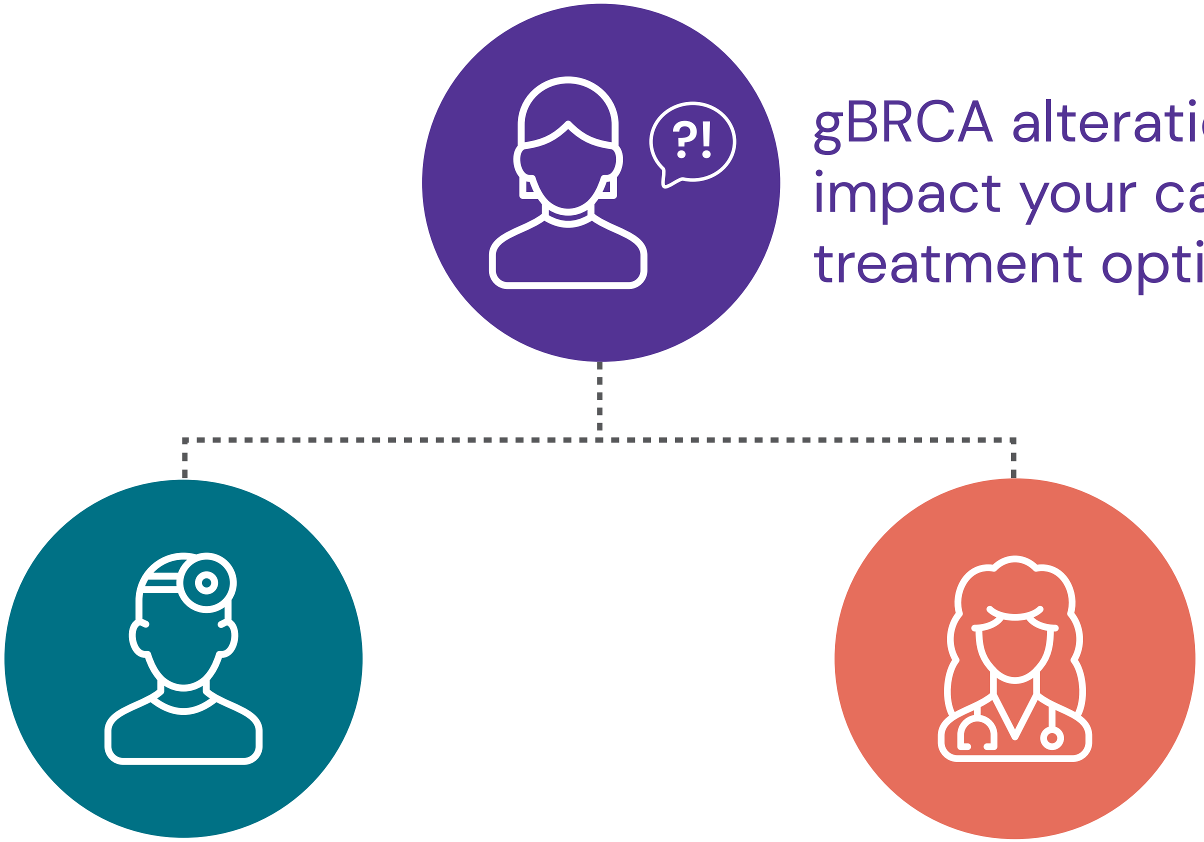

gBRCA alterations may impact your cancer care / treatment options

Surgical decisions

Medical therapy decisions

Why should a patient consider getting tested for a gBRCA alteration?

You may want to discuss the following points:

- Knowing the patient has a gBRCA alteration may change early decisions in their care
- For example, it may impact the decision around what type of surgery is recommended and help the patient decide the surgery they want
- It may also inform other treatment choices, for example their chemotherapy options or targeted treatment

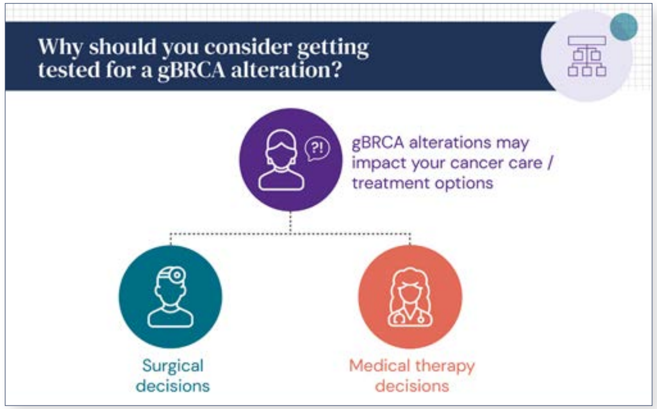

Potential question to ask:
Do you have questions about any elements of your care plan?

Why should you consider getting tested for a gBRCA alteration?

gBRCA alterations are associated with an increased risk of other cancers, including ovarian cancer

Actions can be taken to reduce the risk of future cancers

Why should a patient consider getting tested for a gBRCA alteration?

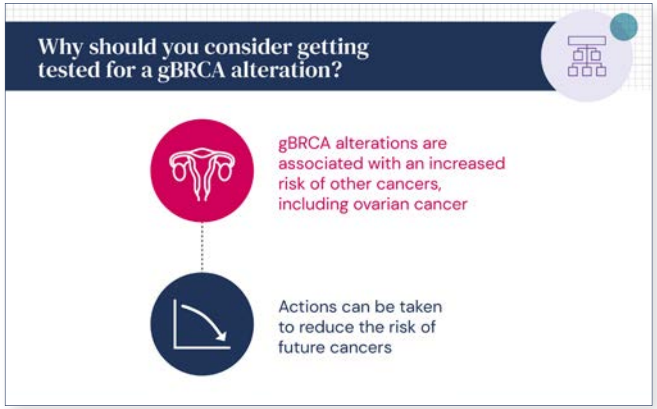

You may want to discuss the following points:

- gBRCA alteration carriers are known to be at risk of second malignancies (or new cancers)
- One of the most common secondary malignancies / cancers is ovarian cancer
- Risk-reducing surgeries, such as salpingo-oophorectomy, may be considered for some women
- Screening may also be offered to patients with gBRCA alterations for early detection of any future cancers

?

Potential question to ask:
Do you have any questions about risk-reducing measures/actions?

Why should you consider getting tested for a gBRCA alteration?

If you have a gBRCA alteration, some of your family members may also have the same alteration

Family members may also choose to be tested

Why should a patient consider getting tested for a gBRCA alteration?

You may want to discuss the following points:

- If a patient has a gBRCA alteration, then the same gene alteration may exist in their family members
- Testing wider family members is important to identify additional gBRCA alteration carriers so they can take actions to reduce their risk of future cancers, if they wish to

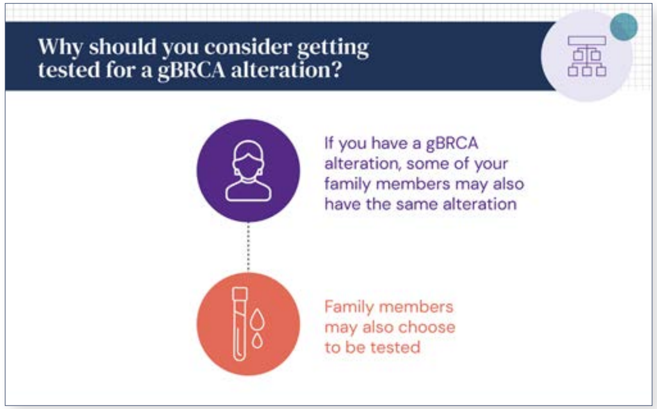

How gBRCA testing works and what the results mean

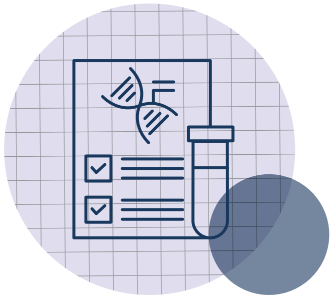

How gBRCA testing works and what the results mean

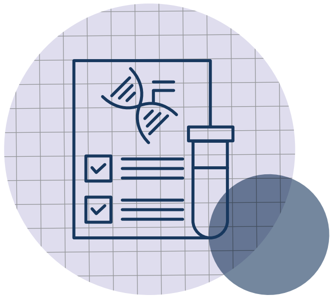

What decision is right for me?

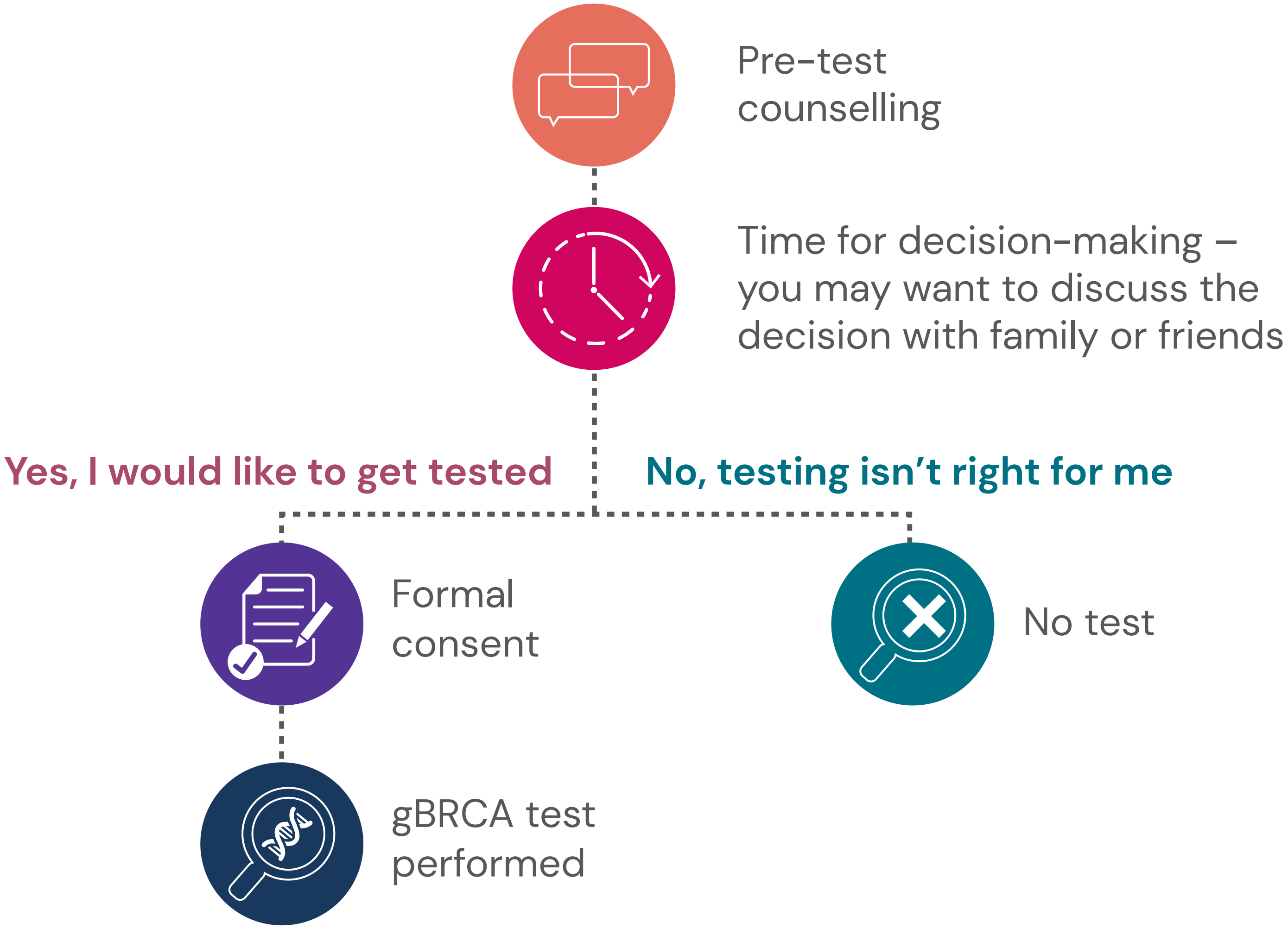

What decision is right for the patient?

You may want to discuss the following points:

- gBRCA testing is not right for everyone and it should be an individualised decision
- It is important to understand the implications of gBRCA testing before agreeing to be tested
- This decision does not need to be made immediately and the patient has the option to discuss the decision with family or friends
- It is normal to experience some anxiety about test results – weighing up the pros and cons of testing is important to be ready for what the results will mean for the patient and their family
- Additionally, the patient may be at increased risk of other cancers, even if no gBRCA alteration is detected, if they have a significant family history

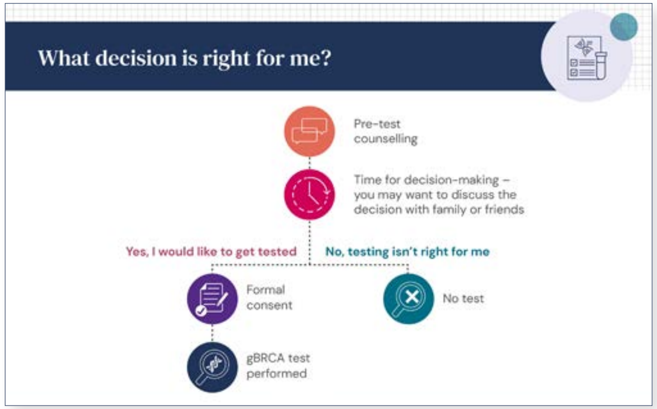

What processes are involved in testing for a gBRCA alteration?

- Consent for testing
- Sample taken
- Sample tested
- Results received

What processes are involved in testing for a gBRCA alteration?

You may want to discuss the following points:

- The patient should understand that gBRCA testing cannot take place without their formal consent (or agreement)
- It can take some time for the results of the gBRCA test to be returned
- When the results are returned, an expert will discuss the results with the patient and provide any required psycho-social (or counselling) support

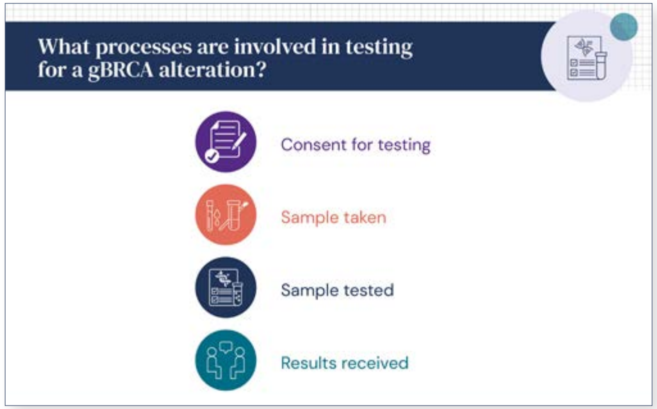

?

Potential question to ask:
Are any parts of this process unclear to you?

Gene panel tests detect alterations in other genes in addition to *BRCA1* & *BRCA2*

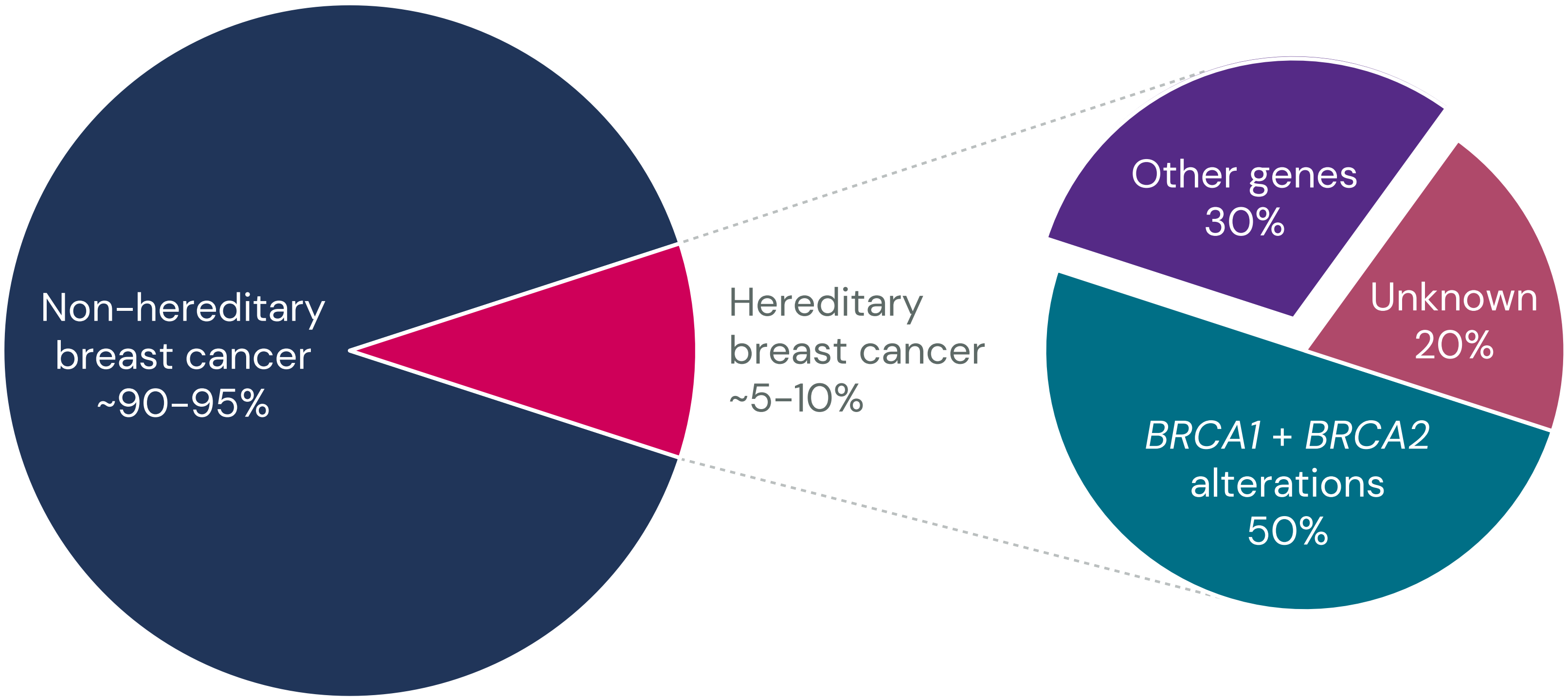

Gene panel tests look for alterations in the BRCA genes, but also look for alterations in a number of other genes that are associated with breast cancer

Gene panel tests detect alterations in other genes in addition to *BRCA1* & *BRCA2*

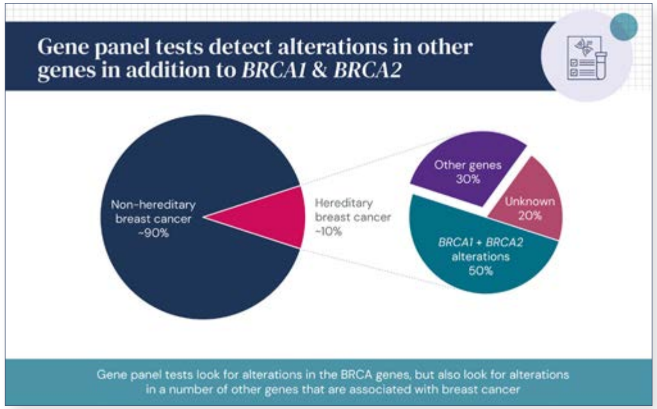

Please only use this page if gene panel testing is being offered to the patient

You may want to discuss the following points:

- Alterations in *BRCA1* and *BRCA2* account for around 50% of inherited gene alterations which are associated with an increased risk of cancer
- Alterations in genes other than *BRCA1* and *BRCA2* are also associated with increased risk of breast cancer
  - There may be some variations but the panel may include: *PALB2, TP53, STK11, PTEN, CHEK2, CDH1, RAD51C, RAD51D* and *ATM*
  - A larger panel may be indicated if there are other types of cancer in the family
- Gene panel tests detect alterations in a number of genes simultaneously
- If an alteration in a gene other than BRCA is detected, more information will be provided to the patient to understand what that result means for them

American Cancer Society. Available at: https://www.cancer.org/cancer/breast-cancer/risk-and-prevention/breast-cancer-risk-factors-you-cannot-change.html (Accessed September 2022).

What happens when the results come back?

A harmful alteration is found

Family members should consider testing

You will receive further counselling to better understand your options

An alteration with uncertain meaning is found*

Family members do not need to be tested

No genetic alteration is found

Family members do not need to be tested

Your care plan will be tailored based on your results. Results may be used to inform surgical and medical therapy decisions

*This is sometimes called a VUS or variant of unknown significance

What happens when the results come back?

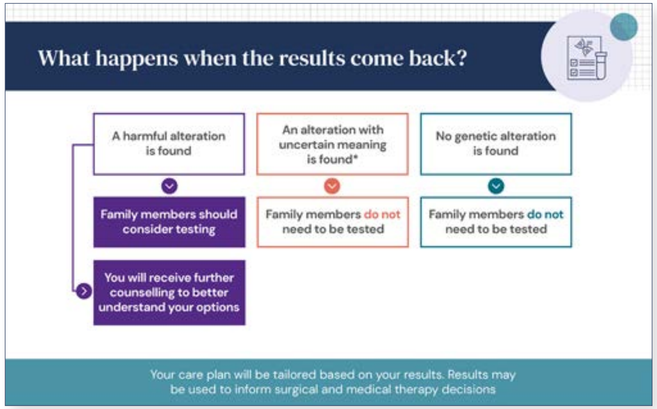

You may want to discuss the following points:

- When the patient's DNA sample is assessed for gBRCA alterations, the results may come back as either:
  - **A pathogenic mutation / alteration / variant is found**. This means that a gBRCA alteration has been found and it is known to be associated with cancer
  - **A variant of unknown significance (VUS) is found.** This means that a gBRCA alteration has been found but it is not known whether it is associated with cancer. A VUS may be re-evaluated at some point in the future
  - **No mutation / alteration is found**
- If a pathogenic (harmful) alteration is found, the patient's care plan may be impacted, including surgical and systemic treatment options
- It is also recommended that family members should consider testing for the same genetic alteration
- In the case of a VUS or no alteration is detected, family members do not require gBRCA testing but if there is a strong family history of cancer, further discussions around future testing may be needed

?

Potential question to ask:
Do you have any questions about these different possible results?

What might gBRCA testing mean for me?

There are several reasons why you may consider being tested. Knowing if you have a gBRCA alteration can help you and your doctor to decide your next steps, including:

- **Treatment options**
- **Choice of surgery**
- **Measures to reduce the risk of further cancer**
- **Screening for potential future cancers**
- **Testing family members**
- **Family planning**
- **Medical insurance**

It is important that you have all the information you need and understand all the implications of testing before you make the decision to be tested

What might gBRCA testing mean for the patient?

You may want to discuss the following points:

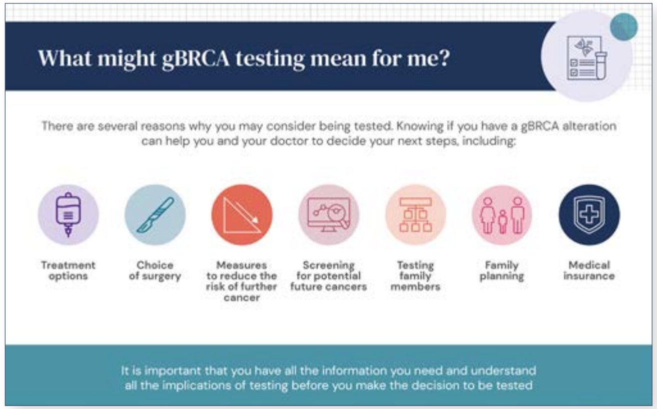

Treatment options
When the time comes for systemic treatment, the patient may be suitable for different treatment options, such as targeted therapy

Choice of surgery
Surgery options will be discussed with the patient so they can choose the right option for them

Measures to reduce the risk of further cancer
Risk reducing measures, including additional surgeries, may be offered to the patient to reduce their risk of secondary cancers

Screening for potential future cancers
Screening for potential future cancers is available for patients to ensure early detection if a second primary cancer develops

Testing family members
The patient's family may also choose to be tested for a gBRCA alteration

Family planning
The patient may wish to explore their family planning options

Medical insurance
You may also want to discuss the implications (if any) of gBRCA testing on medical insurance

Further information

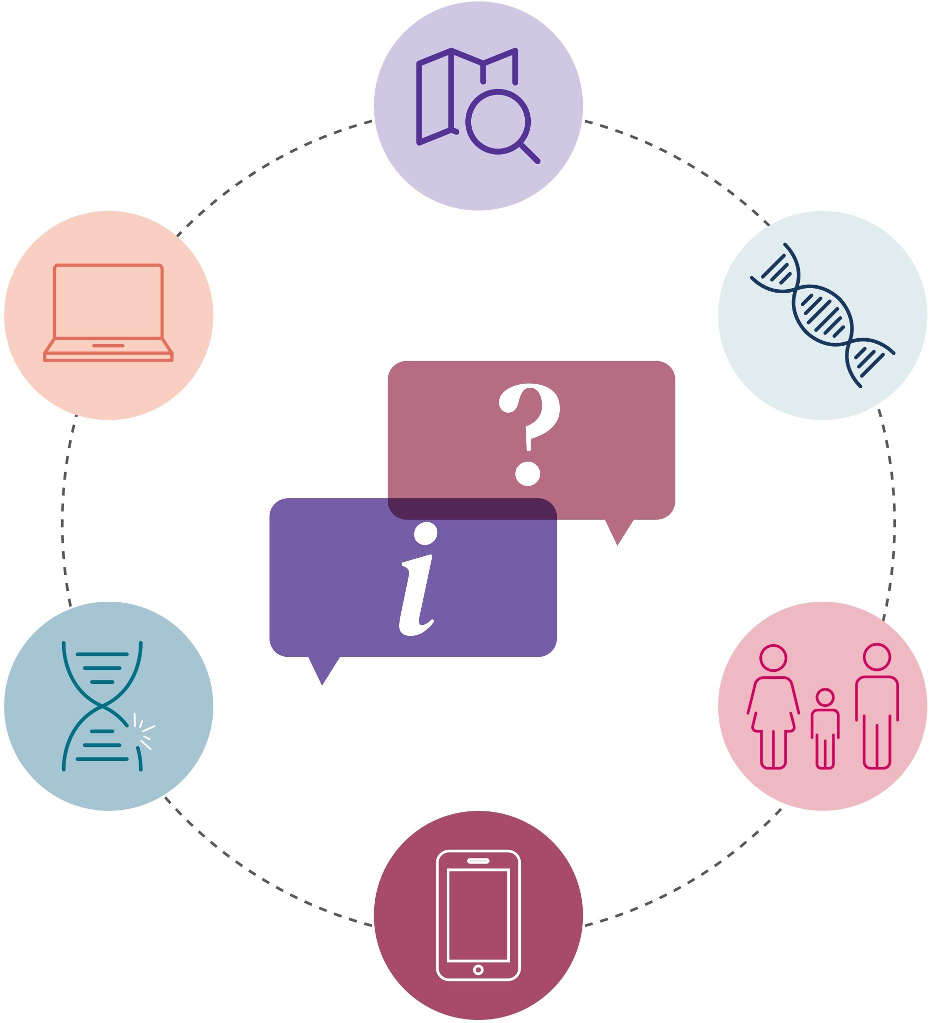

Further information

You may want to discuss the following points:

- Where the patient can find further information
- Who is best to contact if the patient wants to talk to someone about gBRCA testing
- Any written information sheets you are giving the patient to take home with them
- Any patient support groups the patient may be interested in

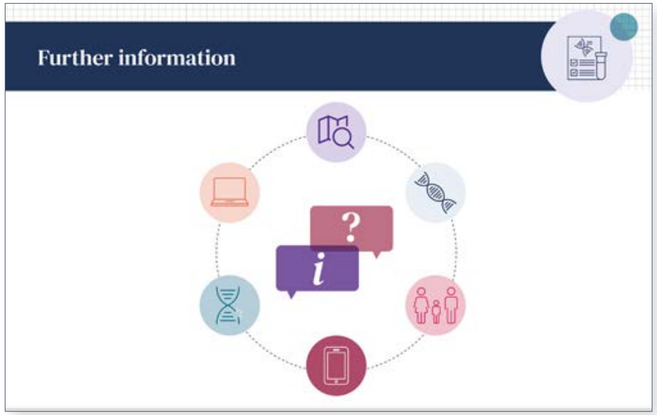

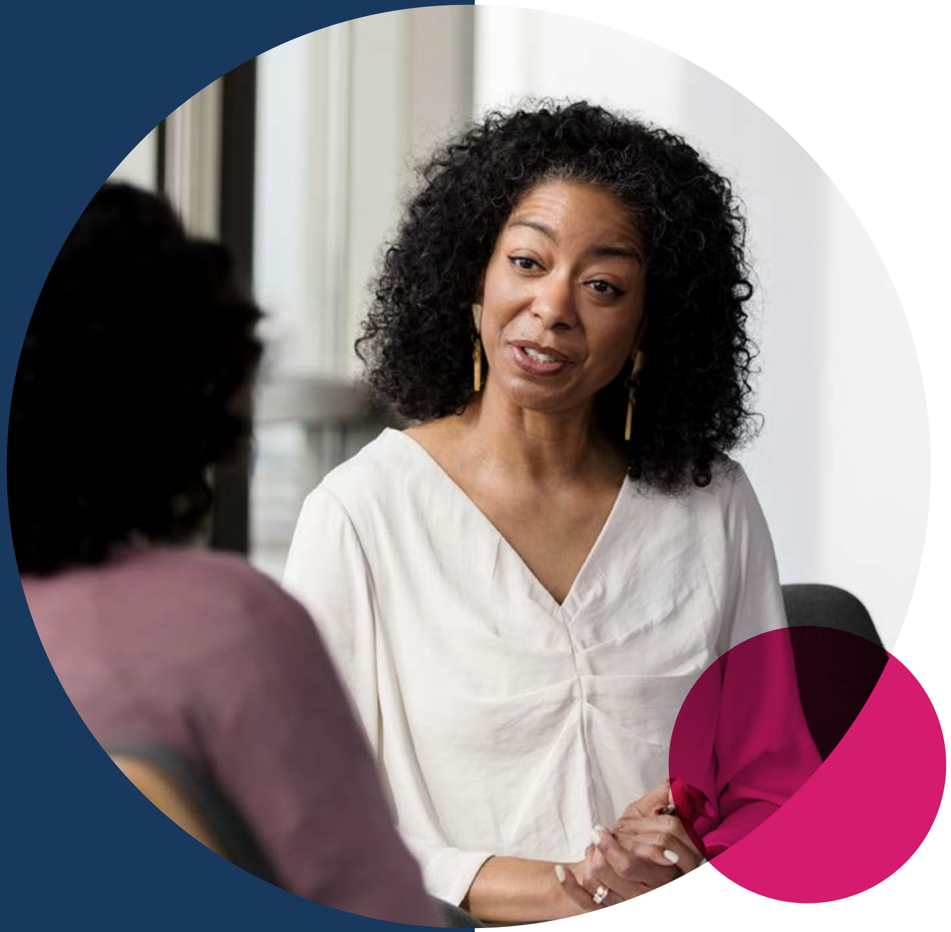

Supplement: Supplementary file 1 — Supplementary information [file 41523_2024_686_MOESM1_ESM.pdf]
